# Enhanced Rishirilide Biosynthesis by a Rare In-Cluster Phosphopantetheinyl Transferase in Streptomyces xanthophaeus

**DOI:** 10.1128/spectrum.03247-22

**Published:** 2022-11-03

**Authors:** Songya Zhang, Shuai Fan, Jing Zhu, Liying Zhou, Xiaohui Yan, Zhaoyong Yang, Tong Si, Tao Liu

**Affiliations:** a CAS Key Laboratory of Quantitative Engineering Biology, Shenzhen Institute of Synthetic Biology, Shenzhen Institute of Advanced Technology, Chinese Academy of Sciences, Shenzhen, China; b The Institute of Medicinal Biotechnology, Chinese Academy of Medical Sciences and Peking Union Medical College, Beijing, China; c Department of Natural Products Chemistry, School of Pharmacy, China Medical University, Shenyang, China; d State Key Laboratory of Component-based Chinese Medicine, Tianjin University of Traditional Chinese Medicine, Tianjin, China; Georgia Institute of Technology

**Keywords:** rishirilide, *Streptomyces*, phosphopantetheinyl transferases, biosynthesis

## Abstract

Phosphopantetheinyl transferases (PPTases) play important roles in activating *apo*-acyl carrier proteins (*apo*-ACPs) and *apo*-peptidyl carrier proteins (*apo*-PCPs) in both primary and secondary metabolism. PPTases catalyze the posttranslational modifications of those carrier proteins by covalent attachment of the 4′-phosphopantetheine group to a conserved serine residue. The protein-protein interactions between a PPTase and a cognate acyl or peptidyl carrier protein have important regulatory functions in microbial biosynthesis, but the molecular mechanism underlying their specific recognition remains elusive. In this study, we identified a new rishirilide biosynthetic gene cluster with a rare in-cluster PPTase from Streptomyces xanthophaeus no2. The function of this Sfp-type PPTase, SxrX, in rishirilide production was confirmed using genetic mutagenesis and biochemical characterization. We applied molecular modeling and site-directed mutagenesis to identify key residues mediating the protein-protein interaction between SxrX and its cognate ACP. In addition, six natural products were isolated from wild-type *S. xanthophaeus* no2 and the Δ*sxrX* mutant, including rishirilide A and lupinacidin A, that exhibited antimicrobial and anticancer activities, respectively. SxrX is the first Sfp-type PPTase identified from an aromatic polyketide biosynthetic gene cluster and shown to be responsible for high-level production of rishirilide derivatives.

**IMPORTANCE** Genome mining has been a vital means for natural product drug discovery in the postgenomic era. The rishirilide-type polyketides have attracted attention due to their potent bioactivity, but the poor robustness of production hosts has limited further research and development. This study not only identifies a hyperproducer of rishirilides but also reveals a rare, in-cluster PPTase SxrX that plays an important role in boosting rishirilide biosynthesis. Experimental and computational investigations revealed new insights on the protein-protein interaction between SxrX and its cognate ACP with wide implications for understanding polyketide biosynthesis.

## INTRODUCTION

Phosphopantetheinyl transferases (PPTases) are responsible for phosphopantetheinylation of the acyl carrier protein (ACP) in polyketide synthases (PKSs) and fatty acid synthases (FASs), as well as peptidyl carrier protein (PCP) in nonribosomal peptide synthetase (NRPSs). PPTases catalyze the transfer and covalent attachment of the 4′-phosphopantetheine (PPant) moiety from coenzyme A (CoA) to the conserved serine residue of carrier proteins, which are converted from inactive *apo* forms to active *holo* forms. The PPant moiety of a *holo*-form carrier protein relays acyl or peptidyl intermediates via thioester linkage, which is an important chain-elongating mechanism. In this way, PPTases play an indispensable role for the biosynthesis of fatty acid, polyketide, and NRP products in microorganisms (1, 2). Mining and engineering of PPTases are desirable to identify enzymes that act on diverse substrates of PKS or NRPS pathways (3).

PPTases are prevalent in bacteria, fungi, and animals. PPTases can be broadly categorized into three classes based on sequence, structure, and substrate specificity (4). The type I PPTases (ACP synthase [ACPS]-type PPTases) are present in the genome sequences of almost every bacterium, are typically 120 amino acids in length, and function as homotrimers. ACPS-type PPTases activate *apo*-ACPs from polyketide and fatty acid pathways but do not take PCPs. The type II PPTases (Sfp-type PPTases) act on both PCP and ACP domains and consist of one monomer that is about twice the size of one type I PPTase subunit. The prototype PPTase from Bacillus subtilis for surfactin biosynthesis belongs to this group. Type III PPTases are often a C-terminal domain of fungal FASs, catalyzing self-phosphopantetheinylation of the ACP domain. This type of PPTases also resides in fungal PKSs (5, 6).

PPTases normally exhibit broad substrate specificities and act on diverse ACPs. For example, Streptomyces coelicolor produces 22 secondary metabolites, but its genome only encodes three PPTases (SCO5883, SCO667, and SCO4744). Two of these proteins are Sfp-type enzymes (SCO5883 and SCO667), and the third one (SCO4744) belongs to the ACPS category (7). In S. coelicolor, no discrete PPTase is associated with the actinorhodin polyketide synthase, and SCO4744 (ScACPS) plays a dual role by activating both the fatty acid synthase and actinorhodin polyketide synthase ACPs. By contrast, some PPTases are specific to their cognate carrier proteins. For instance, the biosynthesis of jadomycin employs a dedicated PPTase, JadM, within the type II PKS pathway (8).

Rishirilide is an aromatic polyketide discovered in Streptomyces rishiriensis OFR-1056 in 1984. It is also produced by a few other *Streptomyces* species, including Streptomyces bottropensis and Streptomyces olivaceus SCSIO T05 (9, 10). Rishirilide is an α_2_-macroglobulin inhibitor with potential applications in the prevention and treatment of thrombosis caused by fibrinolytic accentuation (11). Also, rishirilide B inhibits glutathione *S*-transferases, which mediate increased resistance toward anticancer drugs, and hence may serve as a potential anticancer agent (12). Rishirilides are synthesized by a type II PKS biosynthetic gene cluster (BGC) (13), and their assembly mechanism has been partially elucidated (13). Interestingly, previously reported rishirilide BGCs (*sxd* from *S. olivaceus* SCSIO T05 or *rsl* from S. bottropensis) do not harbor any PPTase-encoding genes (9, 13, 14). The *rsl* BGC was cloned into cosmid cos4 and conferred production of rishirilide in the surrogate host Streptomyces albus J1074, suggesting that the host PPTase(s) can activate the *rsl* ACP (15).

In this study, we identified four rishirilide derivatives (rishirilides A, B, and C and lupinacidin A) produced by the newly isolated Streptomyces xanthophaeus no2. Bioinformatic analysis revealed a corresponding gene cluster that contained an in-cluster PPTase-encoding gene, *sxrX*, which was unprecedented for known rishirilide BGCs. Gene deletion of *sxrX* abolished rishirilide production, and biochemical assays confirmed the PPTase activity and substrate selectivity of SxrX on its cognate *apo*-ACP SxrK1. The key amino acid residues mediating the specific protein-protein interaction of SxrX and SxrK1 were determined using molecular docking and site-directed mutagenesis.

## RESULTS

### *S. xanthophaeus* no2 as a new producer of bioactive rishirilides.

**FIG 1:**
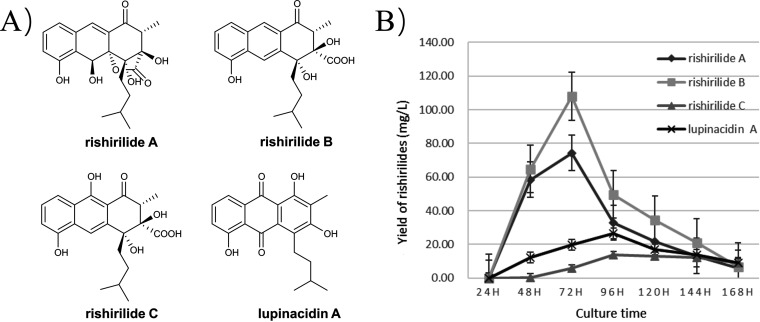
Analysis of the rishirilide derivatives from *S. xanthophaeus* no2. (A) Secondary metabolites isolated from crude extracts of *S. xanthophaeus* no2. (B) Production of rishirilides A, B, and C and lupinacidin A in wild-type *S. xanthophaeus* no2. All cultures were fermented in flasks in optimized GYM medium in triplicate.

The redox enzymes RslO8 and RslO9 are highly conserved in rishirilide BGCs (13), and a high-throughput PCR typing assay targeting *rslO8* and *rslO9* genes was developed to survey an in-house *Streptomyces* strain collection for new rishirilide producers. For one particular strain hit, *S. xanthophaeus* no2, liquid chromatography–high-resolution mass spectrometry (LC-HR-MS) results revealed production of rishirilide derivatives (Fig. 1). Purified compounds were obtained following fermentation in glucose-yeast extract-malt extract (GYM) medium and serial chromatographic separation. These compounds were identified as rishirilide A, rishirilide B, rishirilide C, and lupinacidin (Fig. 1; see also Fig. S1 to S15 in the supplemental material), based on comparison of nuclear magnetic resonance (NMR) and MS results with reported data (9, 15, 16). The time courses of rishirilide production in shake flasks were quantified by LC-tandem MS (MS/MS) (Fig. 1B). Whereas rishirilide A and B titers peaked at 72 h, accumulation of rishirilide C and lupinacidin plateaued after 96 h (Fig. 1B).

We further examined the bioactivities of rishirilide molecules produced from *S. xanthophaeus*. It has been reported that rishirilides exhibit a wide range of bioactivities (9, 17, 18). In particular, the *in vitro* antibacterial assay confirmed the antagonistic effects of rishirilide A and lupinacidin A against the pathogenic bacterium Staphylococcus aureus (Fig. S16). In addition, rishirilide A exhibited antiproliferative activity against PC3, A549, and HeLa cell lines, with 50% inhibitory concentration (IC_50_) values of 15.67 ± 1.30 μM (mean ± standard deviation), 24.7 ± 4.1 μM, and 10.4 ± 0.8 μM, respectively. Lupinacidin A showed a slightly different inhibitory spectrum, exhibiting IC_50_ values of 11.37 ± 0.49 μM and 8.92 ± 0.93 μM when tested against A549 and HeLa cells, respectively (Fig. S17). Together with structural elucidation, we confirmed *S. xanthophaeus* no2 as a new robust producer of bioactive rishirilides.

### Genome sequencing and bioinformatic analysis of rishirilide BGC.

The strain *S. xanthophaeus* no2 was identified based on morphology and 16S rDNA sequence analysis, as described in the supplemental material. To understand the biosynthetic potential of *S. xanthophaeus* no2, its complete genome was sequenced using a combination of BGISeq and PacBio technology. The assembled genome of *S. xanthophaeus* no2 is 8,324,019 bp in length and consists of a linear chromosome with a mean GC content of 71.32%. The genome contains 7,115 protein-coding sequences, 21 rRNA genes, and 78 tRNA genes identified through bioinformatics analysis. Table S1 provides the overview of the genomic features of this strain.

**FIG 2:**
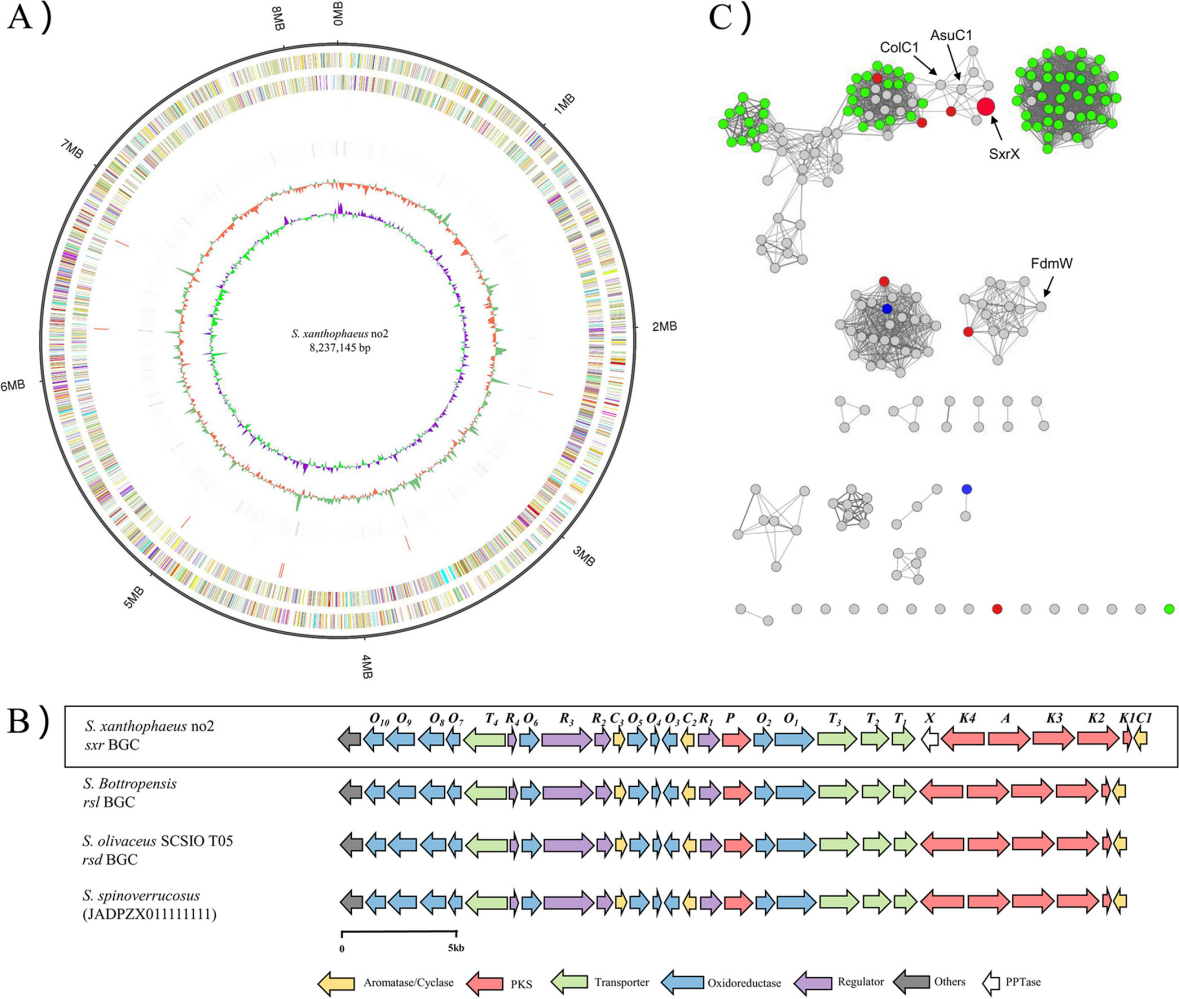
Genome sequencing and analysis of strain *S. xanthophaeus* no2. (A) Complete genome of *S. xanthophaeus* no2. The five circles (outer to inner) represent forward-strand CDSs, reverse-strand CDSs, nomenclature, and locations of predictive secondary metabolites generated using antiSMASH 3.0 software, with GC content and GC skew. (B) Schematics of the organization of rishirilide BGC in the reported producing strains. (C) Sequence similarity network constructed for the protein sequence of SxrX and its homologs. The other six PPTases in *S. xanthophaeus* no2 are labeled in red, PPTases from type II PKS clusters are shown in green, and PPTase from *S. albus* J1074 is shown in blue. The determined *E* value was set at 10^−85^ when separating members of different PPTase families.

The biosynthetic pathway of rishirilide was previously investigated in *S. bottropensis* and *S. olivaceus* (9, 13). Here, we have proposed a tentative BGC of rishirilides (the *sxr* BGC) based on bioinformatic analysis of the *S. xanthophaeus* no2 genome. The proposed rishirilide BGC (~40 kb) consists of 35 open reading frames at the subtelomeric region of the genome; this BGC exhibits high similarity in both sequences and cluster organization with the published rishirilide BGCs, suggesting a similar biosynthetic mechanism (Fig. 2). The core part of the *sxr* gene cluster contains three genes encoding the minimal polyketide synthetase (*sxrK1*, *sxrK2*, and *sxrK3*) responsible for the polyketide backbone assembly. Three aromatases (SxrC1 to -C3) are supposed to assist in the cyclization of the polyketide chain. Genes *sxrR1* to *sxrR4* encode pathway-specific transcriptional regulators. The putative 10 redox tailoring enzymes include SxrO1 to SxrO10 (Table S2). Among them, some of the homologous genes in the *rsl* and *rsd* gene cluster have been investigated (9, 13).

Except for an additional PPTase-encoding gene (*sxrX*), this *sxr* cluster is conserved in sequence (~90% overall identity) and organization to the reported rishirilide BGCs (*rsl* and *rsd*). By gene cluster searching in the databases of NCBI and UniProt, while the anthraquinone compound group was discovered in *S. spinoverrucosus* (JADPZX010000001), a rishirilide-like gene cluster was found from this strain with high identity (Fig. 2). However, there is no SxrX homolog located in the gene cluster (19, 20). SxrX exhibited 40% identity with ColC1 (AIL50185.1) from the colabomycin E pathway (21) and 44% identity with AsuC1 (ADI58627.1) from the asukamycin pathway (22).

### Deletion and complementation of *sxrX* and other PPTase-encoding genes.

**FIG 3:**
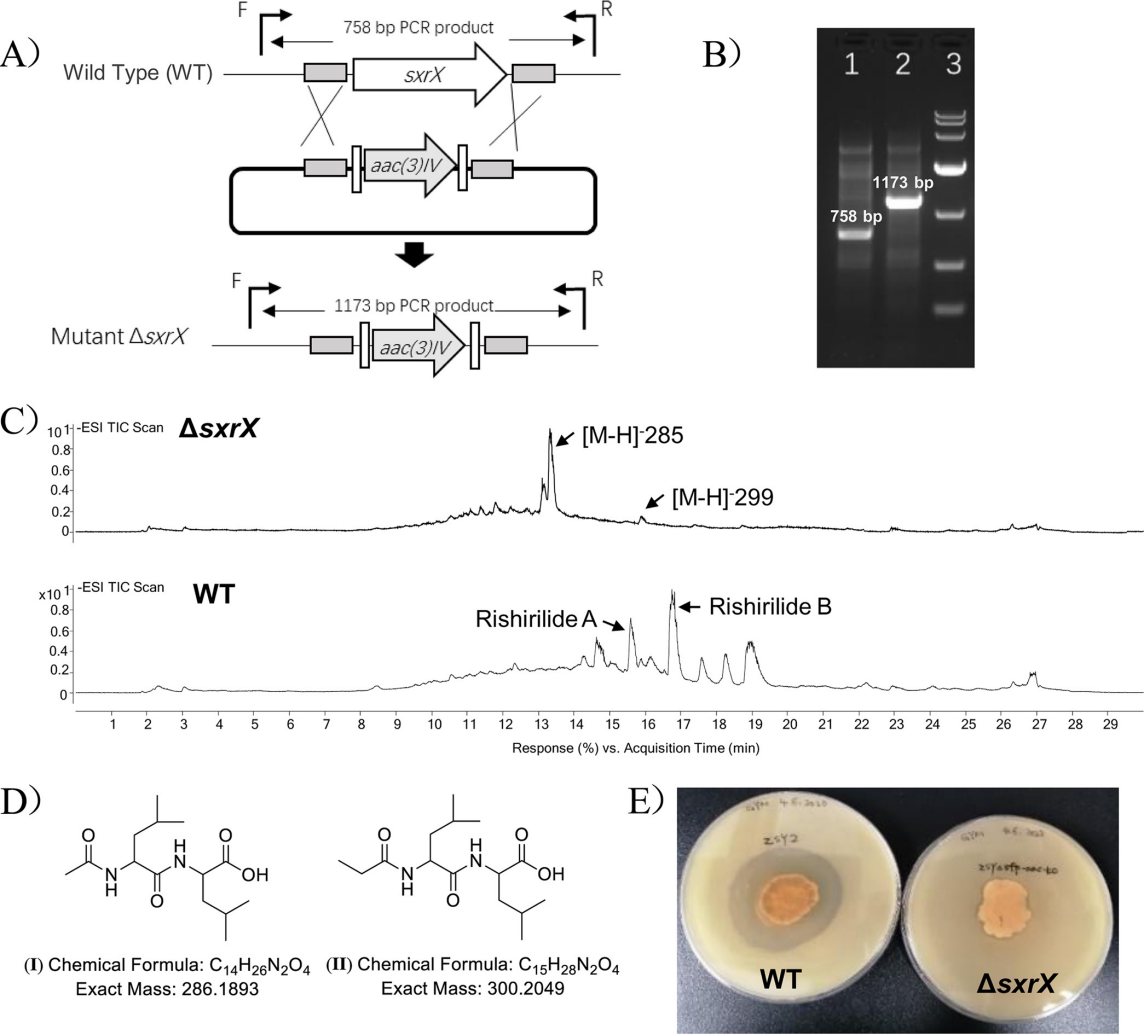
Gene mutagenesis investigation of the gene *sxrX*. (A) Schematics of deletion of the gene *sxrX*. (B) PCR verification for gene deletion of *sxrX*. Note that the samples from left to right represent the PCR product from the wild-type (lane 1), the mutant Δ*sxrX* (lane 2), and the 1-kb marker (lane 3). (C) Comparative LC-MS (total ion chromatogram) profile analysis of the secondary metabolites from strain *S. xanthophaeus* no2 and the mutant *S. xanthophaeus* no2 Δ*sxrX*. (D) The two new secondary metabolites produced in the mutant *S. xanthophaeus* no2 Δ*sxrX*. (E) Comparison of the antibacterial activity of the wild-type and the mutant against S. aureus.

**FIG 4:**
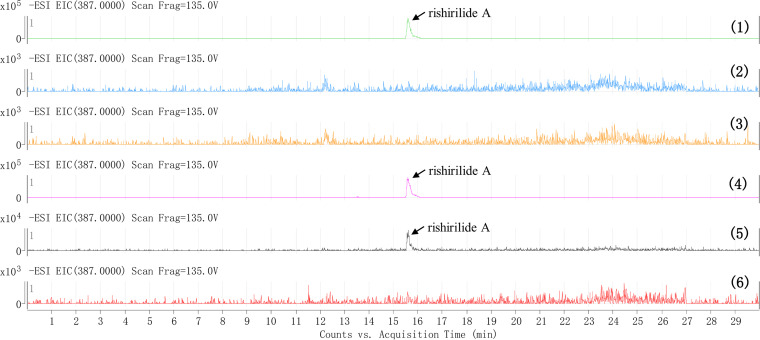
Comparative LC-MS analysis of the secondary metabolite profiles from strain *S. xanthophaeus* no2 and its mutants. LC-MS chromatograms for the mass of rishirilide A ([M-H]^−^ 387) were obtained from the culture of *S. xanthophaeus* no2 (trace 1), *S. xanthophaeus* no2Δ*sxrX* (trace 2), *S. xanthophaeus* no2 Δ*sxrX*::pSET152-SFP-AGI86981 (trace 3), *S. xanthophaeus* no2 Δ*sxrX*::pUWL-H-sxrX (trace 4), *S. xanthophaeus* no2 Δ*sxrX*::pSET152-sxrX (trace 5), and *S. xanthophaeus* no2 Δ*sxrX*::pSET152-SFP-AGI92022 (trace 6).

To verify the function of the gene *sxrX* product, the *S. xanthophaeus* no2 Δ*sxrX* mutant was constructed. We initially disrupted the PPTase-encoding gene *sxrX* by single crossover; to exclude the possible polar effect toward the downstream genes, we also carried out the in-frame deletion of the gene *sxrX*. LC-MS results indicated that the deletion of *sxrX* abolished rishirilide production (Fig. 3), suggesting that *sxrX* is involved in rishirilide biosynthesis. Accordingly, the Δ*sxrX* mutant strain also lost the inhibitory activity against S. aureus (Fig. 3). Surprisingly, two new secondary metabolites were produced in the Δ*sxrX* mutant (Fig. 3), and they were determined to be acetylleucylleucine (I) and propionylleucylleucine (II) (Fig. S18), based on MS and NMR data (Fig. S19-S26). Acetylleucylleucine was first isolated from *S. erythrochromogenes* and considered the degradation product of leupeptin (Fig. S27; Table S3). To further verify the function of the gene *sxrX*, plasmids pSET152-*sxrX* and pUWL-H-*sxrX* for gene complementation were individually introduced into *S. xanthophaeus* no2 *ΔsxrX* to generate *S. xanthophaeus* no2 Δ*sxrX*::pSET152-*sxrX* and *S. xanthophaeus* no2 Δ*sxrX*::pUWL-H-*sxrX*, respectively. As revealed by LC-MS, rishirilide production in the gene complementation mutants was not only restored but also enhanced relative to that of the wild-type (WT) strain (Fig. 4), suggesting that the single *sxrX* gene drives the biosynthesis of the rishirilide variants.

The PPTase-encoding gene is absent in *rsl* and *rsd* (Fig. 2), like many other type II polyketide pathways (e.g., actinorhodin, doxorubicin, mithramycin, and tetracycline) (5). Previously, heterologous expression of the *rsl* BGC in *S. albus* J1074 led to successful reconstitution of rishirilide biosynthesis (15). Therefore, it was postulated that the PPTase(s) from *S. albus* 1074 may be responsible for activating the *apo*-ACP in the rishirilide pathway. By BLASTP search using SxrX as a query sequence, two PPTases (AGI86981 and AGI92022) were identified from *S. albus* 1074. To verify the functions of these PPTases, the mutants *S. xanthophaeus* no2 Δ*sxrX*::pSET152-SFP-AGI86981 and *S. xanthophaeus* no2 Δ*sxrX*::pSET152-SFP-AGI92022 were constructed. The LC-MS analysis showed that the complementation of PPTases from *S. albus* J1074 did not restore rishirilide biosynthesis (Fig. 4), suggesting dedicated catalytic specificity between SxrX and its intrinsic substrate ACP in the *sxr* pathway.

### *In vitro* phosphopantetheinylation assay of SxrX.

The substrate selectivity of SxrX was further investigated using *in vitro* biochemical assays. The SxrX protein was recombinantly expressed and purified from Escherichia coli BL21(DE3)/pET21b-sxrX(opt). The optimal pH for SxrX activities was determined to be 7.5 in 75 mM morpholinoethanesulfonic acid and 100 mM Tris-HCl buffer. In addition, the SxrK1 protein was recombinantly expressed in E. coli BL21(DE3)/pET28a-*SxrK1*. An N-terminal His tag was included to assist purification by affinity and size exclusion chromatography. The PCP_ws9326_ protein, which is a peptidyl carrier protein from the biosynthetic pathway of WS9326a, and the ACP from E. coli (EcACP) were expressed and purified as previously reported (23) as control substrates (Fig. S28).

**FIG 5:**
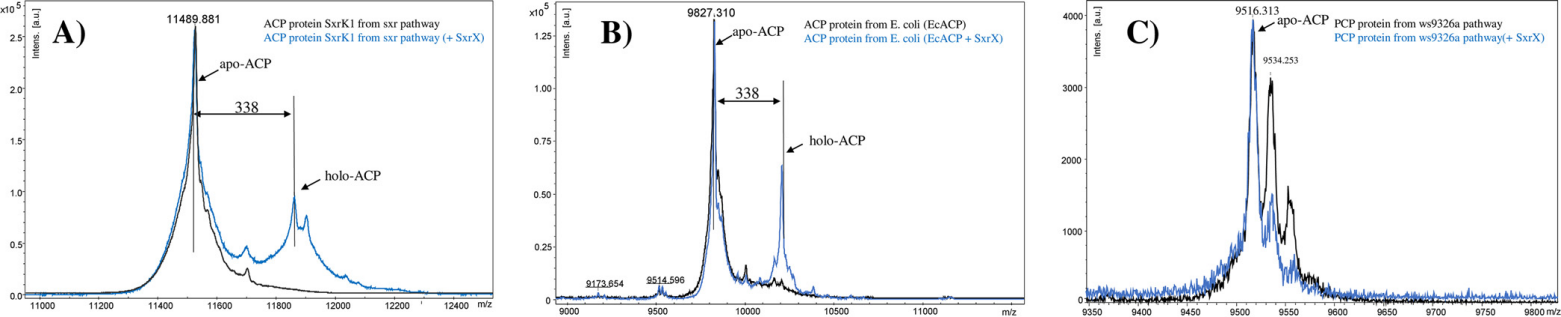
MALDI-TOF MS analysis results of the *in vitro* phosphopantetheinylation assay of SxrX.

In the presence of SxrX, SxrK1, PCP_ws9326_, and EcACP were individually incubated with CoA, and the reaction mixtures were subsequently analyzed by MALDI-TOF MS to monitor the *m/z* values of corresponding protein [M + H]^+^ ions. Upon SxrX treatment, new peaks with a mass shift of 338 Da relative to the *apo* forms were observed for SxrK1 (Fig. 5A) and EcACP (Fig. 5B), whereas no new peaks were observed for PCP_ws9326_ (Fig. 5C), indicating that SxrX has distinct phosphopantetheinylation activities toward different ACPs or PCPs.

### RT-PCR analysis.

Bioinformatic analysis revealed 7 PPTase-encoding genes in the *S. xanthophaeus* no2 genome, including *sxrX* (ctg007-1006), ctg016-1818, ctg022-2273, ctg032-2936, ctg040-3497, ctg046-3754, and ctg073-4969 (Fig. S29). To examine the possible roles of other PPTases in rishirilide biosynthesis, reverse transcription-PCR (RT-PCR) analysis was performed using the WT strain and the Δ*sxrX* mutant of *S. xanthophaeus* no2 (Fig. S30). In the WT strain, *sxrX*, ctg016-1818, and ctg046-3754 were actively transcribed. By contrast, no PPTase genes were actively transcribed in the Δ*sxrX* mutant. Deletion of *sxrX* affected the transcription of other PPTase-encoding genes (ctg016-1818 and ctg046-3754) in the resulting mutant (Fig. S30). These results suggested that this functional PPTase SxrX is necessary for the biosynthesis of rishirilide in this strain.

### Gene cluster neighborhood and SSN analysis.

Homology search in the genome database was performed using SxrX as a query protein. In total, 188 gene clusters were discovered, and 65 PPTase-encoding genes were located inside the gene clusters for type II PKS biosynthesis (Fig. S31). A PPTase family network was constructed based on the functionally characterized PPTases from *Streptomyces*. SxrX and another 572 PPTases were aligned and clustered, and the sequence similarity network (SSN) was constructed using the protein sequence. Eighty-eight PPTases were chosen from type II PKS BGCs, two PPTases were selected from *S. albus* 1074, and others were obtained from annotated gene clusters (Fig. 2). The analysis showed that SxrX is positioned at the same clade with other homologues situated in type II PKS clusters (Fig. 2). The PPTases from *S. albus* J1074 displayed a distant relationship with SxrX. These similarities suggest the specificity of the sequence-function relationships.

### Phylogenetic analysis of the protein SxrX.

**FIG 6:**
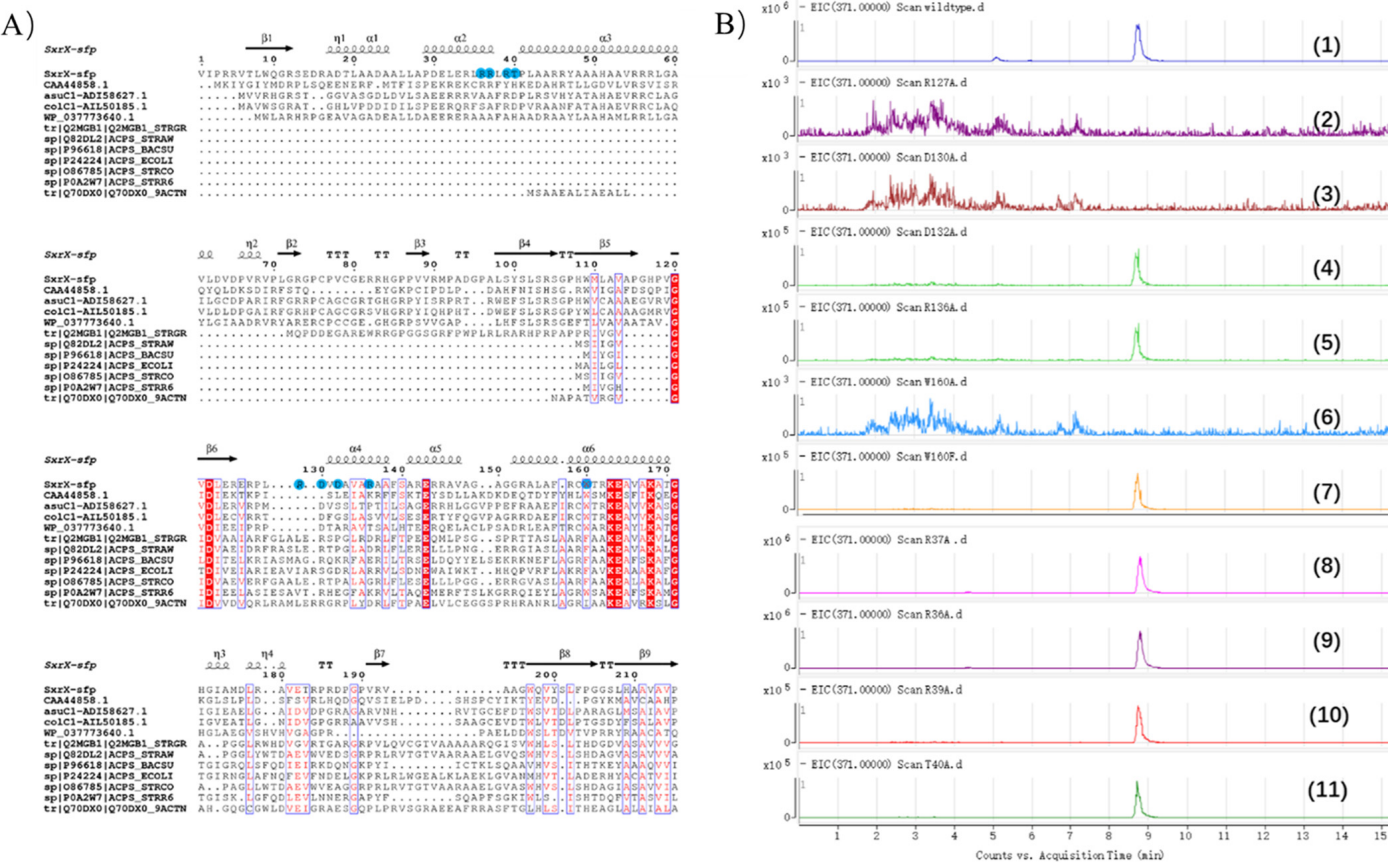
Putative SxrX active site mutants constructed for *in vivo* analysis. (A) Alignment of SxrX sequence with other homologs, showing the positions explored through mutation (R36, R37, R39, T40, R136, D130, D132, R127, and W160 changed to alanine residue, marked in blue circle). The graphics for sequence alignment were constructed using BLAST Omega and ESPript (48). (B) LC-MS chromatograms monitoring for the mass of rishirilide B ([M-H]^−^ 371, EIC scan mode) were individually obtained from the cultures of wild-type (1), sxrX-R127A (2), sxrX-D130A (3), sxrX-D132A (4), sxrX-R136A (5), sxrX-W160A (6), sxrX-W160F (7), sxrX-R37A (8), sxrX-R36A (9), sxrX-R39A (10), and sxrX-T40A (11).

FdmW is the first ACPS-type (FxxKEAxxK) PPTase identified from the BGC of the polyketide fredericamycin (5). By contrast, the conserved motif in SxrX is WxxKEAxxK, the alignment of SxrX with reported PPTases showed that SxrX has three conserved motifs found in the Sfp-type PPTase family motifs P1(PxxP), P2(GxD), and P3 [(F/W)(S/T/A)xKE(S/A)xxK] (24). Based on this principle, SxrX was categorized in the Sfp-type PPTase family (25) (Fig. 6 and Fig. S32).

### Protein-protein docking analysis.

The protein-protein recognition between ACP and PPTase is elusive in the *sxr* pathway. Exploring the molecular determinants of specific PPTase-ACP binding can be helpful in unveiling the evolutionary development of PPTase proteins. Protein structure prediction and molecular dynamic (MD) simulation were carried out using AlphaFold2 to position the amino acid residues related to the protein-protein interaction between SxrX and SxrK1. The SxrX model was highly similar to the reported PPTase (26, 27) and consisted of two domains, each spanning ~100 residues. Both domains generated the expected central antiparallel β-sheet, with a β1-β3-β2 arrangement. The most prominent differences in comparison with the known PPTase structure (PDB ID 4MRT) were the orientation of α1-α2 (K110-Q139) and the loop region containing β1-β2 (G158-S187) at the C terminus. The ACP proteins from rishirilide BGC *sxr* and *rsl* are highly similar (88% identity). The model alignment for those two ACP structures with their homologs showed that the key residues at the interface with the PPTase protein were highly conserved (Fig. S33). The protein sequence of the SxrX-SxrK1 complex was made by adding the (GGGGGG)_10_ linker for flexibility directly between SxrX and SxrK1, and this complex structure was subsequently constructed by using the AlphaFold2 program (28). The (GGGGGG)_10_ linker was then removed from the PPTase-ACP complex structure, which was used for further structure optimization using MD simulations. After a 100-ns simulation analysis, the superposition of the crystal structures of the Sfp-PCP complex (PDB ID 4MRT) and the SxrX-SxrK1 complex yielded a root mean square deviation value of 3.852 Å (Fig. S34).

**FIG 7:**
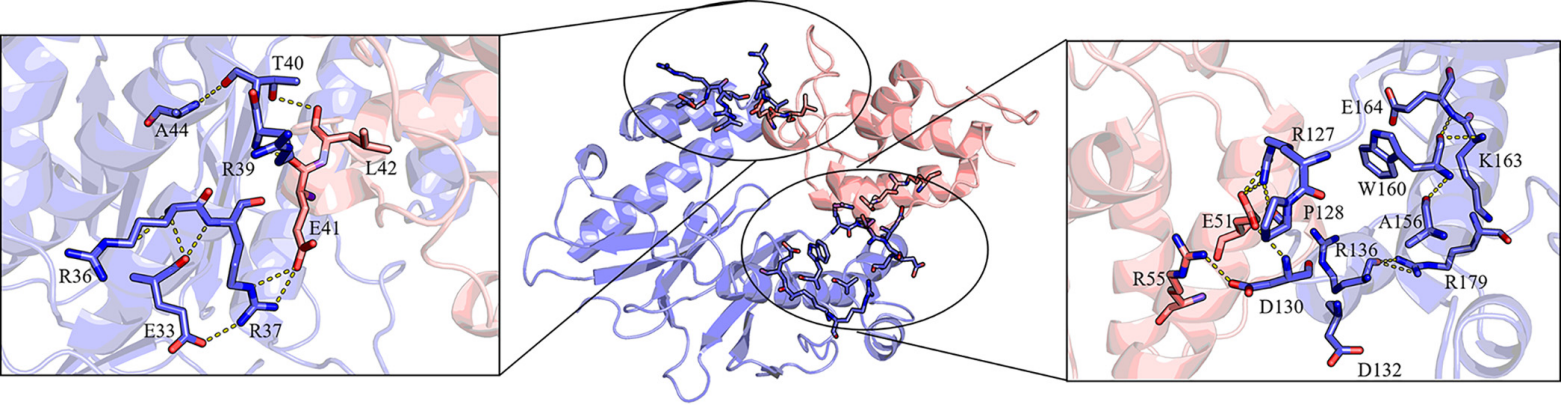
Protein docking result for the PPTase (SxrX) to its cognate ACP (SxrK1) from the *sxr* BGC. Residues labeled are the possible sites that interacted with PPTase protein. SxrX and SxrK1 are shown in blue and pink, respectively. The key amino acids are labeled and shown in the stick model. The dotted lines indicate hydrogen bonding.

A possible protein-protein interaction mode between PPTase (SxrX) and ACP (SxrK1) was proposed based on AlphaFold2 models. The direct interaction surface included the α2-helix at the N-terminal domain, which was relatively longer than FdmW (Fig. S32), and the α5-helix at the C terminus with the preceding loop. Among them, 8 residues (R36, R37, R39, T40, R127, D130, D132, and R136) were predicted to be involved in the interaction. Specifically, the guanidino groups on the R37 residue of SxrX formed hydrogen bond interactions with E33-SxrX and E41-SxrK1. Additionally, hydrogen bonds formed between R36-SxrX and E33-SxrX, as well as the backbones of E41-SxrK1 and L42-SxrK1, with R39-SxrX and T40-SxrX. A net of hydrogen bonds among R127-SxrX, D130-SxrX, and W160-SxrX formed. Further, more expanded interaction site comprised nonpolar contacts between the C-terminal domain of SxrX and the helix of SxrK1 (Fig. 7).

To verify their importance, the individual mutants with protein point mutations of SxrX at the residues were constructed; these were introduced to destroy the possible interaction. The engineered strains with various PPTase mutations were constructed; after that, mutants were cultured and their secondary metabolites were analyzed. Results showed that mutations R36A, R37A, R39A, and T40A did not influence the biosynthesis of rishirilides, suggesting the α2-helix (R36A, R37A, R39A, and T40A) at the N terminal doesn’t interact with ACP. The mutations D130A and R127A abolished the production of rishirilides, suggesting the fifth helix (α5 helix) and the preceding loop (including D130, L129, P128, R127, and E126) in the C-terminal domain may form the key interaction with SxrK1. Interestingly, the D132A and R136A mutants still produced the rishirilides, which could be explained by the outward orientation of residues in the helix (Fig. 6).

### MD simulations for protein-protein interface changes of the mutants.

To probe the mechanism of amino acid substitutions affecting the activity, the MD simulation was performed to determine the binding free energy, confirmation, and intermolecular interaction of SxrX with SxrK1. First, the binding free energy between SxrX and SxrK1 for different systems were tabulated (Table 1). In detail, the mutations R127A and W160A caused a decrease in binding energy. Consistent with *in vitro* enzyme activity results, the mutations R127A and W160A abolished the production of rishirilides completely. Interestingly, the D130A mutation of SxrX, which disrupted the hydrogen bond with R55 of SxrK1, had no effect on SxrX binding to SxrK1 (Table 1). However, it was confirmed that the corresponding D130A mutant strain was unable to produce rishirilides. Although this mutation site was distant from S46-SxrK1, which is at the N terminus of helix α3, the impact of the mutation led to the shift of helix α3. Then, a conformational change of the active site S46-SxrK1 could affect the correct substrate interaction in the active site (Fig. S35).

**TABLE 1:** Binding free energy between SxrX and its partner, SxrK1

SxrX form	Binding free energy (kcal/mol)
WT	−39.37
R127A	0.43
D130A	−40.46
W160A	−26.07

**FIG 8:**
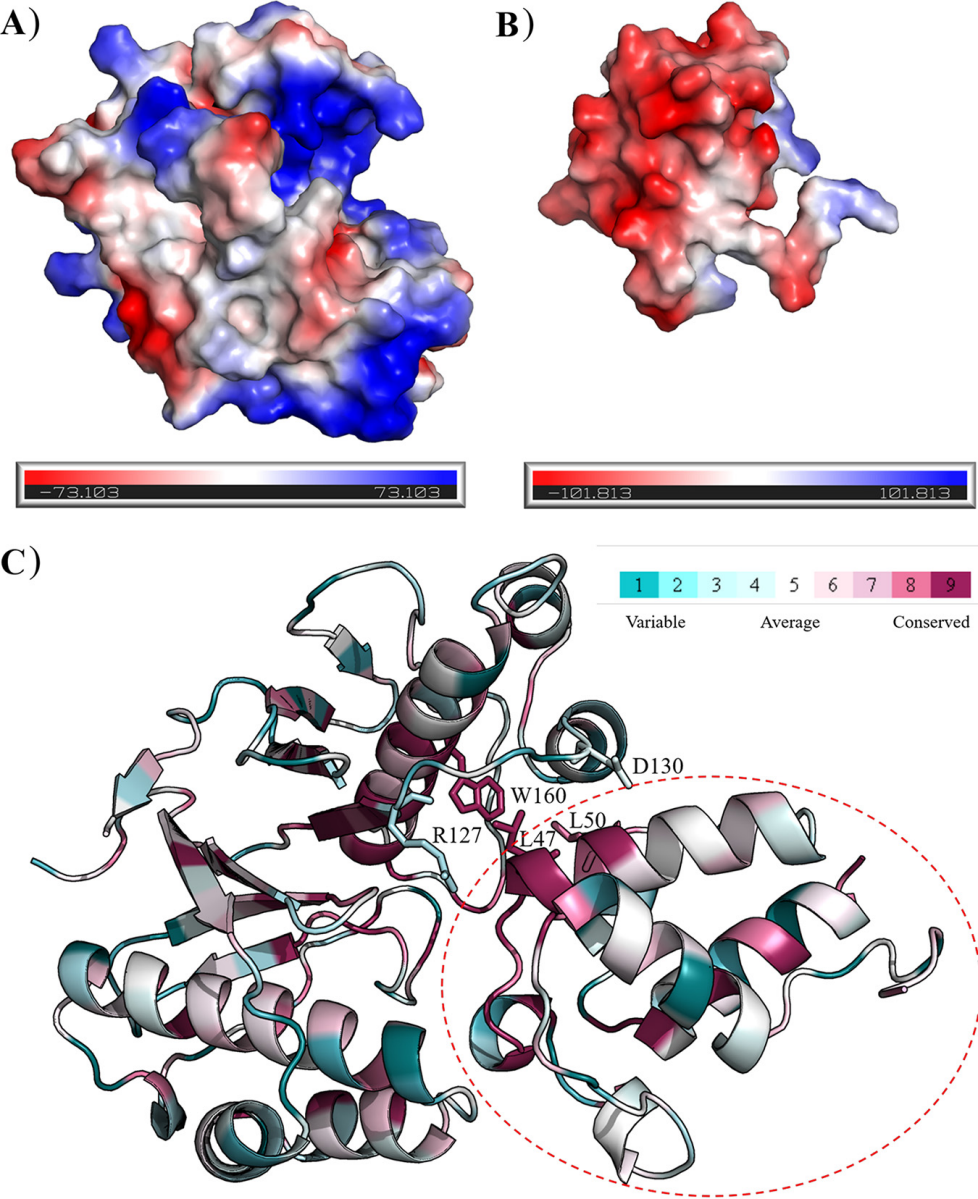
Properties of SxrX and SxrK1. (A and B) Electrostatic surface potentials of SxrX (A) and SxrK1 (B) were determined. (C) Conservative analysis of the SxrX-SxrK1 complex. The red dashed circle indicates SxrK1. The key amino acids are labeled and shown in the stick model.

As shown in Fig. 8A, the electrostatic surface potential of the shallow pocket of SxrX was positive (shown in blue), with a corresponding SxrK1 showing a significant amount of negatively charged surface potential (Fig. 8B, shown in red). Similarly, the R127A and D130A mutations could effectively block rishirilide production, implying those mutants might prevent SxrK1 binding to SxrX. The 160th amino acid residue (W or F) was conserved among the Sfp-type PPTase family (Fig. 8C), and mutation of W160A led to the abolishment of rishirilide biosynthesis, whereas the mutation W160F did not influence the production (Fig. 6). This was possibly due to the fact that the bulky phenylalanine or tryptophan aromatic ring was larger than the alanine side chain and may have therefore caused appropriately hydrophobic stacking during the interaction of SxrX with SxrK1. Also, L47 and L50 of SxrK1, which are involved in the hydrophobic interaction with W160-SxrX, were conserved through *apo*-acyl carrier proteins (Fig. 8C). Therefore, it was likely that both types of hydrophobic interactions, hydrogen bond interactions and hydrophobic interactions, play key roles in the binding of SxrK1 to SxrX. Interestingly, W160-SxrX, L47-SxrK1, and L50-SxrK1 showed a remarkably high level of amino acid sequence conservation. This indicated that hydrophobic interactions of those amino acid residues are commonly observed in PPTase and its ACP. Additionally, R127 and D130 of SxrX were nonconserved sites (Fig. 8C) which were involved in the H-bond network with E51 and R55 of SrxK1. Notably, the H-bond network may not be ubiquitous in the Sfp-type PPTase family. Hence, SxrX has a specific interaction with SxrK1, which is influenced in part by electrostatic interactions.

## DISCUSSION

The ratio of *apo*- and *holo*-ACP was directly linked to the activity of the PPTase enzyme. Horizontal gene transfer between species and “inventive evolution” could be an explanation for this gene loss or presence in different species, which may be related with the regulation of the production of interior secondary metabolites in the organism (29, 30). Therefore, we postulated that the presence or loss of the PPTase-encoding gene in different rishirilide-producing strains could be relevant for the global regulation of the physiological and secondary metabolism.

Sequence analysis showed the existence of the rare codon (UUA) in gene *sxrX*. The UUA codon also exists in SxrR3 (a SARP family transcriptional regulator) and SxrT4 (a drug resistance transporter) in the *sxr* cluster; therefore, it was postulated that the transcription of rishirilide in this *S. xanthophaeus* no2 strain is mediated by the AdpA regulatory system (31) and SxrX might also be involved in the physiological regulation of this strain during its growth and development, leading to the its metabolic changes (32).

In conclusion, we identified a new BGC from a new rishirilide producer: Streptomyces xanthophaeus no2. Four rishirilide derivatives were identified, and bioactivity assays demonstrated that rishirilide A had significant inhibitory activity against Staphylococcus aureus and cytotoxic activity against tumor cells. Through gene mutagenesis experiments, protein biochemical investigation, docking, and MD simulation analysis, we characterized a specific PPTase, SxrX, which catalyzed the phosphopantetheinylation of the ACP SxrK1 in this rishirilide biosynthesis pathway. This is contrary to previous reports indicating that most phosphopantetheinyl transferases are flexible and have broad-range catalytic activities during postmodification of carrier proteins (7, 33, 34). Upon further study, SxrX as a phosphopantetheinyl transferase was proven to be specific to the activation of its cognate substrate, ACP(SxrK1), in *S. xanthophaeus* no2. Biochemical characterization of SxrX also showed that SxrX can phosphopantetheinylate other peptidyl and acyl carrier proteins. Protein mutational analysis and MD simulation analysis suggested that the specific interaction region between SxrX and ACP(SxrK1) is located at the fifth helix and the preceding loop at the C-terminal domain of SxrX. These findings provide the groundwork for future genetic engineering of novel antibiotic biosynthesis pathways and offer a rare example of specialized phosphopantetheinylation during polyketide building.

## MATERIALS AND METHODS

### General methods.

All antibiotics and medium components used in this study were purchased from Oxoid and Sigma-Aldrich. Restriction enzymes, T4 DNA ligase, and NEBuilder HiFi DNA assembly master mix were bought from NEB Biotechnology Co., Ltd. Plasmid, gel purification, and cycle pure kits were acquired from Axygen. Primer synthesis and DNA sequencing were performed by GeneWiz Co., Ltd.

### Strains, plasmids, and culture conditions.

Primers, plasmids, and strains used in this study are described in Tables S4 to S6. E. coli DH5α was cultivated in liquid or on solid LB medium at 37°C, overnight. E. coli ET12567 (pUZ8002) or ET12567 (pR9406) was used for conjugation. E. coli BW25113::pKD46 was used for λ Red recombination. Streptomyces cells were cultivated in liquid tryptic soy broth (TSB) medium and fermented in GYM medium at 28°C. The working concentrations of antibiotics were as follows: ampicillin (100 μg/mL), apramycin (50 μg/mL), chloramphenicol (25 μg/mL), kanamycin (50 μg/mL), fosfomycin (200 μg/mL), and hygromycin (100 μg/mL).

### Fermentation and isolation of rishirilide.

After incubating on an MS agar plate at 28°C for 3 days, the strains were scraped and inoculated into 250-mL Erlenmeyer flasks containing 50 mL of liquid TSB medium with 50 μL apramycin (50 mg/mL). For optimization of fermentation conditions, the flasks were incubated at 28°C with shaking (220 rpm) for 2 days to produce seed cultures. The seed culture (1 mL) was inoculated into a 500-mL baffled flask containing 150 mL of liquid GYM medium consisting of glucose (4 g/liter), yeast extract (4 g/liter), and maltose extract (10 g/liter) in 1 liter of double-distilled H_2_O, with the pH adjusted to 7.2 with 1 M NaOH. The flasks were incubated at 28°C with shaking (220 rpm) for 3 days.

Large-scale fermentation was performed for rishirilide purification; for the 15-liter fermentation, 3 g crude extract was collected by ethyl acetate extraction. The crude extract was subjected to an SPE column, 30% and 100% methanol were used to elute the column, and the resulting elution was collected. According to the LC-MS analysis, the main target components were collected in the fraction of 100%. The fraction was then subjected to preparative HPLC (30%, 60%, 80%, and 100% methanol, 18 mL/min; 5 μm, 250- by 20-mm column) to get nine subfractions. The subfractions 3, 4, 5, and 8 were separated by semipreparative HPLC (5 μm with 250- by 20-mm column or 3.5 μm with 100- by 4.5-mm column) by gradient elution (methanol-H_2_O) to generate rishirilide A (20 mg), rishirilide B (2 mg), rishirilide C (3 mg), and lupinacidin A (6 mg), respectively.

### NMR and high-resolution MS analysis.

NMR was employed to elucidate the structures. The one-dimensional (1D) NMR spectra [^1^H NMR (400 MHz or 600 MHz) and ^13^C NMR (100 MHz or 150 MHz)] and 2D NMR spectra (^1^H/^1^H correlation spectroscopy, heteronuclear single quantum coherence, and heteronuclear multiple bond correlation) of these compounds were measured on a Bruker spectrometer in 50 μL dimethyl sulfoxide (DMSO)-*d*_6_ at 35°C or 25°C. Residual solvent signals were used as an internal standard (DMSO-*d*_6_, δ_H_ = 2.5 ppm, δ_C_ = 39.52 ppm; methanol-*d*_4_: δ_H_ = 3.31 ppm, δ_C_ = 49.0 ppm). High-resolution electrospray ionization MS analyses were executed on a UPLC system (Ultimate 3000, Thermo Scientific, Germany) equipped with a Thermo Q Exactive high-field and an Orbitrap Fusion with UltiMate 3000 RSLCnano system (Thermo Scientific). The instrument was equipped with a Kinetex C_18_ column (50 mm by 2.1 mm, 100 Å). A linear gradient analysis from 5% to 100% phase B was performed over 30 min with mobile phase A (H_2_O with 0.1% formic acid) and mobile phase B (acetonitrile with 0.1% formic acid). For each sample, 1 μL was injected onto the column at a flow rate of 0.3 mL/min. The mass spectrometer was programmed to acquire MS/MS results in a data-dependent manner, acquiring five MS/MS scans following each precursor MS_1_ scan. (The percentages of concentrations indicated for HPLC solvents and conditions are volume/volume.) Agilent Ultivo LC-MS was used for sample analysis. A QBH preparative HPLC system was used for compound purification. An Agilent 1260 analytical-preparative HPLC system was used for the sample analysis and purification.

### Antibacterial and cytotoxicity assay.

An antibacterial *in vitro* assay was performed to test the antagonistic effects of the isolated strain *S. xanthophaeus* no2 against pathogenic bacteria, including Escherichia coli, Staphylococcus aureus, Saccharomyces cerevisiae, Fusarium graminearum, Xanthomonas campestris, Botrytis cinerea, Fusarium oxysporum f. sp. cubense race 4, and Pseudomonas syringae pathovar. The antibacterial bioassays were carried out based on the Kirby-Bauer disk diffusion susceptibility test as described elsewhere (35). The methanol solvent was used as a negative control.

Three tested human tumor cell lines, cervical carcinoma (HeLa), lung cancer (A549), and human prostate cancer (PC3), were purchased from the American Type Culture Collection (USA). Each of these cell lines was incubated in Dulbecco’s modified Eagle’s medium or RPMI 1640 containing 10% fetal bovine serum at 37°C under a humidified atmosphere with 5% CO_2_. The cytotoxicity of the isolates toward these tumor cell lines was assessed via the 3-(4, 5-dimethylthiazol-2-yl)-5(3-carboxymethoxyphenyl)-2-(4-sulfophenyl)-2H tetrazolium (Promega, USA) method. Camptothecin was used as a positive control. The cell lines were inoculated into each well of normal 96-well plates and incubated for 12 h before the addition of the test compounds. Different concentrations of each compound were added and exposed to the cells for a continuous cultivation of 48 h. The isolates with inhibition rates of ≥50% against the cell lines were further assessed in triplicate at different concentrations (0.064, 0.32, 1.6, 8, and 40 μM). The IC_50_ values were measured based on Reed and Muench’s method (36). All the experiments were carried out in triplicate.

### Genome sequencing and annotation.

*S. xanthophaeus* no2 cells were collected, and genomic DNA was extracted using the method described by Zhang et al. (37). The whole genome was sequenced using a combination of Illumina Hiseq and Pacific Bioscience SMRT (PacBio RSII) sequencing platforms, with 601-fold average genome coverage. The genome sequencing, assembly, and basic bioinformatics analysis of *S. xanthophaeus* no2 were performed by BGI (Shenzhen). The protein-coding sequences were predicted based on results from GLIMMER (38). The coding sequences were further annotated using the stand-alone version of HMMER v3.1b2 and by downloading all HMM models for bacteria from eggNOG v4.5.0. Additional analysis was carried out using the UniProt database, the RAST database, and Cluster of Orthologous Group of Proteins (39). rRNA and tRNA genes were predicted with RNAmmer-1.2 and tRNA scan-SE (40). AntiSMASH was used to predict the gene clusters for the production of secondary metabolites, which was followed by manual correction (41).

### Construction of *S. xanthophaeus* no2 mutants.

To delete the gene *sxrX* from the rishirilide BGC in *S. xanthophaeus* no2, the plasmid pKGLP2-sxrX was constructed as described previously (23). First, a 5,282-bp PCR product containing the gene *sxrX* was ligated into pBluescript SK(−) to yield pBSK-sxrX. An *aac*(3)*IV*-*lox*p cassette amplified from pLERECJ was used to replace *sxrX* in pBSK-sxrX by λ Red recombination, yielding BW25113::pBSK-sxrX. Then, the fragment containing the gene *aac*(3)*IV* with homologous arms was amplified using primers listed in Table S4. The resulting fragment was cloned into pKGLP2-gusA to generate plasmid pKGLP2-sxrX. The plasmid was introduced into *S. xanthophaeus* no2 by intergeneric conjugation. The correct *S. xanthophaeus* no2 Δ*sxrX* exconjugant was selected as an apramycin-resistant and hygromycin-sensitive mutant and verified by PCR using the primers listed in Table S4.

To confirm the function of *sxrX*, the integrative plasmid pSET152-sxrX was established. The gene *sxrX* (798 bp) and its 350-bp upstream region were amplified from *S. xanthophaeus* no2 using the primers listed in Table S4. The *sxrX* gene was cloned into integration recombinant plasmid pSET152-hyg to construct plasmid pSET152-SxrX by Gibson assembly, in which the *sxrX* gene was under the control of a native promoter, and the construct was subsequently transferred into *S. xanthophaeus* no2 Δ*sxrX* by conjugation. The correct colonies were screened based on their apramycin resistance. In addition, the gene *sxrX* was cloned into a replicative recombinant plasmid, pUWL-H, to construct plasmid pUWL-H-sxrX, in which the *sxrX* gene was under the control of an ermEP* promoter, and then it was transferred into the *S. xanthophaeus* no2 Δ*sxrX* mutant strain by conjugation, yielding the recombinant strain *S. xanthophaeus* no2 Δ*sxrX*::pUWL-H-sxrX. The exconjugants were screened on the basis of phenotypes with hygromycin resistance and then confirmed by PCR.

To test the function of PPTase-encoding genes from *S. albus* J1074, the plasmids pSET152-SFP-AGI86981 and pSET152-SFP-AGI92022 were constructed. They were subsequently conjugated into the mutant *S. xanthophaeus* no2 Δ*sxrX* to test the PPTase protein function.

### Expression and purification of SxrX, ACPs, and PCPs.

The *sxrX* gene was amplified by PCR using the primers listed in Table S4 and the genome of *S. xanthophaeus* no2 as a template. PCR products were ligated into pET28a(+), GST, and SUMO vectors to generate plasmids pET28-N-sxrX, pET28-C-sxrX, GST-sxrX, and SUMO-sxrX. Using the *sxrX* gene sequence from *S. xanthophaeus* no2 for protein expression failed, and so an optimized *sxrX* gene sequence was synthesized and inserted into pET28a(+) vector; later, this insert was assembled into the pET21b(+) vector to generate plasmid pET21b-sxrX(opt). E. coli BL21(DE3) was used as the host for protein expression. The *sxrK1* genes were amplified and cloned by a similar method using the primers listed in Table S4 to generate plasmid pET28a-SxrK1, and further to construct the stain BL21(DE3)/pET28a-SxrK1 for protein overexpression. For protein purification, 20-mL overnight cultures of E. coli BL21(DE3)/pET21b-sxrX (opt) and BL21(DE3)/pET28a-SxrK1 were individually used to inoculate 2 liters of LB medium with antibiotics. Cells were grown at 37°C to an optical density at 600 nm of 0.6. After induction with 0.1 mM isopropyl-β-d-thiogalactopyranoside, cells were grown at 20°C for 16 h. Cells were harvested by centrifugation at 4°C (5,000 rpm, 10 min), resuspended in 30 mL of buffer A (50 mM Tris-HCl [pH 8.0], 0.5 M NaCl, 5 mM imidazole), and lysed by sonication on ice. Cell debris were removed by centrifugation at 12,000 rpm for 30 min at 4°C. Then, the supernatant was purified in a two-step procedure commencing with Ni-nitrilotriacetic acid (NTA) affinity chromatography using a 2 mL Ni-NTA column preequilibrated in buffer A. After washing with 5 column volumes (CV) of buffer A, bound protein was eluted with 1.5 CV of buffer B (buffer A plus 250 mM imidazole). Fractions were collected and analyzed by SDS-PAGE, and fractions containing target protein were concentrated by ultrafiltration before being further purified using gel filtration (Sephadex G-25 column, 200 mm by 40 mm; gel filtration buffer of 50 mM Tris-HCl [pH 7.4], 150 mM NaCl). After elution, those pure protein fractions were combined and concentrated using an Amicon Ultra centrifugal filter, and the buffer was then exchanged to 20 mM Tris-HCl (pH 8.0), 100 mM NaCl with 15% glycerol. Subsequently, the protein solution was aliquoted and flash-cooled in liquid nitrogen before being stored at −80°C.

### SxrX phosphopanteinylation assay.

A typical reaction mixture of 100 μL containing 100 mM Tris-HCl (pH 7.5), 1.25 mM MgCl_2_, 2.5 mM Tris(carboxyethyl)phosphine hydrochloride (TCEP), 200 μM ACP or PCP, 20 μM PPTase SxrX, and 2 mM CoA was incubated at 25°C for 30 min. The reactions were quenched by freezing reaction mixtures with liquid nitrogen. The conversion of *holo*-ACPs was detected based on the peak abundance levels of the *apo*-ACPs and *holo*-ACPs. The ACPs produced from E. coli or from the phosphopantetheinylation reaction mixture were analyzed by MALDI-TOF-MS. The matrix solvent used for mass analysis was 4-chloro-α-cyanocinnamic acid (α-CHCA; 10 mg/mL) in 50% acetonitrile (supplemented with 0.1% trifluoroacetic acid). The MALDI analysis was performed using the analysis method LP-imaging-2-20 kDa.

### RNA extraction and RT-PCR analysis.

*S. xanthophaeus* no2 and mutant *S. xanthophaeus* no2 Δ*sxrX* were cultivated in liquid GYM medium for 3 days for RNA extraction. Total RNA was extracted by using a bacterial RNA extraction kit (Vazyme Biotech Co., Ltd.), and RNA reverse transcription for cDNA was performed using the PrimeScript RT reagent kit with gDNA Eraser (Dalian TaKaRa Inc., China). The transcription of the regulatory gene *gyrB* was selected as a control. PCR primers are listed in Table S4. PCR conditions were as follows: 5 min at 98°C for one cycle, followed by 10 s at 98°C, 15 s at 58°C, and 2 min at 72°C for 40 cycles, and finally one cycle for 10 min at 72°C.

### Modeling of PPTase SxrX and ACP SxrK1.

The protein structures of SxrX and SxrK1 were generated using the server-based program AlphaFold v2.0 Google Colab notebook in the alignment mode (42). The electrostatic surface models were prepared using the PyMol molecular graphic system (Schrodinger, LLC). The docking simulation of the SxrX-SxrK1 complex structures used AlphaFold v2.0, which can be used for searching all possible binding modes in the translational and rotational space between two proteins, and we evaluated each pose using an energy-based scoring function. All parameters remained at their default values.

### Site-directed mutagenesis in the SxrX protein.

To investigate the protein-protein interaction region in SxrX, the possible active residues were selected for verification through a site-specific mutation strategy. In *S. xanthophaeus* no2 Δ*sxrX*, 10 amino acids are predicted to be involved in the interaction between PPTase and ACP proteins, and point mutations were constructed that included PPTase mutations R36A, R37A, R39A, T40A, R136A, D130A, D132A, R127A, W160A, and W160F, which were introduced to disrupt the possible hydrophilic interaction between those two proteins.

The site-directed mutagenesis of SxrX was conducted using the overlap-extension PCR method (43). According to the manual’s protocol with primers listed in Table S4, a series of plasmids based on vector pSET152-sxrX was constructed. These residues were mutated to alanine or phenylalanine, respectively, yielding mutated plasmids pSET152-sxrX-R36A, pSET152-sxrX-R37A, pSET152-sxrX-R39A, pSET152-sxrX-T40A, pSET152-sxrX-R136A, pSET152-sxrX-D130A, pSET152-sxrX-D132A, pSET152-sxrX-R127A, pSET152-sxrX-W160A, and pSET152-sxrX-W160F. Subsequently, these resulting plasmid mutation sites were confirmed by DNA sequencing. After that, the recombinant plasmids were individually transformed into E. coli ET12567(pUZ8002) and then transferred into the Δ*sxrX* mutant strain by conjugation. The exconjugants were selected on the basis of phenotypes showing hygromycin resistance and then confirmed by PCR.

### Molecular dynamics simulation.

MD simulations of the SxrX-SxrK1 complex and corresponding mutation systems were performed using the Amber18 software package with the ff14SB force field (44). transferable interatomic potential with three points model (TIP3P) water molecules were utilized to solvate the complex, extending at least 10 Å from the protein, while counterions were added to neutralize the system. In the latter, all bond lengths involving hydrogen atoms were restricted with the SHAKE algorithm (45). A cutoff of 8 Å was used for van der Waals forces and short-range electrostatic interactions. To remove the bad contacts, the system was subjected to energy minimization. First, the water molecules and ions were refined through 2,500 steps of steepest descent followed by 2,500 steps of conjugate gradient, keeping the protein and ligands fixed. Second, the whole system was relaxed by 10,000 cycles of minimization procedure with 5,000 cycles of steepest descent and 5,000 cycles of conjugate gradient minimization. After that, the system was heated from 0 to 310 K in a 500-ps position restraint simulation. Next, 2-ns MD simulations without any restraints were sequentially performed to equilibrate the complex. Finally, a 100-ns trajectory was computed at 310 K under constant pressure. Hydrophobic interaction was defined as a distance between the hydrophobic cores of two residues that was <6.5 Å. Binding free energies in all systems were calculated using the GB model in MMPBSA.py (46) embedded in the AMBER package. Results of the evolutionary conservation were analyzed using the ConSurf server (47). Structural figures were drawn using the PyMOL software (http://www.pymol.org).

### Data availability.

The genome sequence of *S. xanthophaeus* no2 has been deposited in the GenBank database with BioProject number PRJNA735883.
