# Supplementary material for: Enhanced Rishirilide Biosynthesis by a Rare In-Cluster Phosphopantetheinyl Transferase in Streptomyces xanthophaeus
Source: Microbiol Spectr. 2022 Nov 3;10(6):e03247-22. doi: 10.1128/spectrum.03247-22 (PMC9769936; doi:10.1128/spectrum.03247-22)
Supplement: Supplemental file 1 — Supplemental results and methods, Tables S1 to S11, and Fig. S1 to S37. Download spectrum.03247-22-s0001.pdf, PDF file, 4.6 MB [file spectrum.03247-22-s0001.pdf]

Supplemental Material

Enhanced rishirilide biosynthesis by a rare in-cluster phosphopantetheinyl transferase in *Streptomyces xanthophaeus*

Songya Zhang,[a] Shuai Fan,[b] Jing Zhu,[a] Liying Zhou,[c] Xiaohui Yan,[d] Zhaoyong Yang,[b] Tong Si,[a]# Tao Liu[c]#

[a]CAS Key Laboratory of Quantitative Engineering Biology, Shenzhen Institute of Synthetic Biology, Shenzhen Institute of Advanced Technology, Chinese Academy of Sciences, Shenzhen, China
[b]The Institute of Medicinal Biotechnology, Chinese Academy of Medical Sciences and Peking Union Medical College, Beijing, China.
[c]Department of Natural Products Chemistry, School of Pharmacy, China Medical University, Shenyang, China
[d]State Key Laboratory of Component-based Chinese Medicine, Tianjin University of Traditional Chinese Medicine, Tianjin, China

#Address correspondence to Tong Si, tong.si@siat.ac.cn, or Tao Liu, liutao@cmu.edu.cn.

Songya Zhang and Shuai Fan contributed equally to this work. Author order was determined on the basis of seniority.

Supplemental Results

General Characterization of *S. xanthophaeus* no2 and *S. xanthophaeus* no2 *ΔsxrX*

The strain *S. xanthophaeus* no2 produces whitish, medium-sized, fuzzy colonies when cultured on MS agar plates. The spores had a smooth surface, were oblong in shape, and were arranged in chains when observed by Scanning Electron Microscopic (Fig. S36). The morphology of *S. xanthophaeus* no2ΔsxrX on the MS agar plate shows its mycelium is white and hard, with depressions all around, and most of whose surface exist shallow brown spores, and less no spores. The morphology on the scanning electron microscope indicates that its hyphae are slender, and has a smooth surface without obvious shrinkage and deformation.

New peaks discovered from the mutant *ΔsxrX*

Acetylleucylleucine (N-acetyl-L-leucyl-L-leucine) [1, 2] and Propionylleucylleucine (N-propionyl-L-leucyl-L-leucine) [2] were isolated from strain *S. xanthophaeus no2ΔsxrX* through large-scale fermentation in TSB medium, and their chemical structures were determined based on the MS and NMR data (Fig. S18-S26).

Acetylleucylleucine (**I**): white needle crystal, $C_{14}H_{26}N_2O_4$; Negative HR-ESIMS *m/z*: 285.1824 $[M\text{-}H]^-$, calculated as 285.1820 for $C_{14}H_{25}N_2O_4$; ^{1}H-NMR and ^{13}C-NMR data, see Table S7.

Propionylleucylleucine (**II**): $C_{15}H_{28}N_2O_4$; Negative HR-ESIMS *m/z*: 299.1980 $[M\text{-}H]^-$, calculated as 299.1976 for $C_{15}H_{27}N_2O_4$.

The rishirilide derivatives discovered from *Streptomyces xanthophaeus*

Rishirilide A: yellow amorphous powder, $C_{21}H_{24}O_7$; $[\alpha]^{20}_D$ = - 459.6° (*c* 0.164, MeOH); Negative HR-ESIMS *m/z*: 387.1455 $[M\text{-}H]^-$, calculated as 387.1449 for $C_{21}H_{23}O_7$; ^{1}H-NMR, ^{13}C-NMR, HMBC and NOESY data, see Table S8.

Rishirilide B: dark brown solid, $C_{21}H_{24}O_6$; Negative HR-ESIMS *m/z*: 371.1504 $[M\text{-}H]^-$, calculated as 371.1500 for $C_{21}H_{23}O_6$; ^{1}H-NMR and ^{13}C-NMR data, see Table S9.

Rishirilide C: brown solid, $C_{21}H_{24}O_7$; Negative HR-ESIMS *m/z*: 387.1453 $[M\text{-}H]^-$, calculated as 387.1449 for $C_{21}H_{23}O_{67}$; ^{1}H-NMR and ^{13}C-NMR data, see Table S10.

Lupinacidin A: brick red amorphous powder, $C_{20}H_{20}O_5$; Negative HR-ESIMS *m/z*: 339.1241 $[M\text{-}H]^-$, calculated as 339.1238 for $C_{20}H_{19}O_5$; ^{1}H-NMR and ^{13}C-NMR data, see Table S11.

Supplemental Methods

Isolation of strain

S. xanthophaeus no2 was isolated from the soil collected from Jiangxi Province, China (28° 4′ 5″N, 115° 3′ 34″E). The strain was deposited at the Department of Natural Products Chemistry, School of Pharmacy, China Medical University with the voucher strain number

GA1-1-4. For strain purification, the rhizosphere soil was scraped into a sterilized plate and heated at 60~70 °C for 4 h to remove non-sporulating bacteria. Then, 0.5 g of soil was weighed and suspended in 5.0 mL of sterile water. After a series of dilution (10^{-1}, 10^{-2}, 10^{-3}, 10^{-4}, 10^{-5}, 10^{-6}), a 50 μL diluent was spread on M2 agar plates (malt extract 10.0 g, glucose 4.0 g, yeast extract 4.0 g, $CaCO_3$ 2.0 g, agar 18.0 g, demineralized water 1 L). After one week of cultivation at 28 °C, visible colonies of actinomycetes were picked and streaked on new M2 agar plates. This purification process was repeated one time.

Morphological characterization

The primers for amplification of the gene encoding 16S rRNA are pA and pH [3]. The sequencing result revealed a high sequence similarity (100%, 1465/1466) to *S. netropsis* NBRC 12,893 as the closest homologous strain (Fig. S37). The isolate, *S. xanthophaeus* no2, was identified using various cultural characteristics on different medium. Genus-level identification of the isolate was carried out based on their morphology following the Bergey's Manual of Determinative Bacteriology. The arrangement of the spores in the mycelium was observed by the cover slip method under light microscope and by scanning electron microscope. Morphological characteristics of the mycelia surface were examined using a JSM-5410LV scanning electron microscopy (JEOL, Japan). The sample was prepared as protocol described previously. The spores of *S. xanthophaeus* no2 were collected, washed with phosphate-buffered saline (PBS), and fixed with 3% glutaraldehyde at 4 °C overnight. The fixed mycelium was washed with PBS three times (15 min each) and then fixed with 1% OsO4 for 1 h. Subsequently, an ethanol concentration gradient (v/v) of 30%, 50%, 90%, and 100% was used to dehydrate the fixed mycelia sequentially.

Supplemental Tables

Table S1. General features of the genome of *S. xanthophaeus* no2.

Features	Chromosome Characteristics
Genome topology	Linear

Chromosome size (bp)	8,324,019
Scaffolds	3
G+C content (%)	71.32
Protein-coding genes	7115
Gene density (per kb)	0.85
Average ORF size (bp)	1018.05
rRNA (16S-23S-5S) operons	21
sRNA	1
Number of tRNA	78
Secondary metabolites gene cluster	40
GenBank accession number	PRJNA735883

Table S2. The deduced function of ORFs in the *sxr* biosynthetic gene cluster.

no	ORF	Size (Amino Acid)	Proposed function	Protein homologue, origin and /similarity	Identity (%)
**1**	*Orf(-3)*	425	1-deoxy-D-xylulose-5-phosphate reductoisomerase	WP_053704903.1 [Streptomyces sp. WM6368]	98
**2**	*Orf(-2)*	422	hypothetical protein	WP_030718653.1 [Streptomyces sp. NRRL S-237]	92
**3**	*Orf(-1)*	644	acyl-CoA-dehydrogenase	CAA16488.1	35
**4**	SxrC1	317	aromatase	RslC1(AHL46707.1); Streptomyces bottropensis	72
**5**	SxrK1	89	acyl carrier protein	RslK1(AHL46708.1); Streptomyces bottropensis	80
**6**	SxrK2	411	Minimal PKS chain-length factor (CLF/KS β)	RslK2(AHL46709.1)	72
**7**	SxrK3	422	Minimal PKS ketosynthetase (KS/KS α)	RslK3(AHL46710.1)	89
**8**	SxrA	371	acyl transferase	RslA(AHL46711.1)	62
**9**	SxrK4	374	3-oxoacyl-ACP synthase III	RslK4(AHL46712.1)	75
**10**	SxrX	225	4'-phosphopantetheinyl transferase	AsuC1 (ADI58627.1)	44
**11**	SxrT1	322	ABC-transporter (substrate binding)	RslT1(AHL46713.1)	71
**12**	SxrT2	255	ABC-transporter (ATP-binding)	RslT2(AHL46714.1)	73

**13**	SxrT3	306	ABC-transporter transmembrane	RslT3(AHL46715.1)	84
**14**	SxrO1	354	luciferase-like monooxygenase	RslO1(AHL46716.1)	90
**15**	SxrO2	167	flavin reductase	RslO2(AHL46717.1)	67
**16**	SxrP	390	Putative aminoglycoside phosphotransferase	RslP(AHL46718.1)	69
**17**	SxrR1	267	SARP family transcriptional regulator	RslR1(AHL46719.1)	68
**18**	SxrC2	301	Second ring cyclase	RslC2(AHL46720.1)	74
**19**	SxrO3	239	3-oxoacyl-ACP reductase	RslO3(AHL46721.1)	73
**20**	SxrO4	99	monooxygenase	RslO4(AHL46722.1)	86
**21**	SxrO5	364	NADH-flavin oxidoreductase	RslO5(AHL46723.1)	75
**22**	SxrC3	155	cyclase/dehydrogenase	RslC3(AHL46724.1)	75
**23**	SxrR2	276	SARP regulatory protein	RslR2(AHL46725.1)	69
**24**	SxrR3	1125	SARP regulatory protein	RslR3(AHL46726.1)	67
**25**	SxrO6	340	luciferase-like monooxygenase	RslO6(AHL46727.1)	82
**26**	SxrR4	152	MarR family transcriptional regulator	RslR4(AHL46728.1)	80
**27**	SxrT4	518	drug resistance transporter	RslT4(AHL46729.1)	81
**28**	SxrO7	306	putative NADPH quinone reductase	RslO7(AHL46730.1)	77
**29**	SxrO8	325	NADPH-quinone oxidoreductase	RslO8(AHL46731.1)	71
**30**	SxrO9	544	FAD-binding monooxygenase	RslO9(AHL46732.1)	77
**31**	SxrO10	270	ketoreductase	RslO10(AHL46733.1)	86
**32**	SxrH	408	amidohydrolase	RslH(AHL46734.1)	68
**33**	*Orf(+1)*	75	hypothetical protein	No hit with significant similarity	-
**34**	*Orf(+2)*	481	aldehyde dehydrogenase	WP_161289953.1 [Streptomyces sp. SID1046]	99
**35**	*Orf(+3)*	526	PucR family transcriptional regulator	WP_150259773.1 [*Streptomyces venezuelae*]	97

Table S3. Bioinformatics of possible genes for the biosynthesis of Acetylleucylleucine and Propionylleucylleucine.

ORF (start-end) in *S. xanthophaeus* no2	*S. albulus* CCRC 11814 (Identity %)	*X. bovienii* SS-2004	Function

7481980 -7480838	WP_016577536.1 (61%)	*leupA*	acyl-protein synthetase LuxE
7480814-7478316	WP_020930524.1 (61%)	*leupB*	AMP-binding protein
7485778-7484504	WP_037635192.1 (61%)	*leupC*	AMP-binding protein
7483140-7482019	WP_020930523.1 (53%)	*leupD*	GNAT family N-acetyltransferase

Note: Genes are reported with UniProtKB entry or NCBI accession number. Amino acid identity was calculated by comparison with genes in *S. xanthophaeus* no2.

Table S4. Primers used in this study.

Primer	Sequence (5'-3')	Purpose
pBSK-kan-GA-F	ACGCTCAGTGGAACGAAAACTCACGTTAAGGGATTTTGGTCATGAAC	For replacing the ampicillin antibiotic resistance marker of pBluescript II SK(+) to kanamycin
pBSK-kan-GA-R	GCACTTTTCGGGGAAATGTCGCGGAACCCCTATTTGTTTATTTTTCT	
pBSK-b-F	ACATTTCCCCGAAAAGTGCCAC	
pBSK-b-R	TTTTCGTTCCACTGAGCGTCAG	
pBSK-BGCsfp-F	TGACGGCGGCACACCGGG	For deletion of gene *sxrX*
pBSK-BGCsfp-R	GTCCTCGCTGCGCCCCTG	
Apra-sfp-F	ATCCCGCGCCGGGTGACCCTGTGGCAGGGGCGCAGCGAGGACGATATCTCTAGATACCG	
Apra-sfp-R	CCGCAGCTTCCCGAAGCGCCGGGCCCGGTGTGCCGCCGTCAAACAAAAGCTGGAGCTC	
pUWL-ZSY2ctg7-SFP-F	CCCAAGCTTGTTCACCTGGAGCTGCGCC	For construction of plasmid pUWL-sxrX
pUWL-ZSY2ctg7-SFP-R	GGACTAGTCCGTCACCAGCGGCACAC	
SFPzsy2-GApSET152-F	TGGGCTGCAGGTCGACTCTAGATACACCGTCGCGCGGATCA	For construction of plasmid pSET152-sxrX
SFPzsy2-GApSET152-R	TGACATGATTACGAATTCGATATCGTTCCCTGACCGCAGCTTCCC	
SFPzsy-P500-pSET-F	TGGGCTGCAGGTCGACTCTAGACTGGTCAGTCTGGCGACCGT	
SFP-AGI86981GA-F	TGGGCTGCAGGTCGACTCTAGATCAACGGCATCGTGCACAGC	For construction of plasmid pSET152-SFP-AGI86981
SFP-AGI86981GA-R	TGACATGATTACGAATTCGATATCTCATGGACAGGGCAGGACGG	
SFP-AGI92022GA-F	TGGGCTGCAGGTCGACTCTAGATCCTCGGCTACGACTACACCTGG	For construction of plasmid pSET152-SFP-AGI92022
SFP-AGI92022GA-R	TGACATGATTACGAATTCGATATCTGTACTGCCTCACCGACCGC	
NdeI-SFP-F	TAACATATGATCCCGCGCCGGGTGA	For construction of plasmid pET28a-N-sxrX
HindIII-SFP-R	TAACCGCAGCTTCCCGAAGCGC	
pET21b-G-F	ATGTATATCTCCTTCTTAAAGTTAAACAAAATTATTTCTAGAGG	For construction of plasmid pET21b-sxrX
pET21b-G-R	CTCGAGCACCACCACCAC	
SFP-G-F	TGGTGGTGGTGGTGCTCGAGCCAGCGGCACACCGGGCC	
SFP-G-R	TTTAAGAAGGAGATATACATATGGTGATCCCGCGCCGG	
ctg7_22SFP-SUMO-F	GCTCACAGAGAACAGATTGGTGGATCCATGGTGATCCCGCGCCGG	For construction of plasmid SUMO-sxrX

ctg7_22SFP-SUMO-R	GTGGTGGTGGTGGTGGTGCTCGAGTCACCAGCGGCACACCGG	
SFP-zsy2-GST(BamHI)-GA-F	TGTTCCAGGGGCCCCTGGGATCCATGATCCCGCGCCGGGTG	For construction of plasmid GST-sxrX
SFP-zsy2-GST(XhoI)-GA-R	CAGTCACGATGCGGCCGCTCGATCACCAGCGGCACACCGG	
optSFP-zsy2-pET21b-GA-F	GTTTAACTTTAAGAAGGAGATATACATATGATTCCGCGCCGCG	For construction of plasmid pET21b-sxrX (opt)
optSFP-zsy2-pET21bSUMO-GA-F	CTTTAAGAAGGAGATATACATATGTCGGACTCAGAAGTCAATC	
PCP1-sas17pET28a(NdeI)-F	CTGGTGCCGCGCGGCAGCCATATGGCGCCCGACTTCGGCGCCG	For construction of plasmid pET28a-PCP1sas17
PCP1-sas17pET28a(HindIII)-R	GGTGCTCGAGTGCGGCCGCAAGCTTCTACCGCGCCTCGGGCAGG	
ACPZSY2(HindIII)-GA-R	GGTGCTCGAGTGCGGCCGCAAGCTTTCAGCGGGCGGAGAGC	For construction of plasmid pET28a-SxrK1
ACPZSY2(NdeI)-GA-F	CTGGTGCCGCGCGGCAGCCATATGAGCGCGACCGAGACCG	
rbs-SFP-zsy2(XbaI)	CTAGTCTAGAAACGGTGCCTGGTGTTCGGT	For construction of plasmid pSET152-sxrX
rbs-SFP-zsy2(EcoRV)	TGCGATATCTTCCCTGACCGCAGCTTCCC	
RT_ctg007-1006-F	TCCGGACCGCACTGGATG	For RT-PCR test of gene ctg007-1006
RT_ctg007-1006-R	GGAACAGGCTGTACACCTGCCA	
RT_ctg016-1818-F	ATGTGCCAGGACTTCACGCC	For RT-PCR test of gene ctg016-1818
RT_ctg016-1818-R	ATCGACGTAGCGGAGATCGAGC	
RT_ctg022-2273-F	CGTCGATGTGCAAGAGGTACTGC	For RT-PCR test of gene ctg022-2273
RT_ctg022-2273-R	GAGGTCCTCCACCTGCCACAG	
RT_ctg032-2936-F	GTCGGAGTGACGTGCTGTCG	For RT-PCR test of gene ctg032-2936
RT_ctg032-2936-R	AGTAGGTCGGCACGGAAGGT	
RT_ctg040-3497-F	TCTCCCTCTCCCACAGCGG	For RT-PCR test of gene ctg040-3497
RT_ctg040-3497-R	CGTGACGGGCTGGATCCTC	
RT_ctg046-3754-F	CACACGCCCTGTACGTCTCC	For RT-PCR test of gene ctg046-3754
RT_ctg046-3754-R	GGATGCCGAGGATCGGGAT	
RT_ctg073-4969-F	AGGACCGCGGCTCCTATCT	For RT-PCR test of gene ctg073-4969
RT_ctg073-4969-R	CTCGTCGGGGTGGAAGAAGT	
RT_gyrB-F	AGAAGGTGACGAACTGCTCTCGA	For RT-PCR test of gene *gyrB*
RT_gyrB-R	CAGGTTGATCAGCTGGACGCC	
SSM-SFP-zsy2-R1	TCCGCCCGAGGGCAGA	For construction of plasmid pSET152-sxrX
SSM-SFP-zsy2-F2	CTCTGCCCTCGGGCGGACCACGCCCATCATGACCTTG	
36R-SFP-zsy2-F1	CGGCTGCGCACTCCCCT	For site-directed single mutation at position 36R
36R-SFP-zsy2-R2	AGGGGAGTGCGCAGCCGCGCTAACCGCTCCAGCTCGT	
160A-SFP-zsy2-F1	ACCCGCAAGGAAGCGG	For site-directed single mutation at position 160A and 160F
160A-SFP-zsy2-R2	CCGCTTCCTTGCGGGTCGCGCACCGGAAGGCCAGCG	

160F-SFP-zsy2-R2	CCGCTTCCTTGCGGGTGAAGCACCGGAAGGCCAGCG	
136R-SFP-zsy2-F1	GCGGCGTTCTCCGCC	For site-directed single mutation at position 136R
136R-SFP-zsy2-R2	GGCGGAGAACGCCGCCGCGGCCACGGCGTCGACG	
37R-SFP-zsy2-F1	CTGCGCACTCCCCTCGC	For site-directed single mutation at position 37R
37R-SFP-zsy2-R2	GCGAGGGGAGTGCGCAGCGCGCGTAACCGCTCCAGCT	
39R-SFP-zsy2-F1	ACTCCCCTCGCCGCC	For site-directed single mutation at position 39R
39R-SFP-zsy2-R2	GGCGGCGAGGGGAGTCGCCAGCCGGCGTAACCGC	
40R-SFP-zsy2-F1	CCCCTCGCCGCCCGCC	For site-directed single mutation at position 40R
40R-SFP-zsy2-R2	GCGGGCGGCGAGGGGCGCGCGCAGCCGGCGTAAC	
130R-SFP-zsy2-F1	GTCGACGCCGTGGCCC	For site-directed single mutation at position 130R
130R-SFP-zsy2-R2	GGGCCACGGCGTCGACCGCCAGCGGCCGCTCCCGT	
132R-SFP-zsy2-F1	GCCGTGGCCCGTGCG	For site-directed single mutation at position 132R
132R-SFP-zsy2-R2	GCCGCACGGGCCACGGCCGCGACGTCCAGCGGCCG	
127R-SFP-zsy2-F1	CCGCTGGACGTCGACGCC	For site-directed single mutation at position 127R
127R-SFP-zsy2-R2	GGCGTCGACGTCCAGCGGCGCCTCCCGTTCGAGGTCCACC	
pA	AGAGTTTGATCCTGGCTCAG	Primer for the 16s RNA analysis
pH	AAGGAGGTGATCCAGCCGCA	
pUWL-H-ctg7_22sfp-F	CCCAAGCTTGTTCACCTGGAGCTGCGCC	PCR verification for deletion of gene *sxrX*
pUWL-H-ctg7_22sfp-R	GGACTAGTCCGTCACCAGCGGCACAC	

Table S5. Plasmid used in this study.

**Name**	**Description**	**Reference**
pET28a(+)	Protein expression vector	Invitrogen
pBluescript SK(-)	Vector for cloning	Lab stock
pET28-N-sxrX	Vector for protein expression of SxrX	This study
pET28-C-sxrX	Vector for protein expression of SxrX	This study
SUMO-sxrX	Vector for protein expression of SxrX	This study
GST-sxrX	Vector for protein expression of SxrX	This study
pET21b-sxrX (opt)	Vector for protein expression of SxrX	This study
pUWL-H-sxrX	For overexpression of SxrX in *S. xanthophaeus* no2Δ*sxrX*	This study
pSET152-hyg	ΦC31 attachment site, integrative, Hyg^R	Lab stock
pSET152-sxrX	For overexpression of SxrX	This study
pBSK-kan	Vector for replacing the antibiotic resistance marker of pBluescript II SK(+) to kanamycin	This study
pLERECJ	Vector carrying apramycin resistance cassette with loxP-sites for gene replacement	Prof. Luzhetskyy, Saarland University

pBSK-sxrX	Vector for constructing plasmid pKGLP2-sxrX	This study
pKGLP2-gusA	pKCLP2 derivative with *gusA* gene	Lab stock
pKGLP2-sxrX	Plasmid for deletion of gene *sxrX*	This study
pET28a-SxrK1	Plasmid for ACP protein SxrK1 expression	This study
pET28a- PCP_{ws9326}	Plasmid for PCP_{ws9326} protein expression	This study
pSET152-sxrX	For gene *sxrX* complementation	This study
pSET152-sxrX-R36A	For complementation of SxrX with point-mutation R36A	This study
pSET152-sxrX-R37A	For complementation of SxrX with point-mutation R37A	This study
pSET152-sxrX-R39A	For complementation of SxrX with point-mutation R39A	This study
pSET152-sxrX-T40A	For complementation of SxrX with point-mutation T40A	This study
pSET152-sxrX-R136A	For complementation of SxrX with point-mutation R136A	This study
pSET152-sxrX-D130A	For complementation of SxrX with point-mutation D130A	This study
pSET152-sxrX-D132A	For complementation of SxrX with point-mutation D132A	This study
pSET152-sxrX-R127A	For complementation of SxrX with point-mutation R127A	This study
pSET152-sxrX-W160A	For complementation of SxrX with point-mutation W160A	This study
pSET152-sxrX-W160F	For complementation of SxrX with point-mutation W160F	This study
pSET152-SFP-AGI86981	For gene AGI86981 complementation in *S. xanthophaeus* no2Δ*sxrX*	This study
pSET152-SFP-AGI92022	For gene AGI92022 complementation in *S. xanthophaeus* no2Δ*sxrX*	This study

Table S6. Strains used in this study.

**Name**	**Relevant characteristics**	**Reference**
E. coli DH5α	General cloning host	Lab stock
E. coli BL21(DE3)	T7 expression host	Lab stock
E. coli ET12567(pUZ8002)	Strain for conjugation	Lab stock
E. coli ET12567(pR9406)	Strain for conjugation	Lab stock
Streptomyces xanthophaeus no2	Wildtype strain of rishirilide producer	Lab stock
S. xanthophaeus no2*ΔsxrX*	Mutant with gene *sxrX* deletion in *S. xanthophaeus* no2	This study
S. xanthophaeus no2Δ*sxrX*::pSET152-SxrX	Mutant *S. xanthophaeus no2ΔsxrX* carrying plasmid pSET152-SxrX	This study
S. xanthophaeus no2Δ*sxrX*::pUWL-H-sxrX	Mutant *S. xanthophaeus no2ΔsxrX* carrying plasmid pUWL-H-sxrX	This study
Streptomyces albus J1074	For gene amplification	Lab stock
E. coli BL21(DE3)/pET28-N-sxrX	For SxrX protein expression	This study
E. coli BL21(DE3)/pET28-C-sxrX	For SxrX protein expression	This study
E. coli BL21(DE3)/SUMO-sxrX	For SxrX protein expression	This study

S. xanthophaeus no2Δ*sxrX*::pSET152-SFP-AGI86981	The mutant *S. xanthophaeus* no2Δ*sxrX* with gene *AGI86981*	This study
S. xanthophaeus no2Δ*sxrX*::pSET152-SFP-AGI92022	the mutant *S. xanthophaeus* no2Δ*sxrX* with gene *AGI92022*	This study
E. coli BW25113::pKD46	For Red/ET homologous recombination	[4]
E. coli BW25113::pBSK-*sxrX*	For deletion of gene *sxrX*	This study
BL21(DE3)/pET28a-PCP_{ws9326}	For PCP_{ws9326} protein expression	This study
BL21(DE3)/pET28a-SxrK1	For SxrK1 protein expression	This study
E.coli BL21(DE3)/pET21b-sxrX (opt)	For SxrX protein expression	This study
S. xanthophaeus no2Δ*sxrX*::pSET152-sxrX	For gene *sxrX* complementation	This study
S. xanthophaeus no2Δ*sxrX*::pSET152-sxrX-R36A	For complementation of SxrX with point-mutation R36A	This study
S. xanthophaeus no2Δ*sxrX*::pSET152-sxrX-R37A	For complementation of SxrX with point-mutation R37A	This study
S. xanthophaeus no2Δ*sxrX*::pSET152-sxrX-R39A	For complementation of SxrX with point-mutation R39A	This study
S. xanthophaeus no2Δ*sxrX*::pSET152-sxrX-T40A	For complementation of SxrX with point-mutation T40A	This study
S. xanthophaeus no2Δ*sxrX*::pSET152-sxrX-R136A	For complementation of SxrX with point-mutation R136A	This study
S. xanthophaeus no2Δ*sxrX*::pSET152-sxrX-D130A	For complementation of SxrX with point-mutation D130A	This study
S. xanthophaeus no2Δ*sxrX*::pSET152-sxrX-D132A	For complementation of SxrX with point-mutation D132A	This study
S. xanthophaeus no2Δ*sxrX*::pSET152-sxrX-R127A	For complementation of SxrX with point-mutation R127A	This study
S. xanthophaeus no2Δ*sxrX*::pSET152-sxrX-W160A	For complementation of SxrX with point-mutation W160A in *S. xanthophaeus* no2Δ*sxrX*	This study
S. xanthophaeus no2Δ*sxrX*::pSET152-sxrX-W160F	For complementation of SxrX with point-mutation W160F in *S. xanthophaeus* no2Δ*sxrX*	This study

Table S7. The NMR summary of compound acetyl-leucyl-leucine.

Position	δ_H (*J* in Hz) [a]	δ_C (ppm) [b]	DEPT 135	DEPT 90	HMBC
1		175.8			
2	4.43, m	52.0	CH	CH	1, 13, 14

3	/	/	/	/	/
4		175.0			
5	4.41, m	53.1	CH	CH	4, 7, 9, 10
6	/	/	/	/	/
7		173.2			
8	1.97, s	22.3	CH_3		7
9	1.58, m	41.6	CH_2		4, 5, 10, 11, 12
10	1.73, m	25.8	CH	CH	9
11	0.92, t (6.4)	22.1	CH_3		9, 10
12	0.92, t (6.4)	21.8	CH_3		9, 10
13	1.64, m	41.9	CH_2		1, 2, 14, 15, 16
14	1.72, m	25.9	CH	CH	2, 13, 15, 16
15	0.96, m	23.4	CH_3		13, 14, 16
16	0.96, m	23.4	CH_3		13, 14, 15

[a] 400 MHz, in methanol-d_4; [b] 100 MHz, in methanol-d_4.

Table S8. The NMR summary of rishirilide A.

Position	δ_H (J in Hz) [a]	δ_C (ppm) [b]	HMBC	NOESY
1		199.4		
2	2.78, 1H, q (7.4)	50.7	1, 3, 16, 17	11, 17
3		81.8		
4		82.1		
4a		85.4		
5		156.8		
6	7.00, 1H, d (7.2)	120.7	5, 7, 10, 10a	7
7	7.25, 1H, t (7.8)	131.1	5, 6, 8, 8a	6, 8
8	7.00, 1H, d (7.2)	123.8	6, 7, 8a, 9, 10, 10a	7, 9
8a		131.8		
9	7.59, 1H, s	139.6	1, 4a, 5, 8a, 9a, 10a	8
9a		131.9		
10	5.50, 1H, s	64.5	1, 4a, 5, 8a, 9a, 10a	
10a		123.2		
11	2.51, 1.61, 2H, m	31.0	3, 12, 13, 15	2, 14, 15
12	1.49-1.70, 2H, m	32.6	15	

13	1.39, 1H, m	30.0	11, 15	
14	0.89, 3H, d (6.6)	23.1	12, 13, 15	11
15	0.87, 3H, d (6.6)	22.7	12, 13, 14	11
16		177.5		
17	1.22, 3H, d (7.4)	12.6	1, 2, 3	2

[a] 400 MHz, in methanol-d_4; [b] 100 MHz, in methanol-d_4.

Table S9. The NMR summary of rishirilide B.

Position	δ_H (*J* in Hz) [a]	δ_C (ppm) [b]
1		200.5
2	3.08, 1H, q (6.7)	49.6
3		85.8
4		78.6
4a		140.1
5		154.7
6	6.89, 1H, d (7.4)	111.1
7	7.28, 1H, m	127.6
8	7.44, 1H, d (8.3)	121.5
8a		134.4
9	8.38, 1H, s	127.9
9a		130.7
10	8.41, 1H, s	121.7
10a		128.5
11	2.35, 1.65, 2H, m	37.1
12	1.45, 0.90, 2H, m	32.7
13	1.36, 1H, m	29.7
14	0.73, 3H, d (6.6)	23.1
15	0.85, 3H, d (6.6)	23.2
16		175.7
17	1.28, 3H, d (6.7)	11.0

[a] 600 MHz, in methanol-d_4; [b] 150 MHz, in methanol-d_4.

Table S10. The NMR summary of rishirilide C.

Position	δ_H (*J* in Hz) [a]	δ_C (ppm) [b]
1		206.0

2	3.10, 1H, q (6.4）	49.7
3		85.4
4		78.8
4a		139.9
5		154.7
6	6.97, 1H, d (7.4）	113.7
7	7.26, 1H, dd (7.4, 8.0)	126.8
8	7.80, 1H, d (8.0)	115.9
8a		126.7
9		162.5
9a		112.0
10	7.82, 1H, s	111.2
10a		129.1
11	2.31, 1.63, 2H, m	36.3
12	1.43 , 0.89, 2H, m	32.8
13	1.36, 1H, m	29.8
14	0.72, 3H, d (6.5)	23.1
15	0.83, 3H, d (6.5）	23.4
16		175.7
17	1.32, 3H, d (6.4）	10.4

[a] 600 MHz, in methanol-d_4; [b] 150 MHz, in methanol-d_4.

Table S11. The NMR summary of lupinacidin A.

Position	δ_H (J in Hz) [a]	δc (ppm) [b]
1		162.3
1-OH	14.22, 1H, s	
2		117.8
3		161.6
4		130.4
4a		127.5
5		161.6
5-OH	12.82, 1H, s	
6	7.32,1H, dd (1.1, 7.5)	124.0
7	7.75, 1H, m (7.5, 8.3)	136.6
8	7.68 , 1H, dd (1.1, 8.3)	118.0
8a		132.8

9		185.7
9a		109.1
10		190.2
10a		116.7
11	3.14, 2H, m	24.6
12	1.35, 2H, m	37.5
13	1.72, 1H, m	28.4
14	0.98, 3H, d (6.6)	22.5
15	0.98, 3H, d (6.6)	22.5
16	2.15, 3H, s	9.1

[a] 600 MHz, in DMSO-d_6; [b] 150 MHz, in DMSO-d_6.

Supplemental Figures

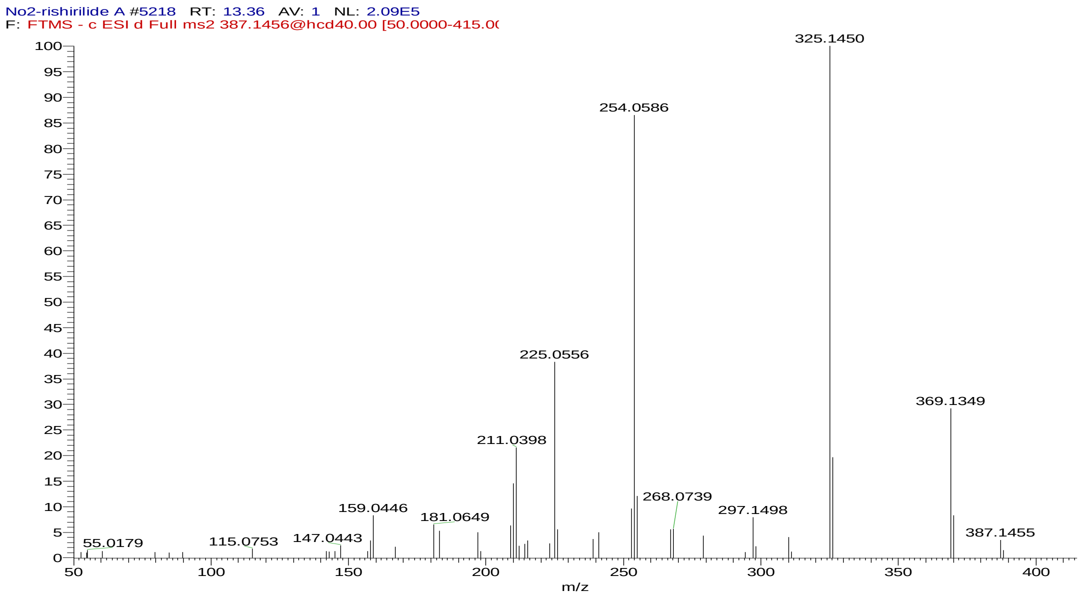

Figure S1. The HR ESIMS spectrum of rishirilide A.

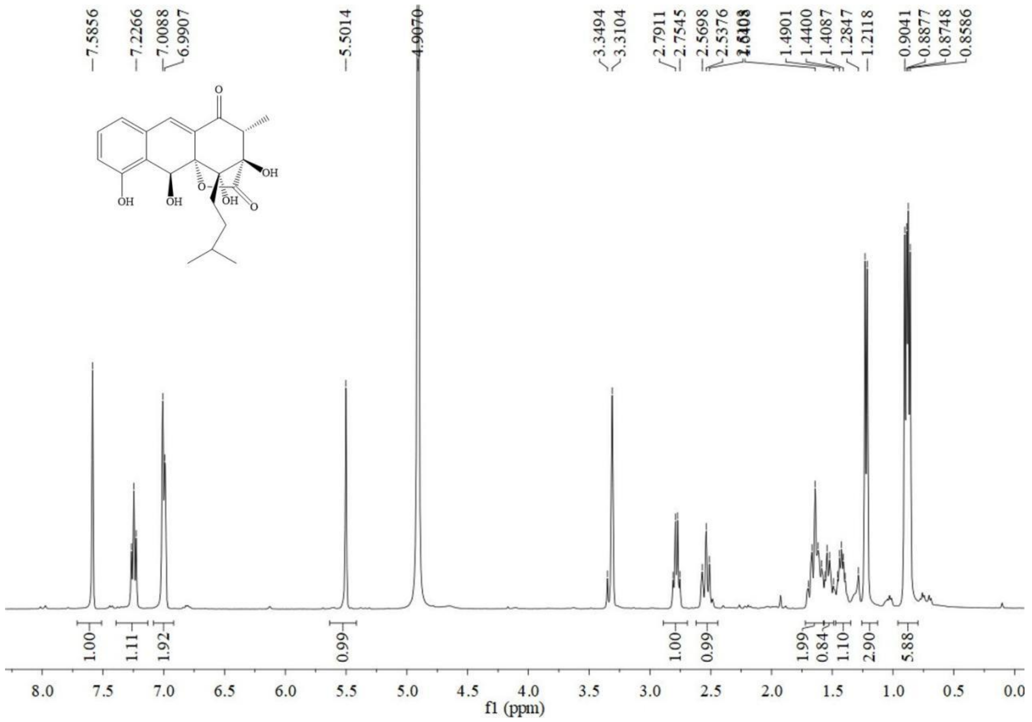

Figure S2. ^{1}H NMR spectrum of rishirilide A in methanol-d_4.

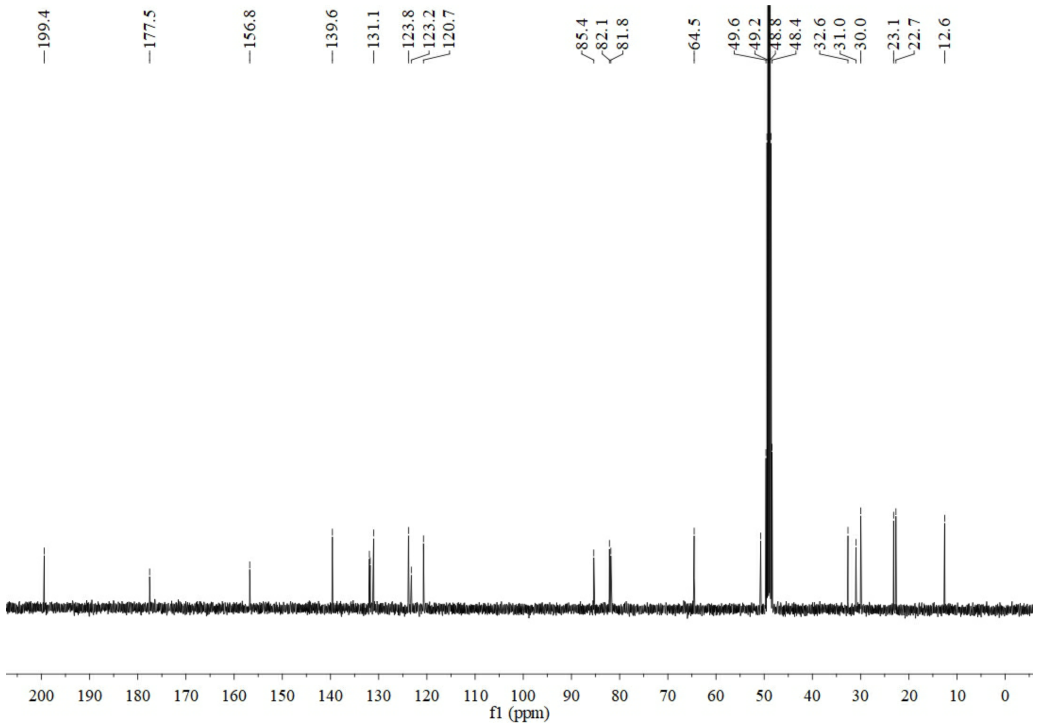

Figure S3. ^{13}C NMR spectrum of rishirilide A in methanol-d_4.

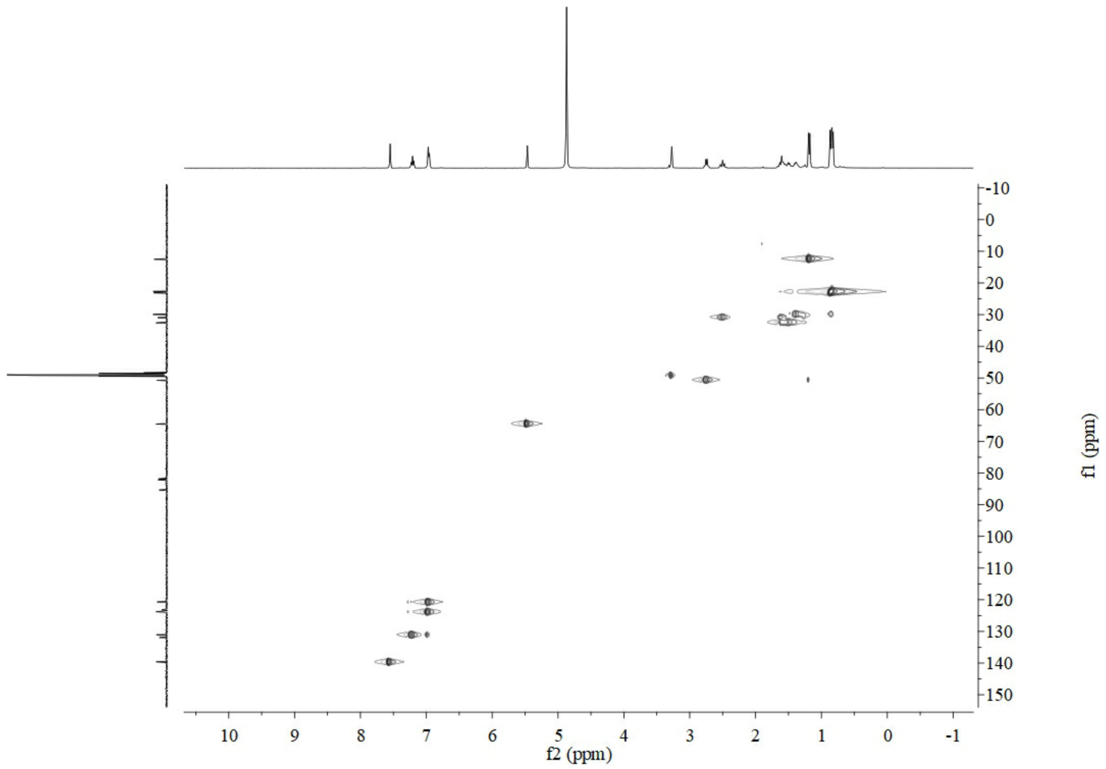

Figure S4. HSQC spectrum of rishirilide A in methanol-d_4.

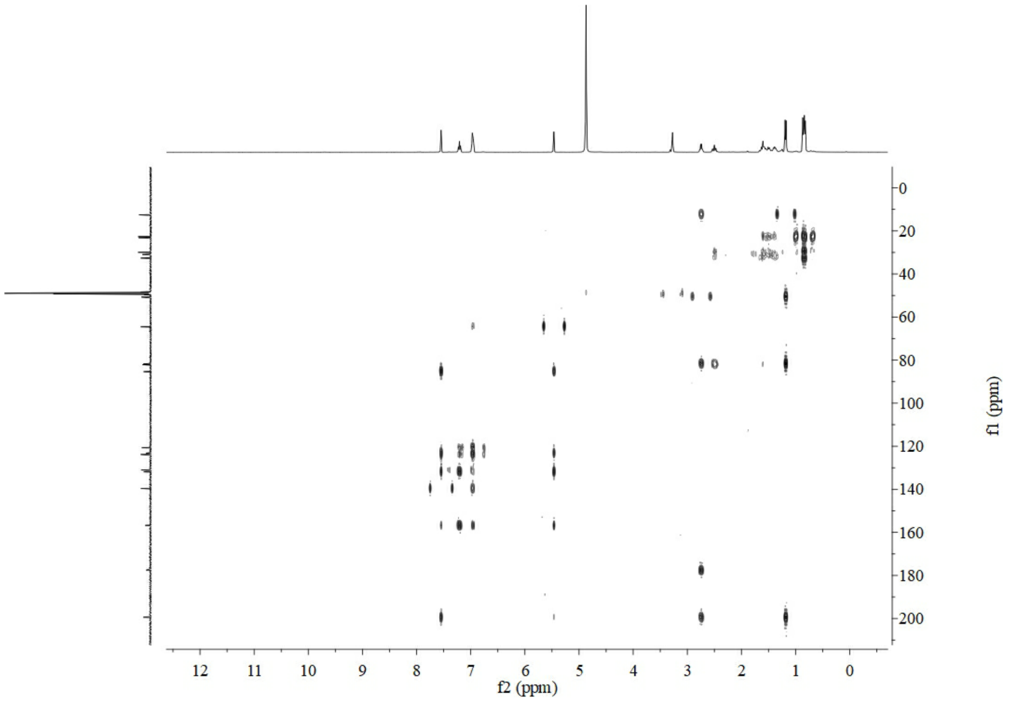

Figure S5. HMBC spectrum of rishirilide A in methanol-d_4.

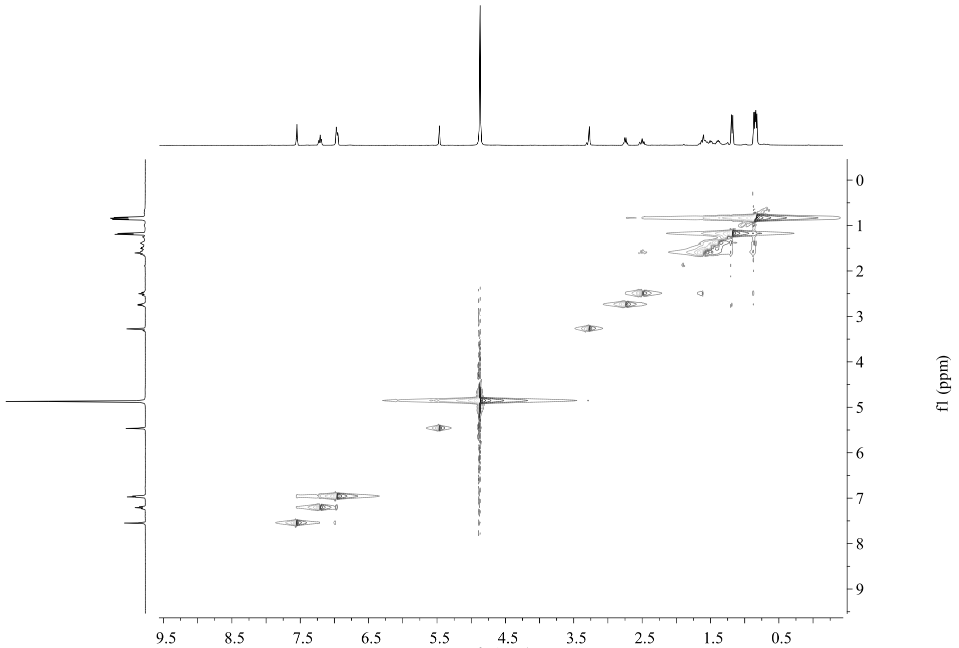

Figure S6. ROESY spectrum of rishirilide A in methanol-d_4.

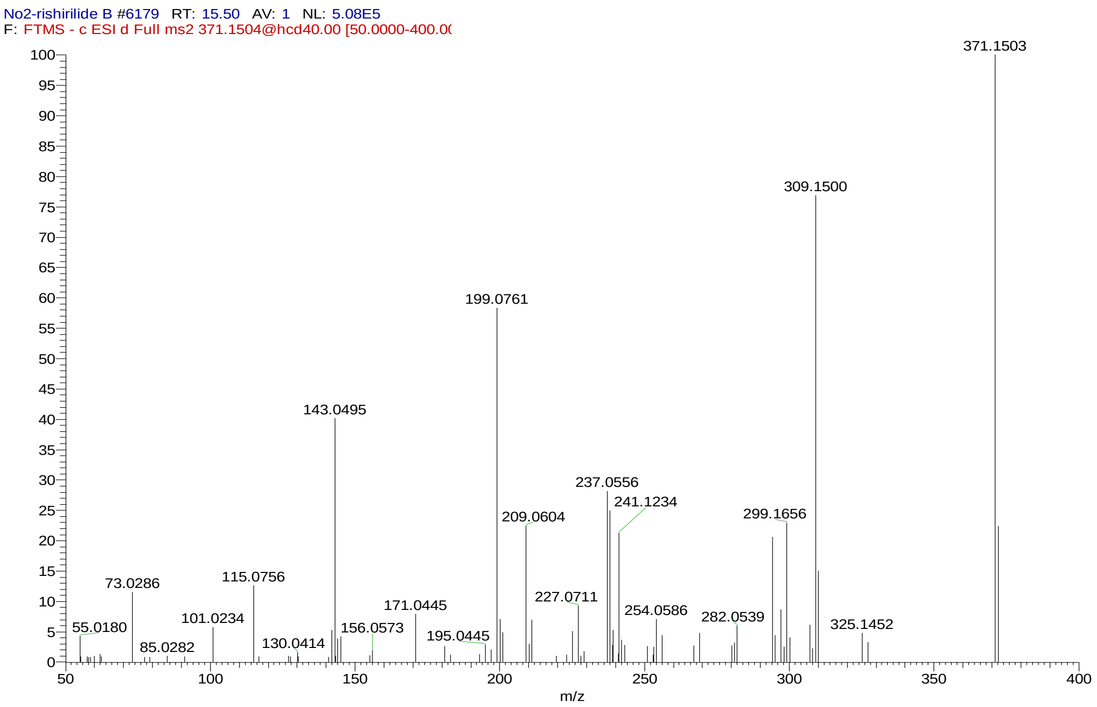

Figure S7. The HR-ESIMS chromatogram of rishirilide B.

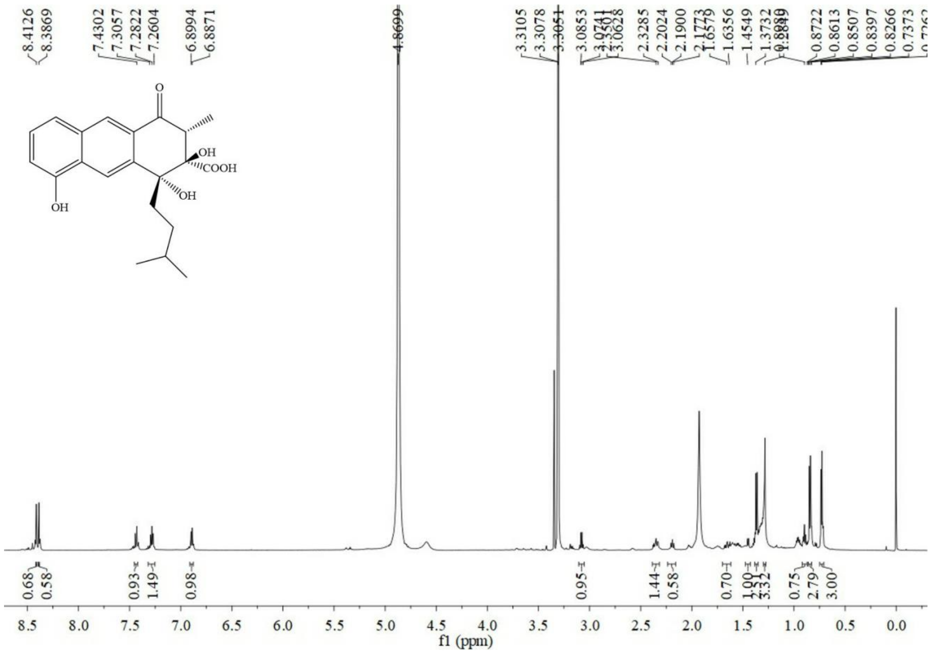

Figure S8. ^{1}H NMR spectrum of rishirilide B in methanol-d_4.

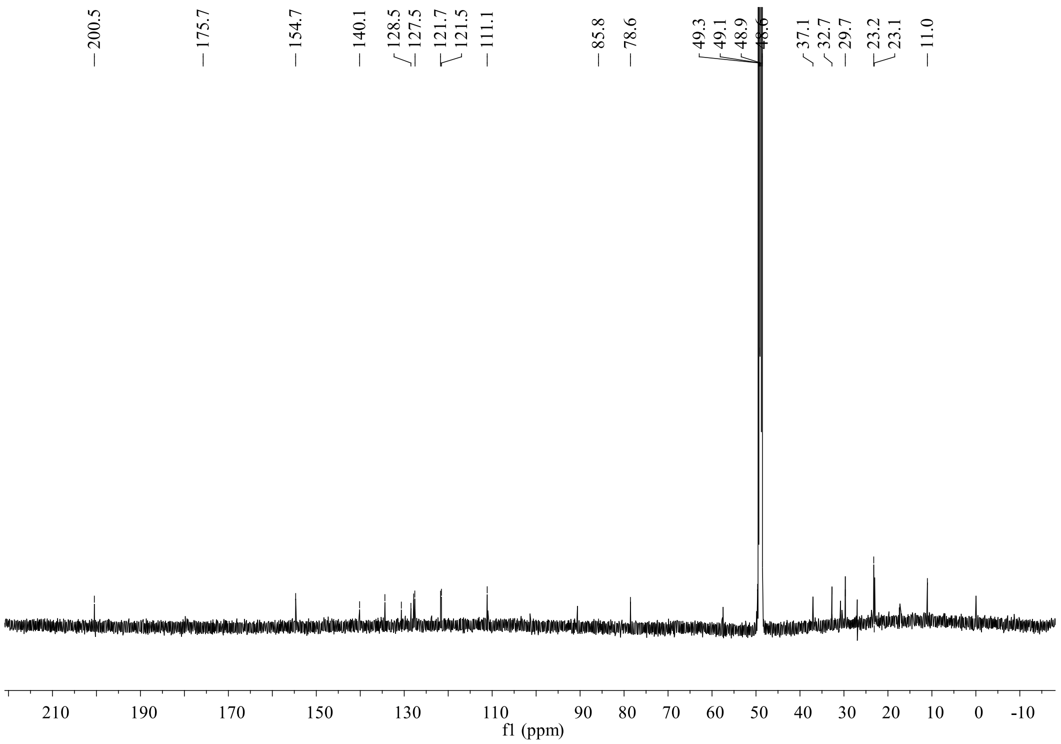

Figure S9. ^{13}C NMR spectrum of rishirilide B in methanol-d_4.

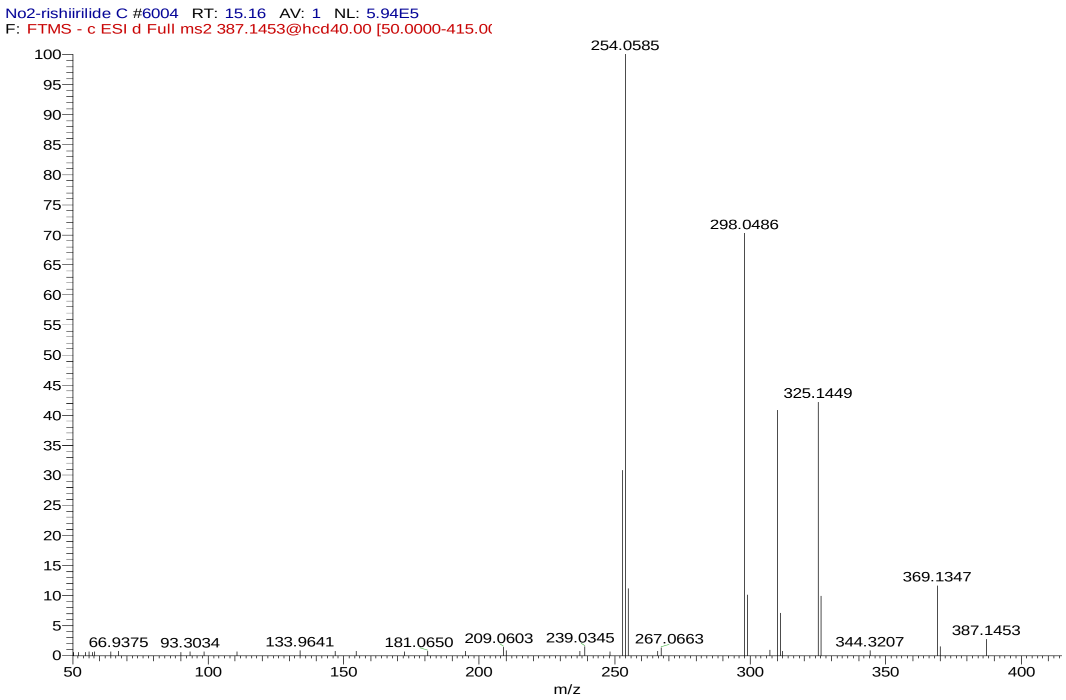

Figure S10. The HR-ESIMS chromatogram of rishirilide C.

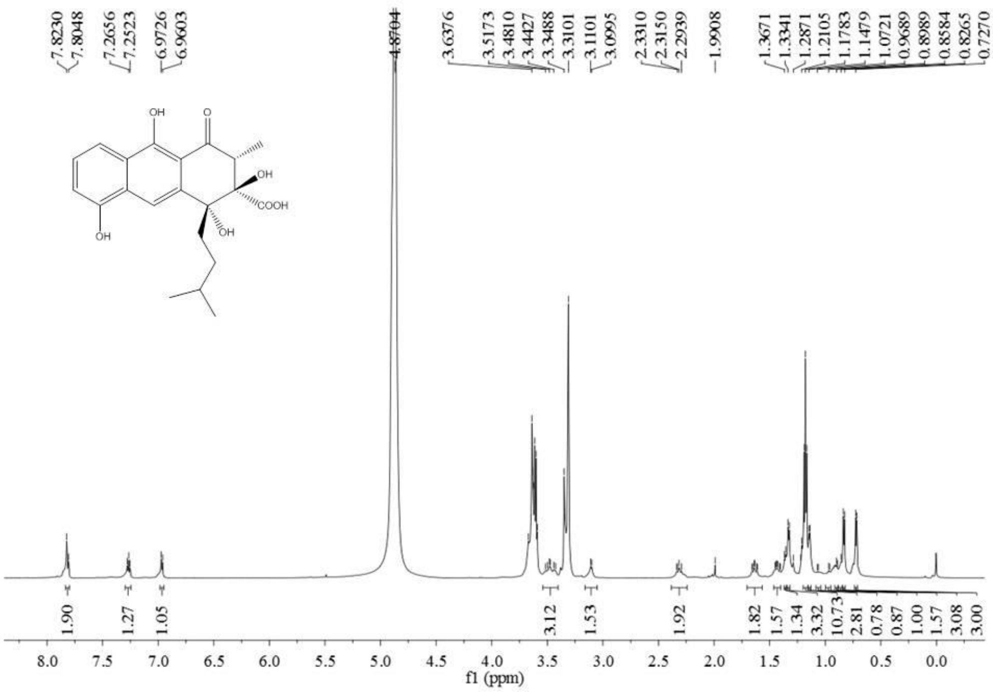

Figure S11. ^{1}H NMR spectrum of rishirilide C in methanol-d_4.

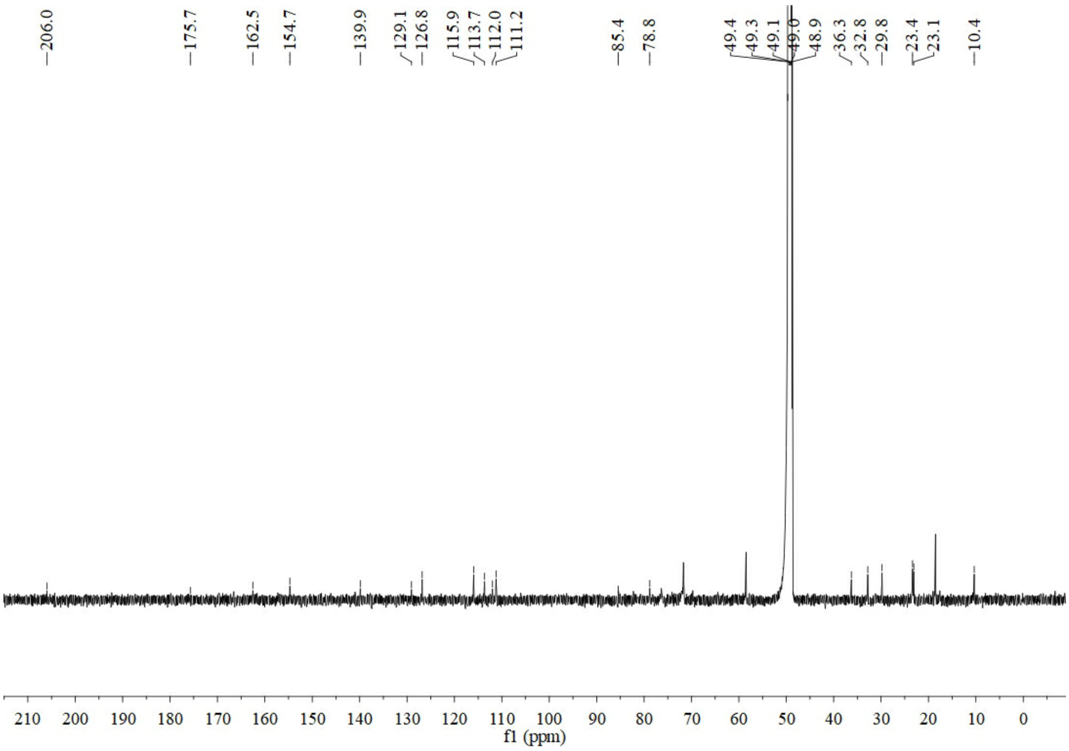

Figure S12. ^{13}C NMR spectrum of rishirilide C in methanol-d_4.

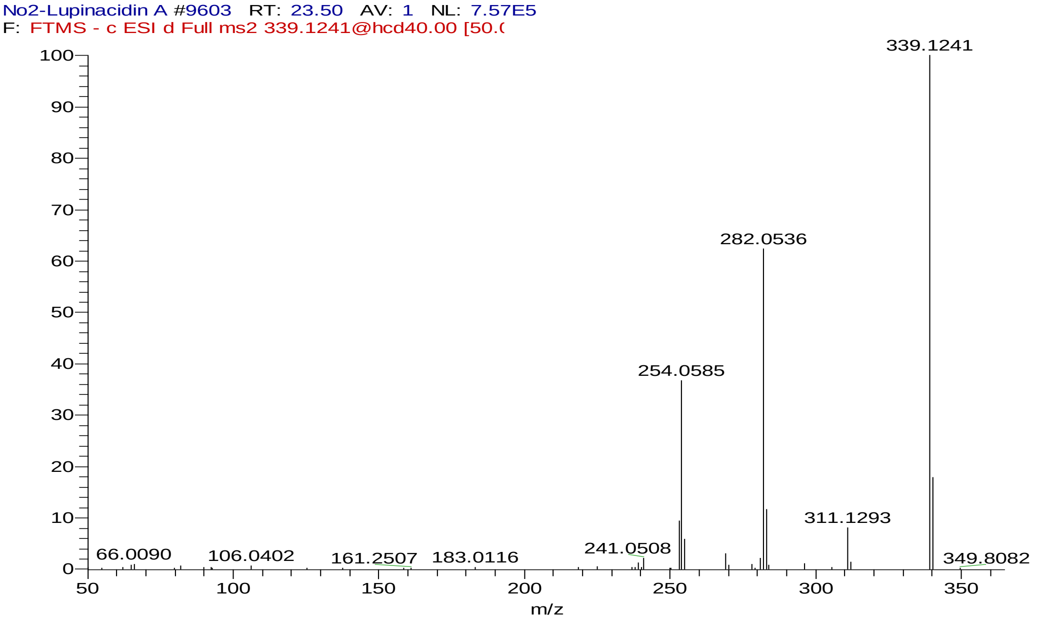

Figure S13. The HR-ESIMS chromatogram of lupinacidin A.

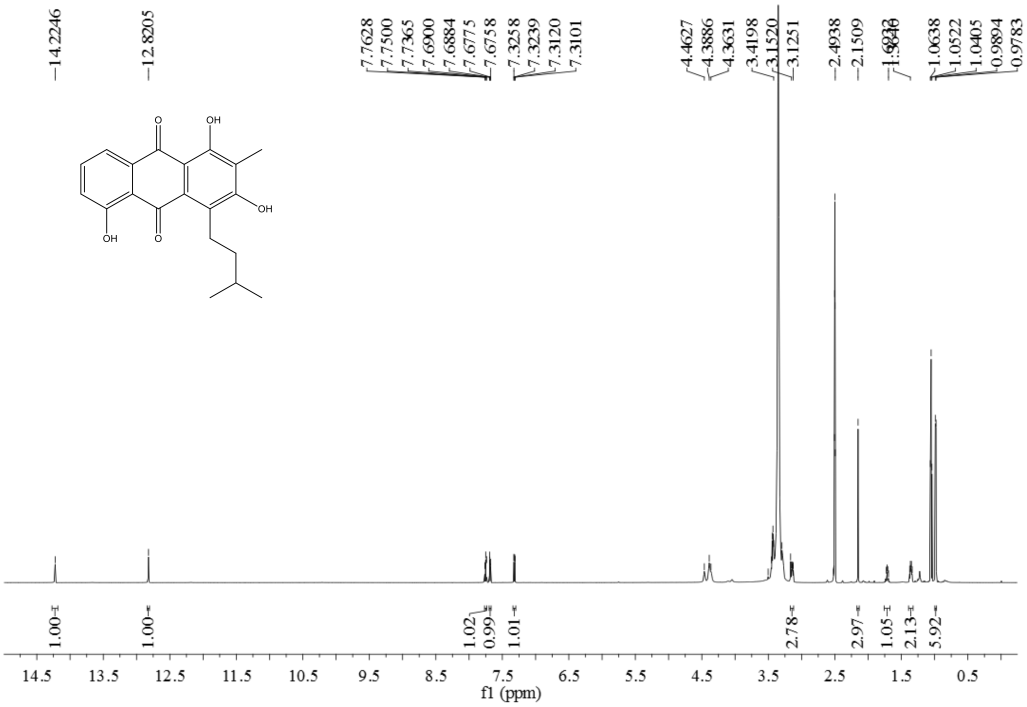

Figure S14. ^{1}H NMR spectrum of lupinacidin A in DMSO-d_6.

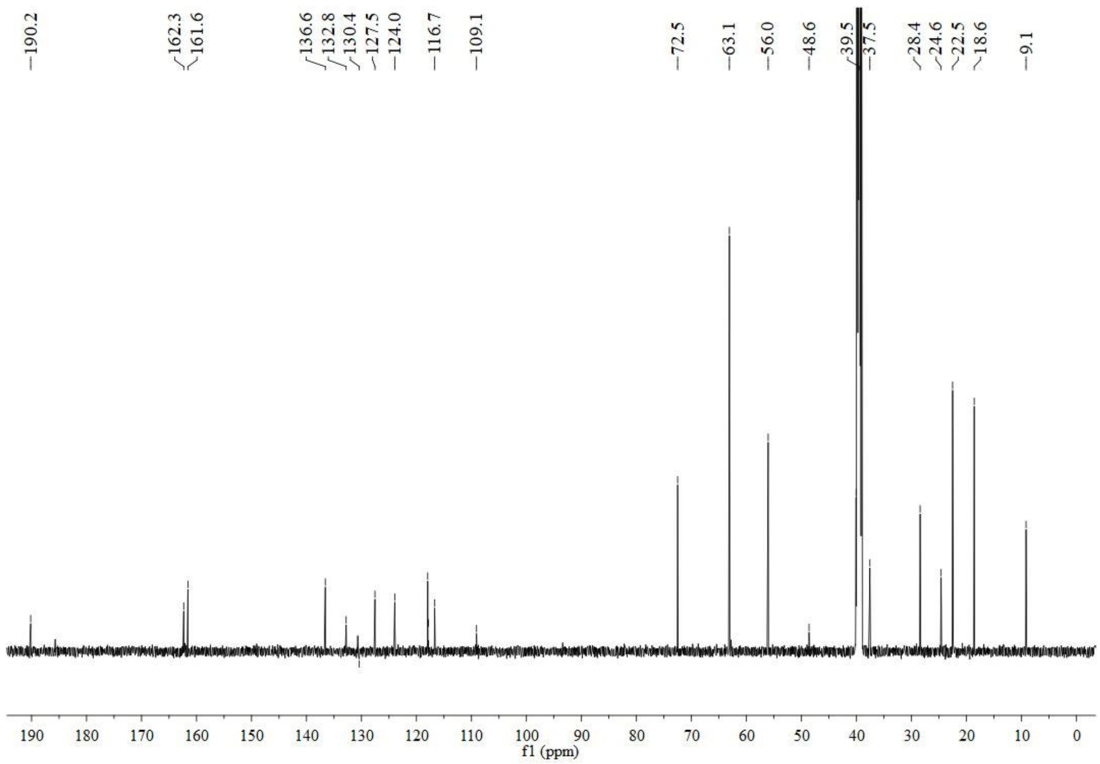

Figure S15. ^{13}C NMR spectrum of lupinacidin A in DMSO-d_6.

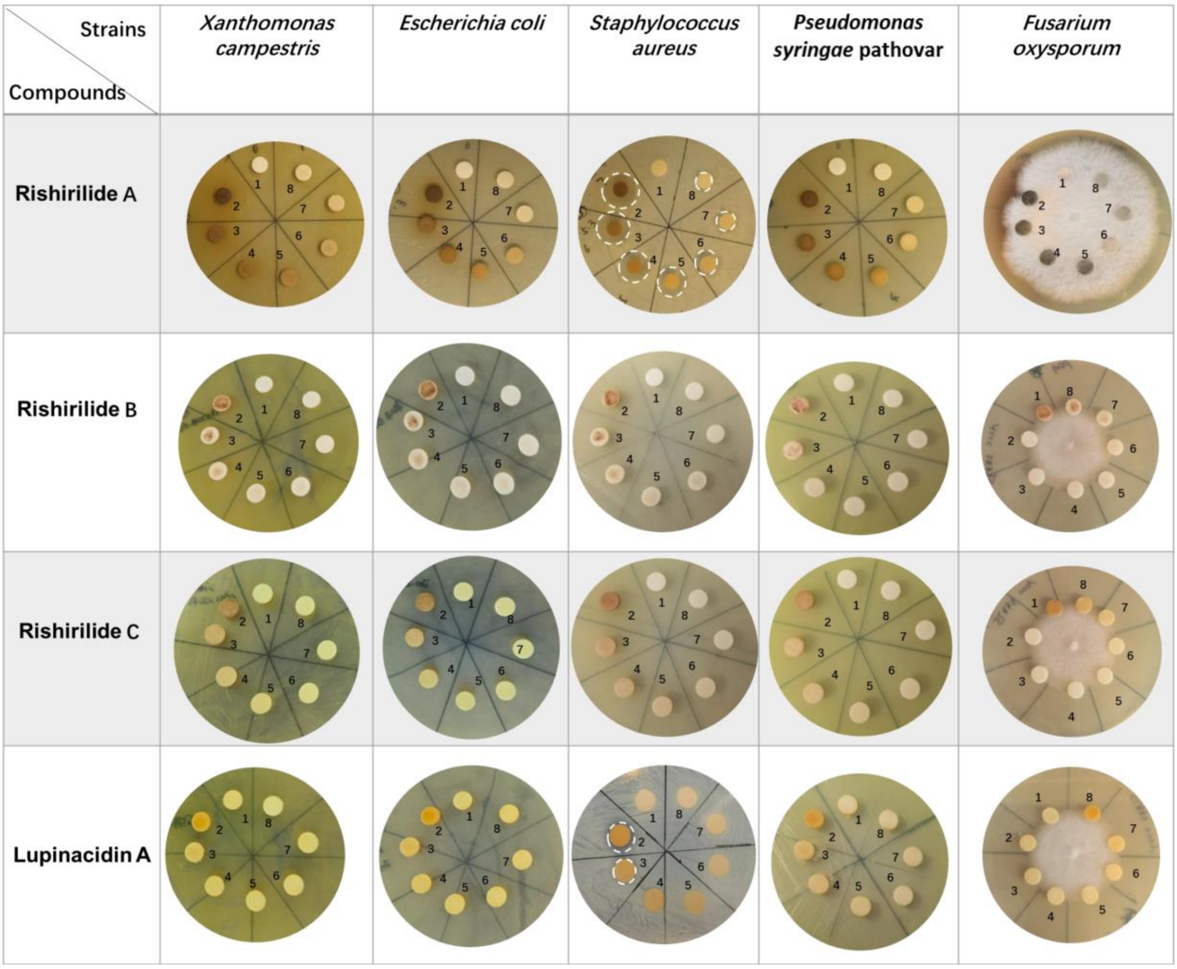

Figure S16. The antibacterial tests of rishirilide derivatives. The methanol was used as the blank control (No.1). The quantity of samples was set as 200 μg (No.2), 100 μg (No.3), 50 μg (No.4), 25 μg (No.5), 12.5 μg (No.6), 6.25 μg (No.7), 3.125 μg (No.8), respectively. The white dotted cycles were used to label the sample with antibacterial activity.

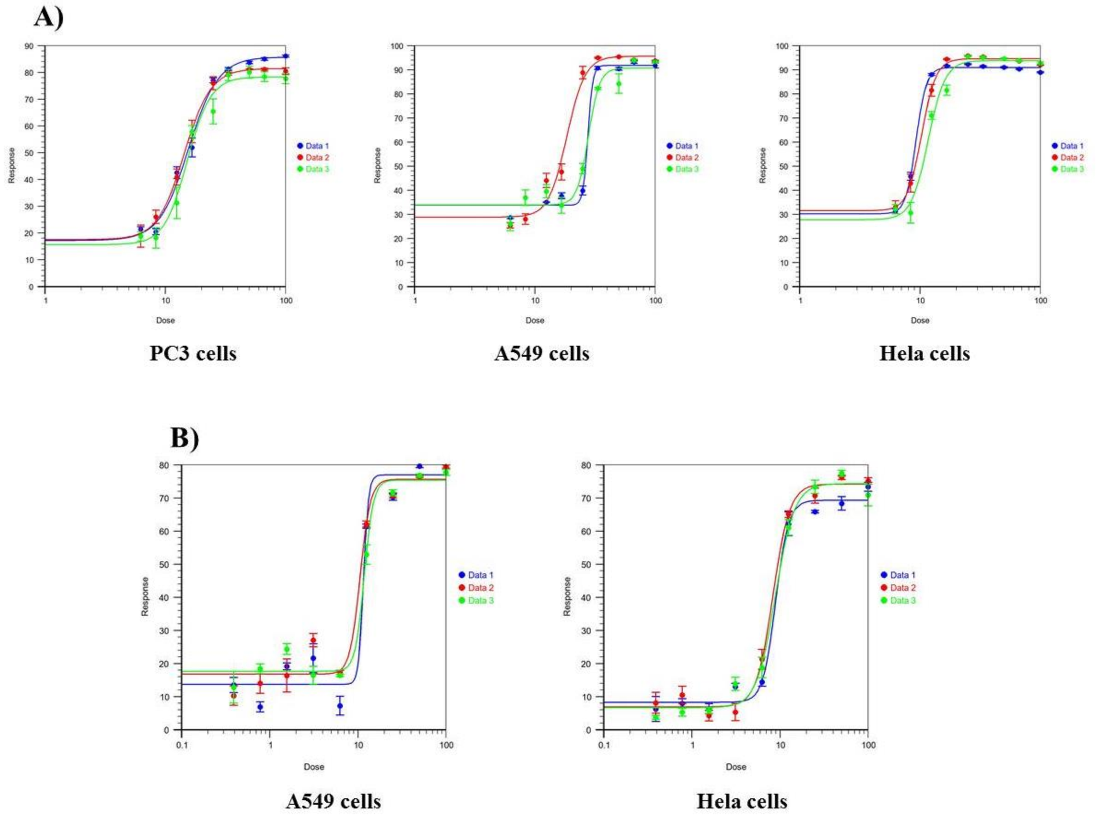

Figure S17. The inhibitory curve of compound rishirilide A (A) and lupinacidin A (B).

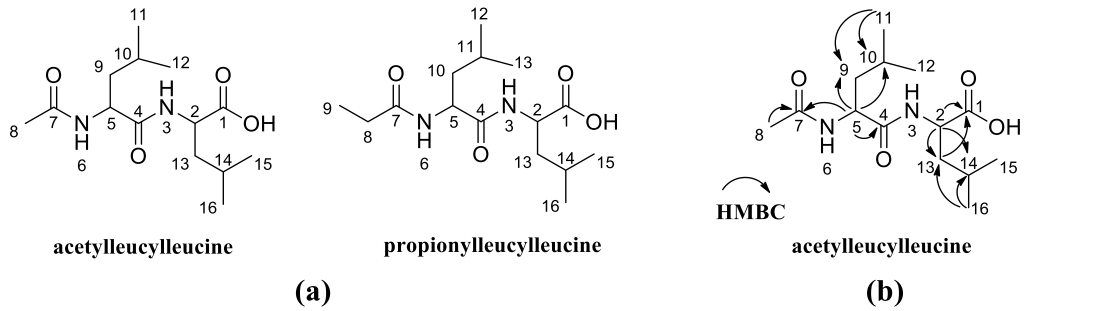

Figure S18. (a) Secondary metabolites isolated from the crude extracts of *S. xanthophaeus* no2Δ*sxrX*; (b) selected HMBC correlations of acetylleucylleucine.

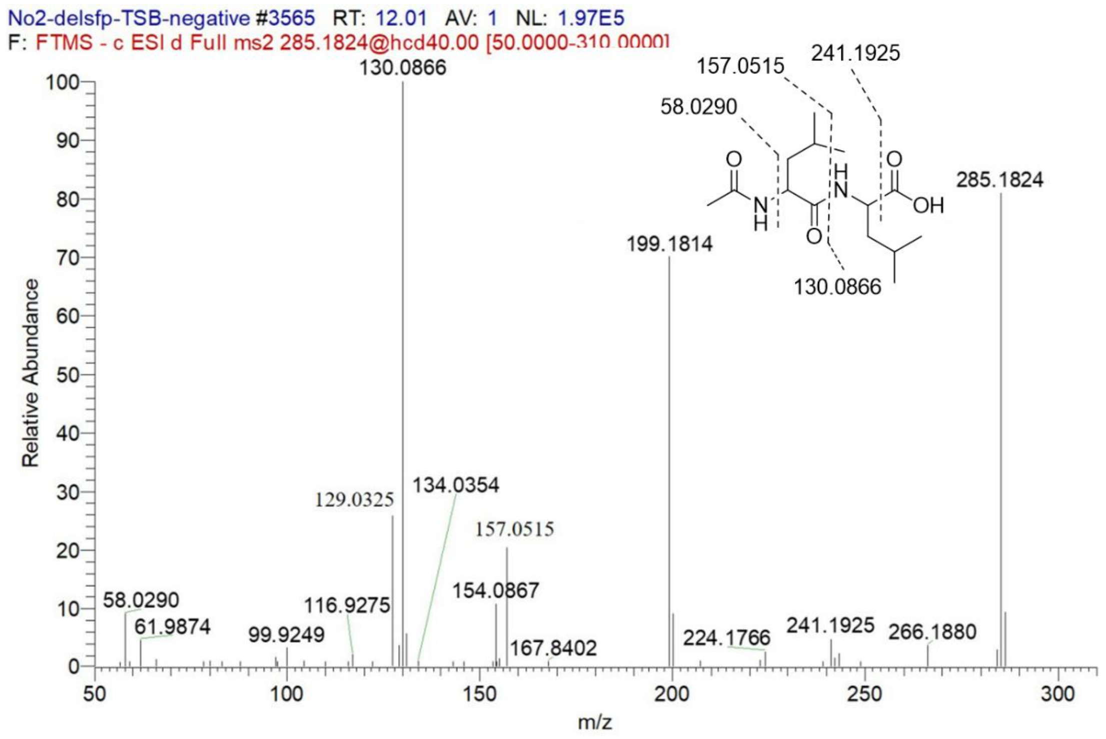

Figure S19. The HR-ESIMSMS chromatogram of acetylleucylleucine (**I**).

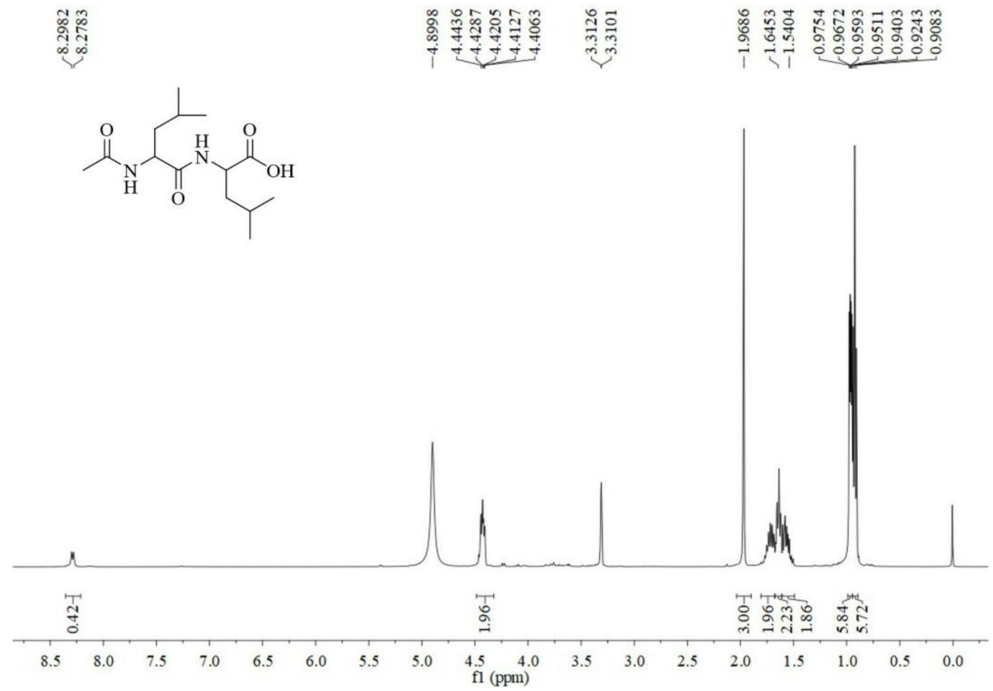

Figure S20. ^{1}H NMR spectrum of acetylleucylleucine (**I**) in methanol-d_4.

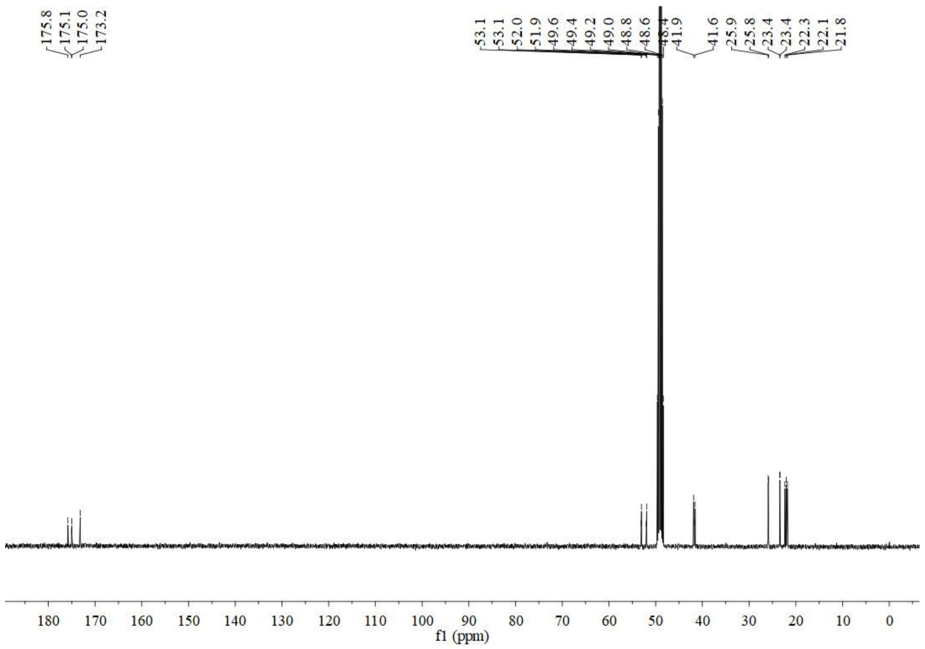

Figure S21. ^{13}C NMR spectrum of acetylleucylleucine (**I**) in methanol-d_4.

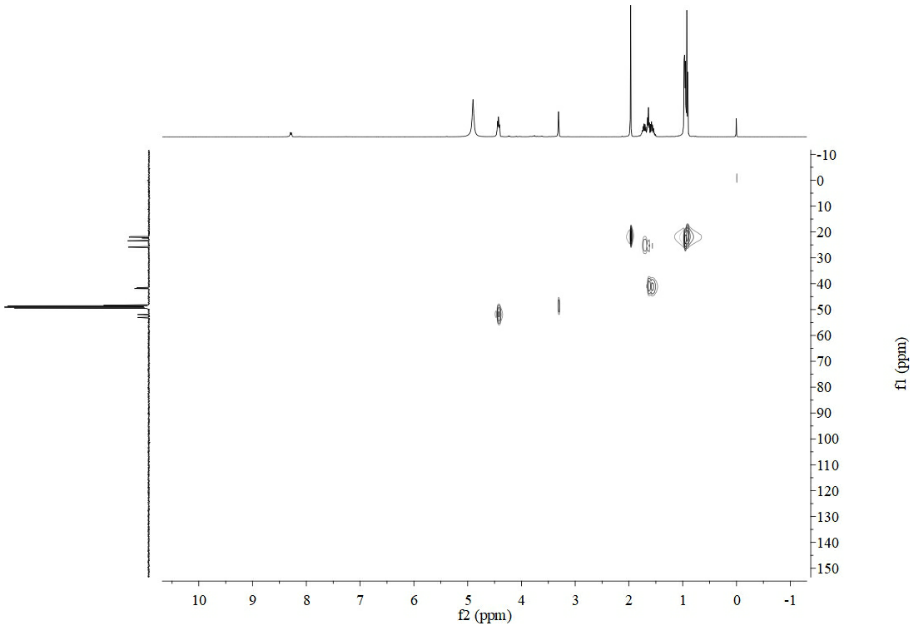

Figure S22. HSQC spectrum of acetylleucylleucine (**I**).

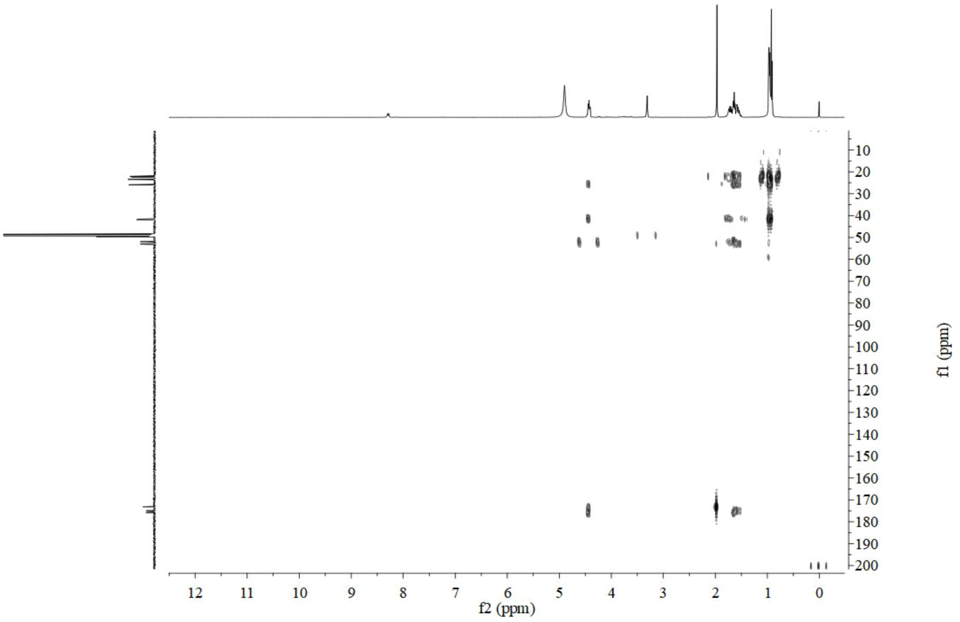

Figure S23. HMBC spectrum of acetylleucylleucine (**I**).

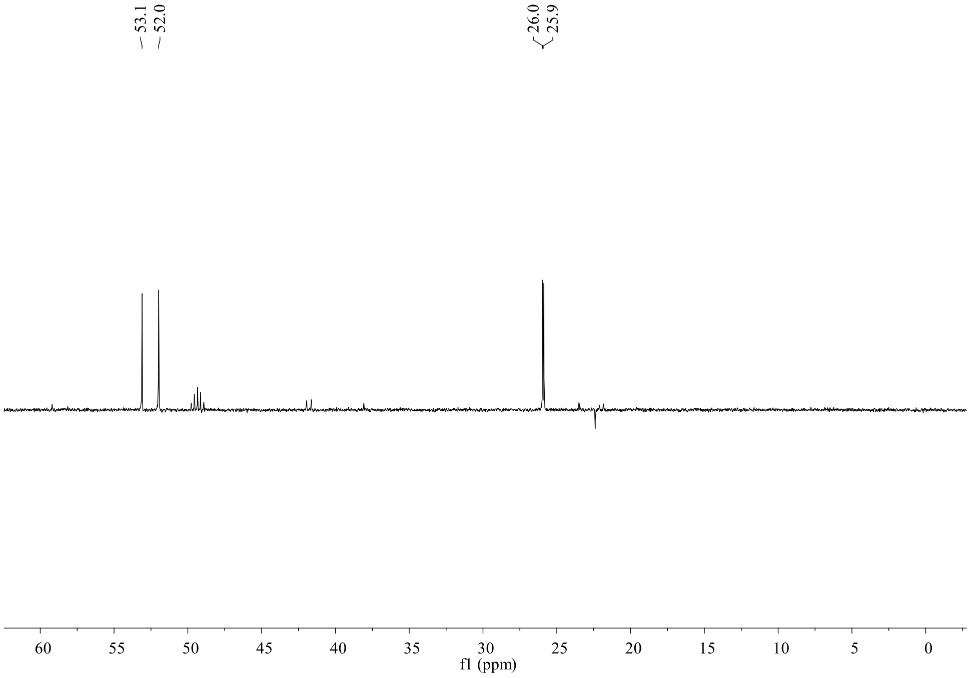

Figure S24. DEPT 90 spectrum of acetylleucylleucine (**I**).

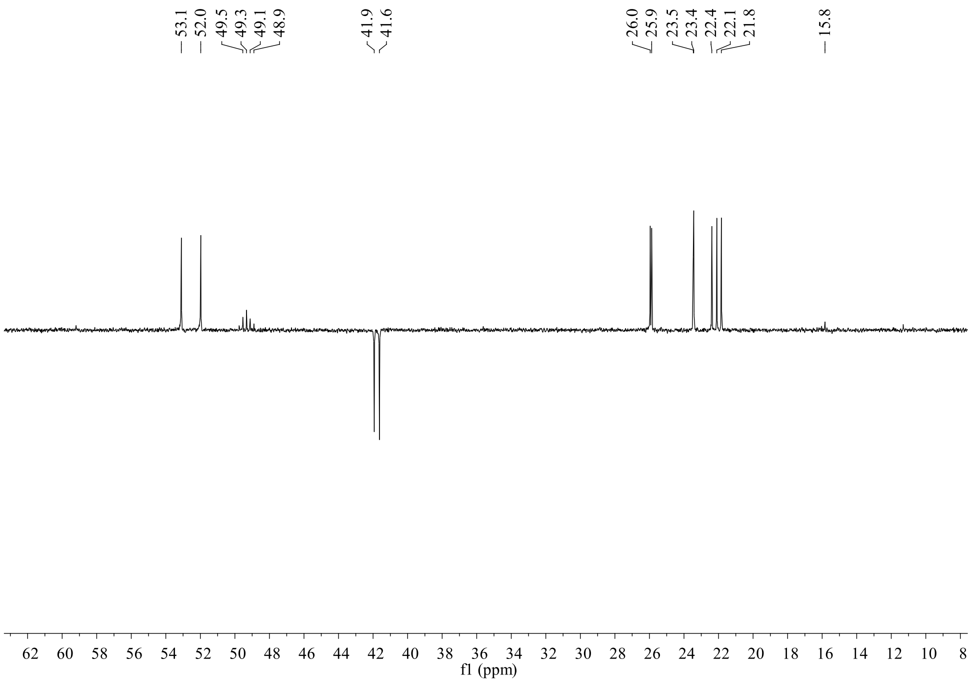

Figure S25. DEPT 135 spectrum of acetylleucylleucine (**I**).

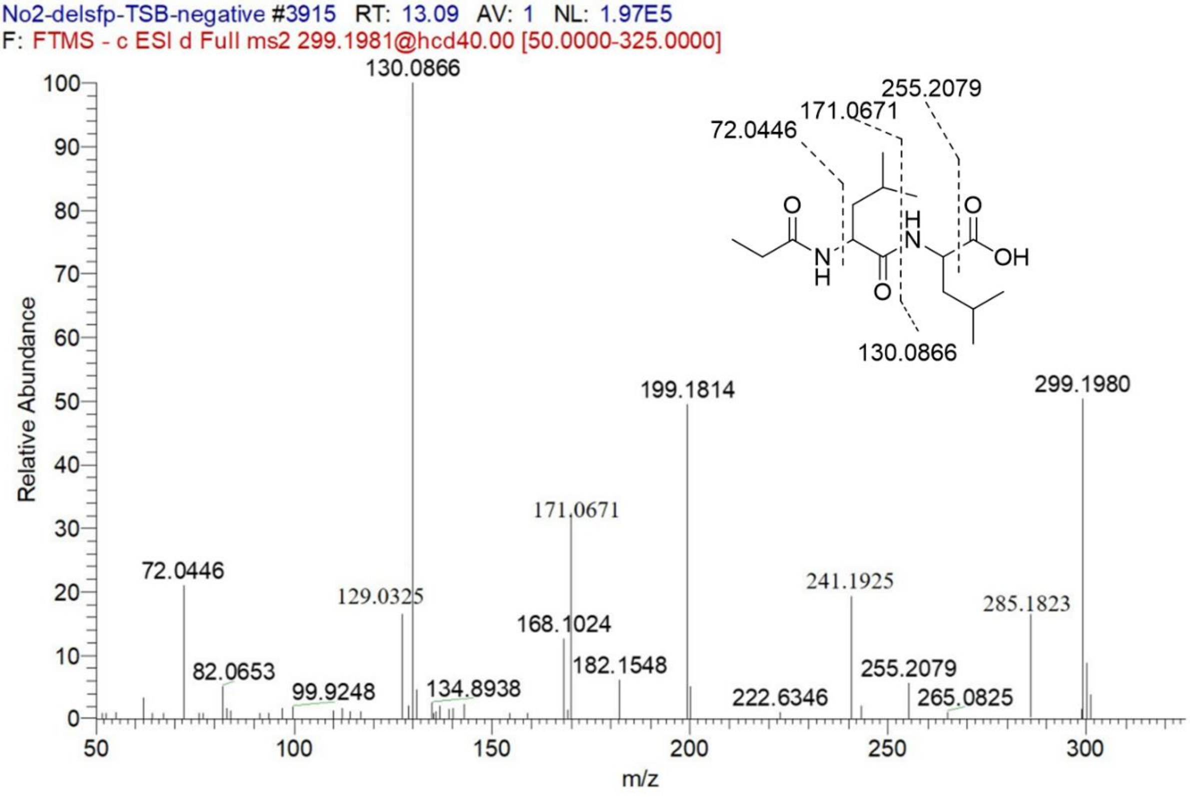

Figure S26. The HR-ESIMSMS chromatogram of propionylleucylleucine (**II**).

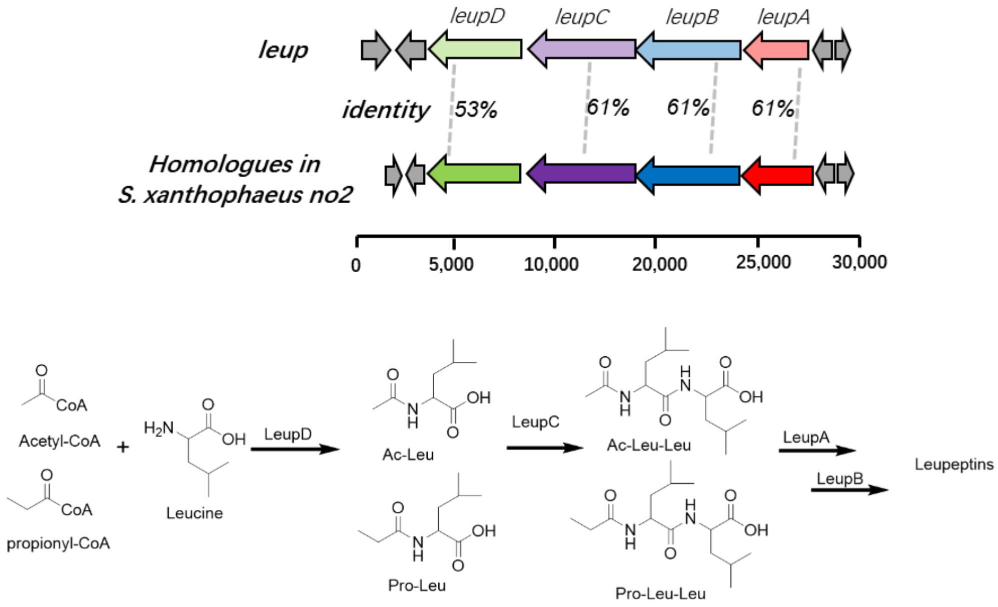

Figure S27. The putative gene cluster and possible biosynthetic pathway of acetylleucylleucine (Ac-Leu-Leu) and propionylleucylleucine (Pro-Leu-Leu) in *S. xanthophaeus* no2Δ*sxrX*. The *leup* represents the biosynthetic gene cluster of leupeptins [5].

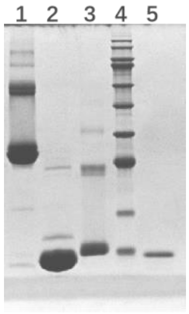

Figure S28. The SDS-PAGE result of proteins SxrX (lane 1), SxrK1 (lane 2), PCP_{ws9326} (lane 3), marker (lane 4) and ACP from fatty acid (lane 5). The related proteins were all expressed and purified, as the SDS-PAGE shown.

```
>sxrX (ctg007-1006)
MIPRRVTLWQGRSEDRADTLAADAALLAPDELERLRRLRTPLAARRYAAAHAAVRRRLGAVLDVDPVRVPLGRGPCPVCGERRHGPPVVRMPADGPALSYSLSRS
GPHWMLAVAPGHPVGVDLERERPLDVDAVARAAFSARERRAVAGAGGRALAFRCWTRKEAVAKATGHGIAMDLRAVETRPRDPGPVRVAAGWQVYSLFPGGSLHA
AVAVPEGEPGPVCRW
>ctg046-3754
MTAAAVDAGFRPIAPGRVHRWGGARLIVARAGELRQVPPLSPAERRLVRGMRAWRQAEWTAGRLLAKRLLGEVVSAPAEGVEILPRDDGSPRVVVGGTPAHALYV
SISHTAGHVAAALAPVPVGVDLCERDSADAVRRVADHFLSPEELSLVGRERPDAMTGAWALKEAAVKADRSSVFGAAPRGIPILGIRPPLLGGRRRAMVWRADDA
ALALVLAHPGG
>ctg073-4969
MTTPAGLSSAVLRLDRPVGAQRLPARGEALVRFVDAGTQAPFAERLAPGVLDPAERRRADRFARPQDRGSYLVAHVALRLMLGALLDTAPRDLVMTRDACPECGG
PDGRPVLVGGRAHFSLSHSRDAVFVACASTPVGVDVEALPAPRVVAQSEDFFHPDESAELAAFPEAGRPAAFARLWTRKEAHLKGTGAGLGHGGHRTYLGTGPAA
DSVRPHWSLTDLPAPEGYAAALALRAPSAHWR
>ctg040-3497
MIEYVNQLGPGAPRIGPPPSGTAASVWSLDTTAGTVGGYRVEEADAVLDAGEREKAARLVLPGLRHRYLASHLGLRVLLGARLGLDPREVTLVREDCPCCGGPHG
RPAVAGGGVHFSLSHSGDLAYLAFAAVPVGVDIEETPGADAVADVLDSLHPAETAELTALAPPRRPAALARVWSRKEACLKAAGTGLALGTAEPYVGSATAPAPV
TGWILTDLPAPDGYAAALAVADHHHEQGNPA
>ctg022-2273
MSPDPREIFDRDATHVWTGAASGRGAVPGTRSLSLEERARTRTLPAPRAGWYAATRTAVREVLGRYLEQPPGEILLGRSPCPGCGSAAHGPPAVLAPATTLTFSL
SYSGPRWLLALTSIRPLGVDVQEVLPGAGPDLTRMAASCFAPDELGEFQARSAPASRAAYFYRAWTRKEAVVKAMGVGLAADLTGVHVSAGTPGPAVVRTRRAGH
TALWQVEDLPVSGGPGFAALAREASAAGPVRFFVHEPAEPDPEIRQNPATAAA
>ctg032-2936
MISSVLPPSVATAELFRDDPAAAPLFPEEERVVAGAVAARRREFASVRRCARDALARLGLPPAPILPGPGGAPRWPDGIVGSMTHCRGYRAAAVARRSDVLSIGC
DAEPNEPLPDAGILDLIALPQERARVRALAARRPAVCWDRLLFSAKESVYKAWFPLTGSPLDFDEALITLDPPTNTFRADLLVPGPTLNGFRVTAFNGRWRAGNG
LVVSAVTINRLP
>ctg016-1818
MIIGVGIDVAEIERFGAALERTPNLAGRLFVDAELTLPSGERRGTASLAARFAAKEALAKALGAPAGLLWTDAEVYVEDSGQPRLRVSGTVEARALALGVKSWHI
SLSHDAGVASAVVIAEG
```

Figure S29. The protein sequences of PPTase found in the *S. xanthophaeus* no2.

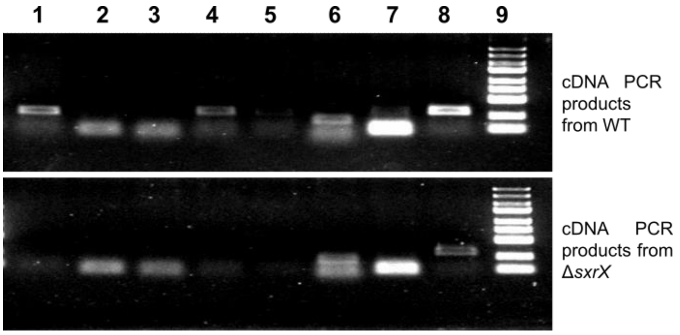

Figure S30. Transcriptional analysis of PPTase-encoding genes in *S. xanthophaeus* no2 by RT-PCR. RT-PCR analysis of co-transcribed genes. Total RNA of wildtype *S. xanthophaeus* no2 and mutant Δ*sxrX* were isolated after incubation for 72 h and used for synthesizing complementary DNA (cDNA). The genome DNA (gDNA) from WT and mutant Δ*sxrX* were used as positive control. Gene *gyrB* (301bp, lane 8) was used as the positive control. The samples from left to right: *sxrX* (ctg007-1006) (298bp, lane 1), ctg022-2273 (289bp, lane 2), ctg032-2936 (274bp, lane 3), ctg016-1818 (296bp, lane 4), ctg073-4969 (287bp, lane 5), ctg046-3754 (276bp, lane 6), ctg040-3497 (299bp, lane 7).

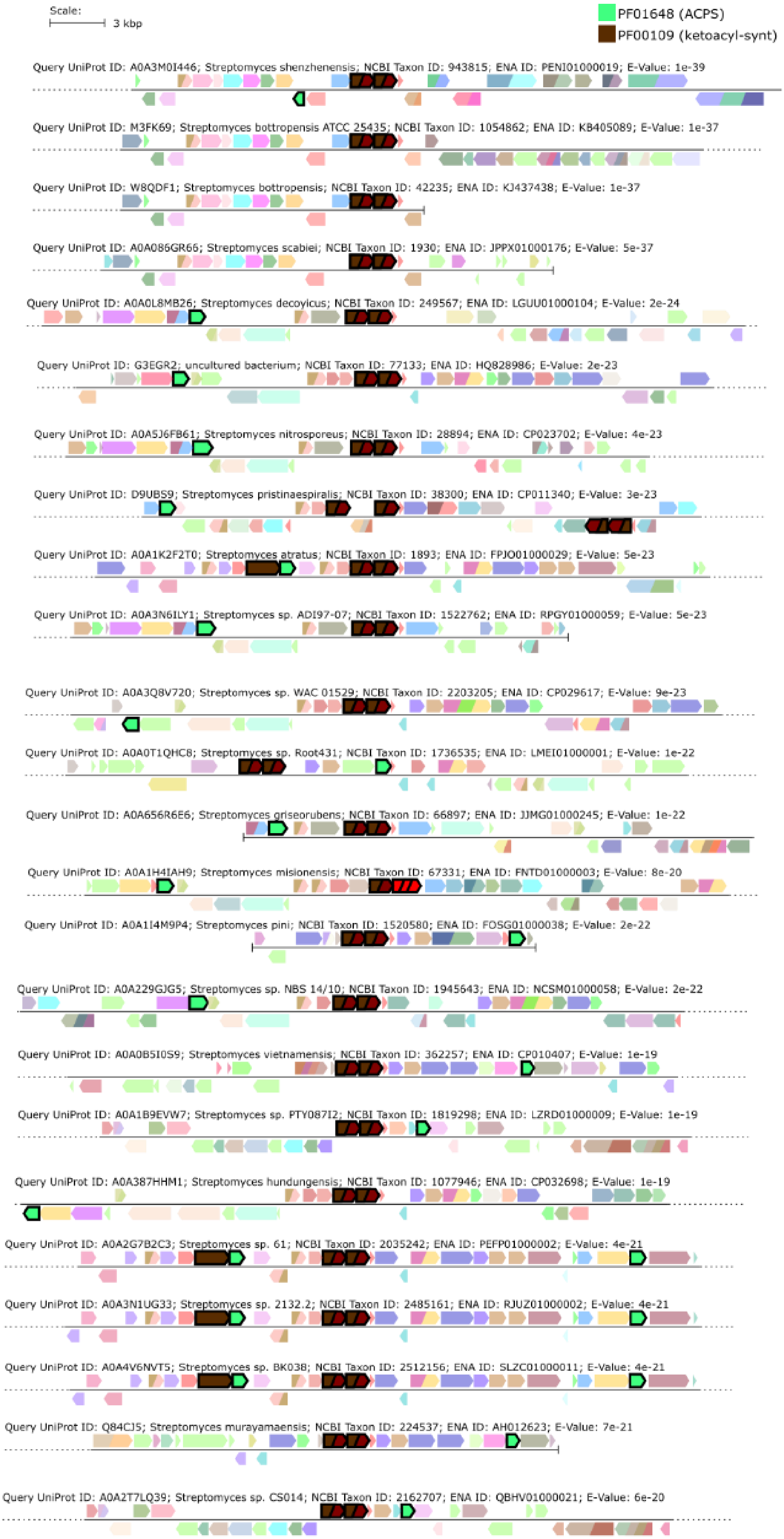

Scale:
3 kbp
PF01648 (ACPS)
PF00109 (ketoacyl-synt)
Query UniProt ID: A0A3M0I446; Streptomyces shenzhenensis; NCBI Taxon ID: 943815; ENA ID: PENI01000019; E-Value: 1e-39
Query UniProt ID: M3FK69; Streptomyces bottropensis ATCC 25435; NCBI Taxon ID: 1054862; ENA ID: KB405089; E-Value: 1e-37
Query UniProt ID: W8QDF1; Streptomyces bottropensis; NCBI Taxon ID: 42235; ENA ID: KJ437438; E-Value: 1e-37
Query UniProt ID: A0A086GR66; Streptomyces scabiei; NCBI Taxon ID: 1930; ENA ID: JPPX01000176; E-Value: 5e-37
Query UniProt ID: A0A0L8MB26; Streptomyces decoyicus; NCBI Taxon ID: 249567; ENA ID: LGUU01000104; E-Value: 2e-24
Query UniProt ID: G3EGR2; uncultured bacterium; NCBI Taxon ID: 77133; ENA ID: HQ828986; E-Value: 2e-23
Query UniProt ID: A0A5J6FB61; Streptomyces nitrosporeus; NCBI Taxon ID: 28894; ENA ID: CP023702; E-Value: 4e-23
Query UniProt ID: D9UBS9; Streptomyces pristinaespiralis; NCBI Taxon ID: 38300; ENA ID: CP011340; E-Value: 3e-23
Query UniProt ID: A0A1K2F2T0; Streptomyces atratus; NCBI Taxon ID: 1893; ENA ID: FPJO01000029; E-Value: 5e-23
Query UniProt ID: A0A3N6ILY1; Streptomyces sp. ADI97-07; NCBI Taxon ID: 1522762; ENA ID: RPGY01000059; E-Value: 5e-23
Query UniProt ID: A0A3Q8V720; Streptomyces sp. WAC 01529; NCBI Taxon ID: 2203205; ENA ID: CP029617; E-Value: 9e-23
Query UniProt ID: A0A0T1QHC8; Streptomyces sp. Root431; NCBI Taxon ID: 1736535; ENA ID: LMEI01000001; E-Value: 1e-22
Query UniProt ID: A0A656R6E6; Streptomyces griseorubens; NCBI Taxon ID: 66897; ENA ID: JJMG01000245; E-Value: 1e-22
Query UniProt ID: A0A1H4IAH9; Streptomyces misionensis; NCBI Taxon ID: 67331; ENA ID: FNTD01000003; E-Value: 8e-20
Query UniProt ID: A0A1I4M9P4; Streptomyces pini; NCBI Taxon ID: 1520580; ENA ID: FOSG01000038; E-Value: 2e-22
Query UniProt ID: A0A229GJG5; Streptomyces sp. NBS 14/10; NCBI Taxon ID: 1945643; ENA ID: NCSM01000058; E-Value: 2e-22
Query UniProt ID: A0A0B5I0S9; Streptomyces vietnamensis; NCBI Taxon ID: 362257; ENA ID: CP010407; E-Value: 1e-19
Query UniProt ID: A0A1B9EVW7; Streptomyces sp. PTY087I2; NCBI Taxon ID: 1819298; ENA ID: LZRD01000009; E-Value: 1e-19
Query UniProt ID: A0A387HHM1; Streptomyces hundungensis; NCBI Taxon ID: 1077946; ENA ID: CP032698; E-Value: 1e-19
Query UniProt ID: A0A2G7B2C3; Streptomyces sp. 61; NCBI Taxon ID: 2035242; ENA ID: PEFP01000002; E-Value: 4e-21
Query UniProt ID: A0A3N1UG33; Streptomyces sp. 2132.2; NCBI Taxon ID: 2485161; ENA ID: RJUZ01000002; E-Value: 4e-21
Query UniProt ID: A0A4V6NVT5; Streptomyces sp. BK038; NCBI Taxon ID: 2512156; ENA ID: SLZC01000011; E-Value: 4e-21
Query UniProt ID: Q84CJ5; Streptomyces murayamaensis; NCBI Taxon ID: 224537; ENA ID: AH012623; E-Value: 7e-21
Query UniProt ID: A0A2T7LQ39; Streptomyces sp. CS014; NCBI Taxon ID: 2162707; ENA ID: QBHV01000021; E-Value: 6e-20

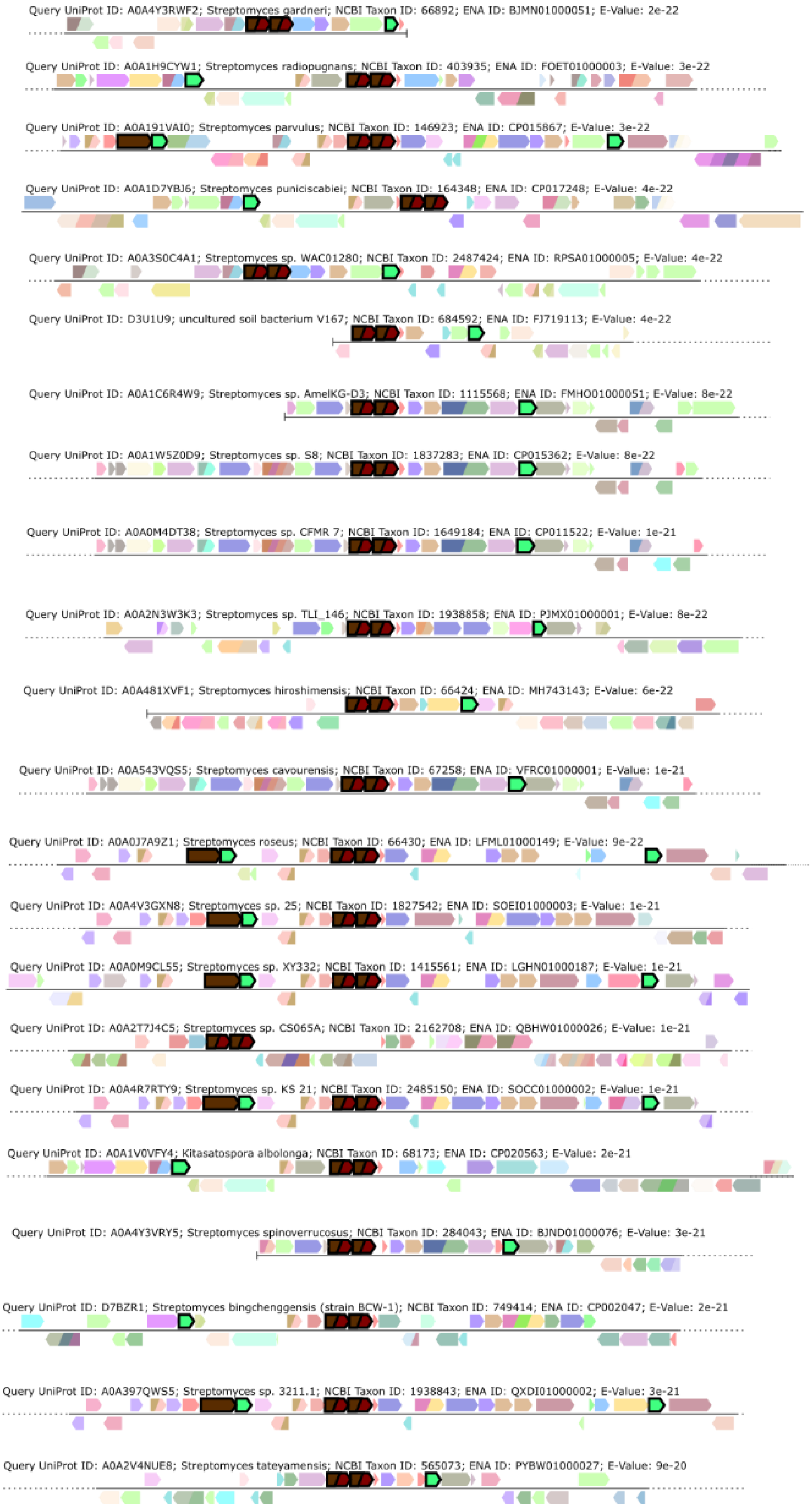
Query UniProt ID: A0A4Y3RWF2; Streptomyces gardneri; NCBI Taxon ID: 66892; ENA ID: BJMN01000051; E-Value: 2e-22
Query UniProt ID: A0A1H9CYW1; Streptomyces radiopugnans; NCBI Taxon ID: 403935; ENA ID: FOET01000003; E-Value: 3e-22
Query UniProt ID: A0A191VAI0; Streptomyces parvulus; NCBI Taxon ID: 146923; ENA ID: CP015867; E-Value: 3e-22
Query UniProt ID: A0A1D7YBJ6; Streptomyces puniciscabiei; NCBI Taxon ID: 164348; ENA ID: CP017248; E-Value: 4e-22
Query UniProt ID: A0A3S0C4A1; Streptomyces sp. WAC01280; NCBI Taxon ID: 2487424; ENA ID: RPSA01000005; E-Value: 4e-22
Query UniProt ID: D3U1U9; uncultured soil bacterium V167; NCBI Taxon ID: 684592; ENA ID: FJ719113; E-Value: 4e-22
Query UniProt ID: A0A1C6R4W9; Streptomyces sp. AmelKG-D3; NCBI Taxon ID: 1115568; ENA ID: FMHO01000051; E-Value: 8e-22
Query UniProt ID: A0A1W5Z0D9; Streptomyces sp. S8; NCBI Taxon ID: 1837283; ENA ID: CP015362; E-Value: 8e-22
Query UniProt ID: A0A0M4DT38; Streptomyces sp. CFMR 7; NCBI Taxon ID: 1649184; ENA ID: CP011522; E-Value: 1e-21
Query UniProt ID: A0A2N3W3K3; Streptomyces sp. TLI_146; NCBI Taxon ID: 1938858; ENA ID: PJMX01000001; E-Value: 8e-22
Query UniProt ID: A0A481XVF1; Streptomyces hiroshimensis; NCBI Taxon ID: 66424; ENA ID: MH743143; E-Value: 6e-22
Query UniProt ID: A0A543VQS5; Streptomyces cavourensis; NCBI Taxon ID: 67258; ENA ID: VFRC01000001; E-Value: 1e-21
Query UniProt ID: A0A0J7A9Z1; Streptomyces roseus; NCBI Taxon ID: 66430; ENA ID: LFML01000149; E-Value: 9e-22
Query UniProt ID: A0A4V3GXN8; Streptomyces sp. 25; NCBI Taxon ID: 1827542; ENA ID: SOEI01000003; E-Value: 1e-21
Query UniProt ID: A0A0M9CL55; Streptomyces sp. XY332; NCBI Taxon ID: 1415561; ENA ID: LGHN01000187; E-Value: 1e-21
Query UniProt ID: A0A2T7J4C5; Streptomyces sp. CS065A; NCBI Taxon ID: 2162708; ENA ID: QBHW01000026; E-Value: 1e-21
Query UniProt ID: A0A4R7RTY9; Streptomyces sp. KS 21; NCBI Taxon ID: 2485150; ENA ID: SOCC01000002; E-Value: 1e-21
Query UniProt ID: A0A1V0VFY4; Kitasatospora albolonga; NCBI Taxon ID: 68173; ENA ID: CP020563; E-Value: 2e-21
Query UniProt ID: A0A4Y3VRY5; Streptomyces spinoverrucosus; NCBI Taxon ID: 284043; ENA ID: BJND01000076; E-Value: 3e-21
Query UniProt ID: D7BZR1; Streptomyces bingchenggensis (strain BCW-1); NCBI Taxon ID: 749414; ENA ID: CP002047; E-Value: 2e-21
Query UniProt ID: A0A397QWS5; Streptomyces sp. 3211.1; NCBI Taxon ID: 1938843; ENA ID: QXDI01000002; E-Value: 3e-21
Query UniProt ID: A0A2V4NUE8; Streptomyces tateyamensis; NCBI Taxon ID: 565073; ENA ID: PYBW01000027; E-Value: 9e-20

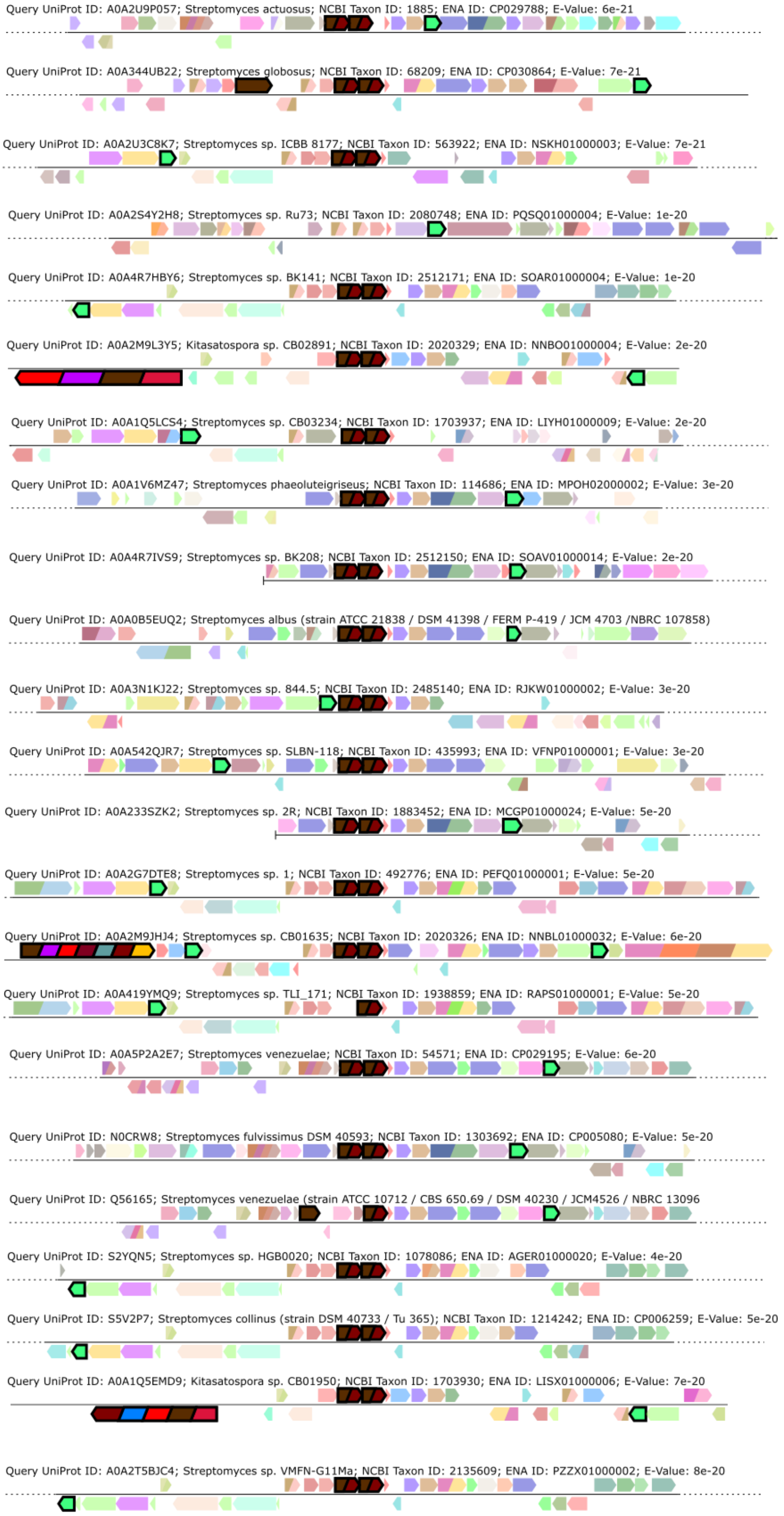
Query UniProt ID: A0A2U9P057; Streptomyces actuosus; NCBI Taxon ID: 1885; ENA ID: CP029788; E-Value: 6e-21
Query UniProt ID: A0A344UB22; Streptomyces globosus; NCBI Taxon ID: 68209; ENA ID: CP030864; E-Value: 7e-21
Query UniProt ID: A0A2U3C8K7; Streptomyces sp. ICBB 8177; NCBI Taxon ID: 563922; ENA ID: NSKH01000003; E-Value: 7e-21
Query UniProt ID: A0A2S4Y2H8; Streptomyces sp. Ru73; NCBI Taxon ID: 2080748; ENA ID: PQSQ01000004; E-Value: 1e-20
Query UniProt ID: A0A4R7HBY6; Streptomyces sp. BK141; NCBI Taxon ID: 2512171; ENA ID: SOAR01000004; E-Value: 1e-20
Query UniProt ID: A0A2M9L3Y5; Kitasatospora sp. CB02891; NCBI Taxon ID: 2020329; ENA ID: NNBO01000004; E-Value: 2e-20
Query UniProt ID: A0A1Q5LCS4; Streptomyces sp. CB03234; NCBI Taxon ID: 1703937; ENA ID: LIYH01000009; E-Value: 2e-20
Query UniProt ID: A0A1V6MZ47; Streptomyces phaeoluteigriseus; NCBI Taxon ID: 114686; ENA ID: MPOH02000002; E-Value: 3e-20
Query UniProt ID: A0A4R7IVS9; Streptomyces sp. BK208; NCBI Taxon ID: 2512150; ENA ID: SOAV01000014; E-Value: 2e-20
Query UniProt ID: A0A0B5EUQ2; Streptomyces albus (strain ATCC 21838 / DSM 41398 / FERM P-419 / JCM 4703 /NBRC 107858)
Query UniProt ID: A0A3N1KJ22; Streptomyces sp. 844.5; NCBI Taxon ID: 2485140; ENA ID: RJKW01000002; E-Value: 3e-20
Query UniProt ID: A0A542QJR7; Streptomyces sp. SLBN-118; NCBI Taxon ID: 435993; ENA ID: VFNP01000001; E-Value: 3e-20
Query UniProt ID: A0A233SZK2; Streptomyces sp. 2R; NCBI Taxon ID: 1883452; ENA ID: MCGP01000024; E-Value: 5e-20
Query UniProt ID: A0A2G7DTE8; Streptomyces sp. 1; NCBI Taxon ID: 492776; ENA ID: PEFQ01000001; E-Value: 5e-20
Query UniProt ID: A0A2M9JHJ4; Streptomyces sp. CB01635; NCBI Taxon ID: 2020326; ENA ID: NNBL01000032; E-Value: 6e-20
Query UniProt ID: A0A419YMQ9; Streptomyces sp. TLI_171; NCBI Taxon ID: 1938859; ENA ID: RAPS01000001; E-Value: 5e-20
Query UniProt ID: A0A5P2A2E7; Streptomyces venezuelae; NCBI Taxon ID: 54571; ENA ID: CP029195; E-Value: 6e-20
Query UniProt ID: N0CRW8; Streptomyces fulvissimus DSM 40593; NCBI Taxon ID: 1303692; ENA ID: CP005080; E-Value: 5e-20
Query UniProt ID: Q56165; Streptomyces venezuelae (strain ATCC 10712 / CBS 650.69 / DSM 40230 / JCM4526 / NBRC 13096
Query UniProt ID: S2YQN5; Streptomyces sp. HGB0020; NCBI Taxon ID: 1078086; ENA ID: AGER01000020; E-Value: 4e-20
Query UniProt ID: S5V2P7; Streptomyces collinus (strain DSM 40733 / Tu 365); NCBI Taxon ID: 1214242; ENA ID: CP006259; E-Value: 5e-20
Query UniProt ID: A0A1Q5EMD9; Kitasatospora sp. CB01950; NCBI Taxon ID: 1703930; ENA ID: LISX01000006; E-Value: 7e-20
Query UniProt ID: A0A2T5BJC4; Streptomyces sp. VMFN-G11Ma; NCBI Taxon ID: 2135609; ENA ID: PZZX01000002; E-Value: 8e-20

Figure S31. Gene clusters include PPTase-related genes. EFI-Genome Neighborhood Tool was used to build this scheme. SxrX was used as query protein and set E-value 1 and limit 10 neighbor genes on each side for BLAST. 188 gene clusters were found and 65 clusters containing SxrX homologue were displayed. Graphics was edited by Inkscape editor. The protein sequences and Genbank entries for all available PPTases were obtained from the NCBI database.

```
CLUSTAL O(1.2.4) multiple sequence alignment

TR|Q2MGB1|Q2MGB1_STRGR   --------------------------MQPDDEGA-------------------------- 8
SP|Q82DL2|ACPS_STRAW     ------------------------------------------------------------
SP|P96618|ACPS_BACSU     ------------------------------------------------------------
SP|P24224|ACPS_ECOLI     ------------------------------------------------------------
SP|O86785|ACPS_STRCO     ------------------------------------------------------------
SP|P0A2W7|ACPS_STRR6     ------------------------------------------------------------
TR|Q70DX0|Q70DX0_9ACTN   ------------------------------------------------------------
SxrX                     VIPRRVTLWQGRSEDRADTLAADAALLAPDELERLRRLRTPLAARRYAAAHAAVRRRLGA 60

TR|Q2MGB1|FdmW           -------------------REWRRGP------GGSGRFPWPLRLRARH--PRPAPPRIVG 41
SP|Q82DL2|ACPS_STRAW     -------------------------------------------------------MSIIG 5
SP|P96618|ACPS_BACSU     --------------------------------------------------------MIYG 4
SP|P24224|ACPS_ECOLI     -------------------------------------------------------MAILG 5
SP|O86785|ACPS_STRCO     -------------------------------------------------------MSIIG 5
SP|P0A2W7|ACPS_STRR6     --------------------------------------------------------MIVG 4
TR|Q70DX0|Q70DX0_9ACTN   -------------------------------------MSAAEALIAEA--LLNAPATVRG 21
SxrX                     VLDVDPVRVPLGRGPCPVCGERRHGPPVVRMPADGPALSYSLSRSGPHWMLAVAPGHP-- 118
TR|Q2MGB1|FdmW           VGIDVAAIARFGLALERS-PGLRDRLFTPEEQMLPSGS-P-RTTASLAARFAAKEAVAKV 98
SP|Q82DL2|ACPS_STRAW     VGIDVAEIDRFRASLERT-PGLADRLFLERELLLPNGE-R-RGIASLAARFAAKEAVAKA 62
SP|P96618|ACPS_BACSU     IGLDITELKRIASMAGRQ-KRFAERILTRSELDQYYELSEKRKNEFLAGRFAAKEAFSKA 63
SP|P24224|ACPS_ECOLI     LGTDIVEIARIEAVIARSGDRLARRVLSDNEWAIWKTH--HQPVRFLAKRFAVKEAAAKA 63
SP|O86785|ACPS_STRCO     VGIDVAEVERFGAALERT-PALAGRLFLESELLLPGGE-R-RGVASLAARFAAKEALAKA 62
SP|P0A2W7|ACPS_STRR6     HGIDIEELASIESAVTRH-EGFAKRVLTAQEMERFTSLKGRRQIEYLAGRWSAKEAFSKA 63
TR|Q70DX0|Q70DX0_9ACTN   VGIDVVDVQRLRAMLERRGRPLYDRLFTPAELVLCEGGSPRHRANRLAGRIAAKEAVRKS 81
SxrX                     VGVDLERERPLDV------DAVARAAFSARERRAVAGA---GGRALAFRCWTRKEAVAKA 169
                          * *:     :          .    :   *                    : ***  *

TR|Q2MGB1|FdmW           LGAP-G-GLRWHDVGVRTGARGRPVLQVCGTVAAAAARQGISVWHLSL-THDGDVASAVV 155
SP|Q82DL2|ACPS_STRAW     LGAP-G-GLYWTDAEVWVEDSGRPRLRVTGTVAARAAELGVQSWHVSL-SHDAGVASAVV 119
SP|P96618|ACPS_BACSU     FGTGIGRQLSFQDIEIRKDQNGKPYIIC--------TKLSQAAVHVSI-THTKEYAAAQV 114
SP|P24224|ACPS_ECOLI     FGTGIRNGLAFNQFEVFNDELGKPRLRLWGEALKLAEKLGVANMHVTL-ADERHYACATV 122
SP|O86785|ACPS_STRCO     LGAP-A-GLLWTDAEVWVEAGGRPRLRVTGTVAARAAELGVASWHVSL-SHDAGIASAVV 119
SP|P0A2W7|ACPS_STRR6     MGTGIS-KLGFQDLEVLNNERGAPYFSQ--------APFSG-KIWLSI-SHTDQFVTASV 112
TR|Q70DX0|Q70DX0_9ACTN   LGAH-GQGCGWLDVEIGRAESGQPLPRVSGRAEEAFRRASFTGLHLSI-THEAGLALAIA 139
SxrX                     TGHGIAMDLR--AVETRPRDPGPVRVAA--------------GWQVYSLFPGGSLHAAVA 213
                          *                   *                       :           * .

TR|Q2MGB1|FdmW           VGAA-------- 159
SP|Q82DL2|ACPS_STRAW     VAEG-------- 123
SP|P96618|ACPS_BACSU     VIERLSS----- 121
SP|P24224|ACPS_ECOLI     IIES-------- 126
SP|O86785|ACPS_STRCO     IAEG-------- 123
SP|P0A2W7|ACPS_STRR6     ILEENHES---- 120
TR|Q70DX0|Q70DX0_9ACTN   LAV--------- 142
SxrX                     VPEGEPGPVCRW 225
                         :
```

Figure S32. The sequence comparison of SxrX from *S. xanthophaeus* no2 with selected PPTases from *S. griseus* (Q2MGB1), *B. subtilis* (P96618), *E. coli* (P24224), *S. coelicolor* (O86785), *S. pneumoniae* (P0A2W7), and *S. resistomycificus* (Q70DX0). Given in parentheses are the protein accession numbers. The conserved motifs for PPTases are colored in yellow, and the distant N terminus of SxrX is colored in blue.

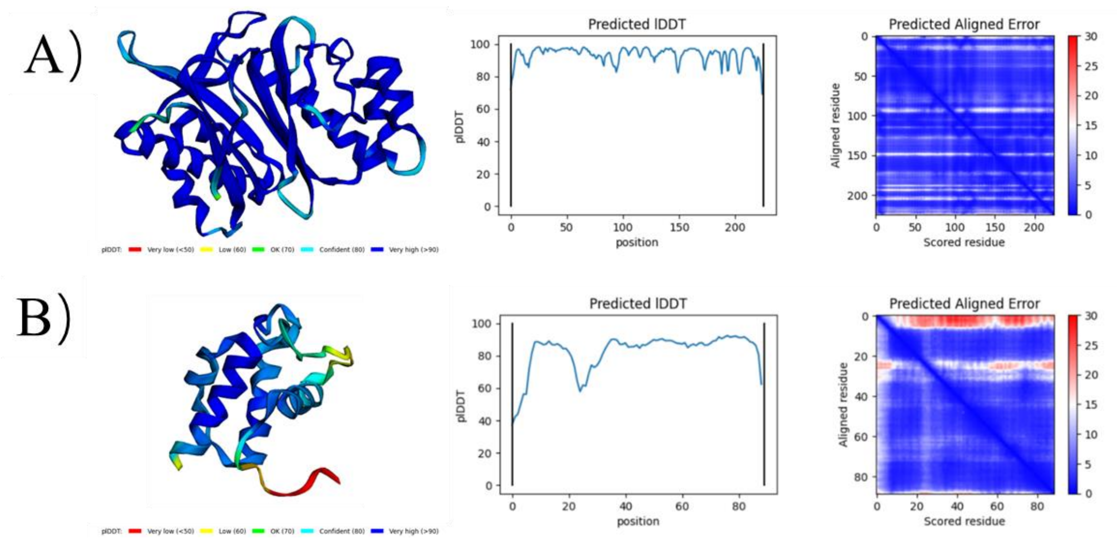

Figure S33. Modelling of SxrX and ACP SxrK1. (A) a physics-based model of SxrX generated using the AlphaFold v2.0 Google Colab notebook [6]. (B) a physics-based model of SxrK1 generated using the AlphaFold v2.0 Google Colab notebook [6].

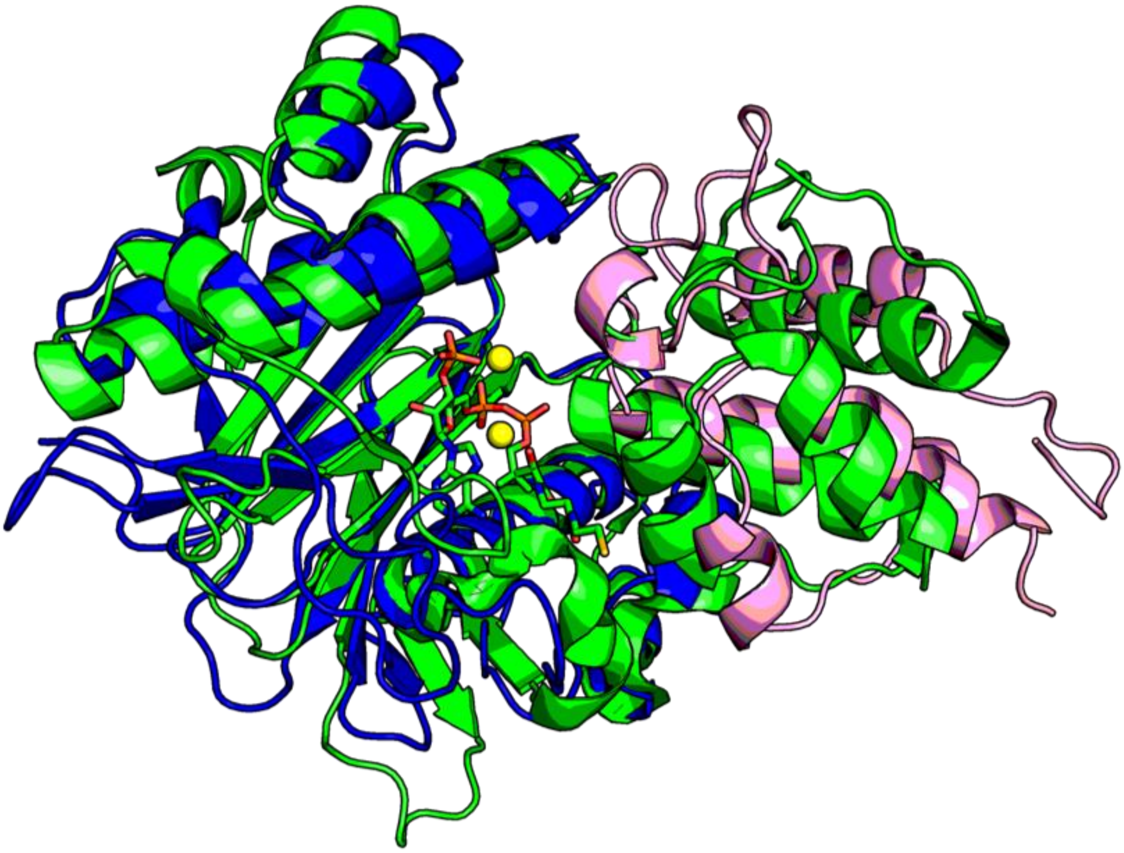

Figure S34. The Alignment of the three-dimensional structures of Sfp-PCP complex (PDB ID: 4MRT) and SxrX-SxrK1 complex. The Sfp-PCP complex, SxrX and SxrK1 are showed in green, blue and pink, respectively. The CoA is shown as sticks in green. The Mg^{2+} is shown as ball in yellow.

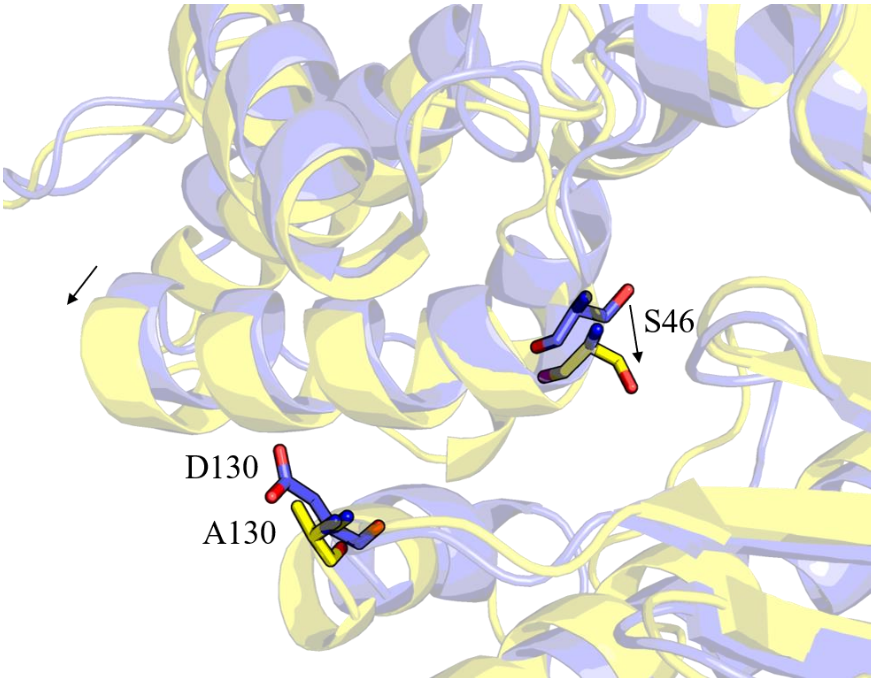

Figure S35. The Alignment of the structures optimized by MD simulation of SxrX-SxrK1 complex and its mutant. The SxrX-SxrK1 complex and D130A-SxrX are showed in blue and yellow, respectively. Black arrows denote the directions of movement.

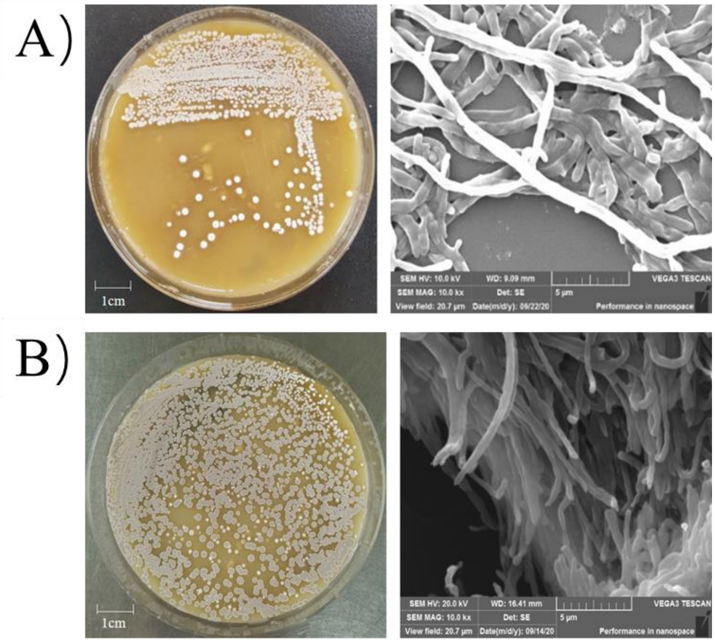

Figure S36. Phenotype characterization of *S. xanthophaeus* no2 and *S. xanthophaeus* no2 ΔsxrX. (A) Colony morphological characteristics and scanning electron microscope analysis of *S. xanthophaeus* no2; (B) Colony morphological characteristics and scanning electron microscope analysis of *S. xanthophaeus* no2 ΔsxrX.

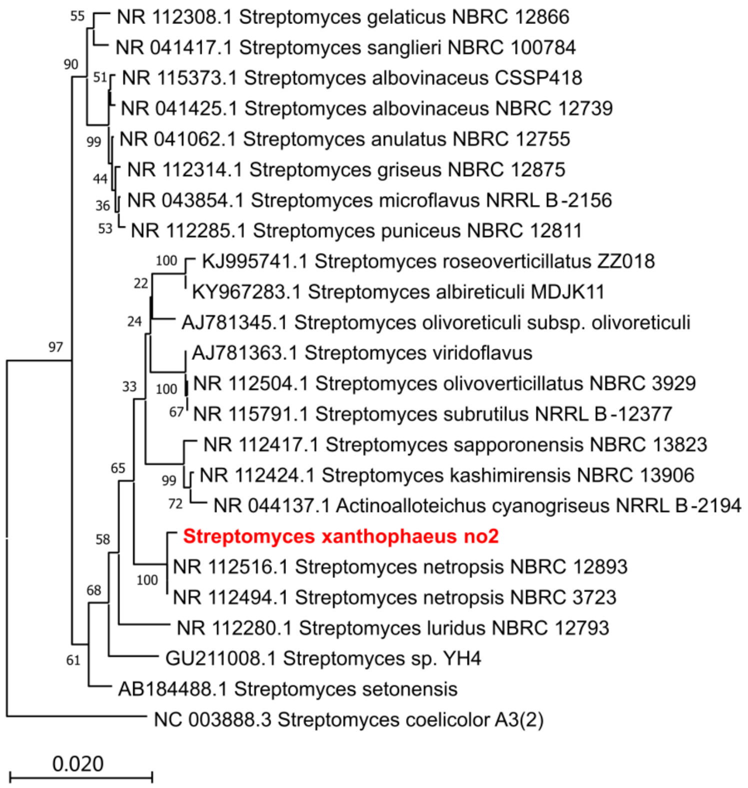

Figure S37. The phylogenetic relationship of *S. xanthophaeus* no2 with other bacterial strains based on 16S rRNA gene sequences. The phylogenetic tree was constructed using the neighbor joining method based on selected species belonging to the genus Streptomycetes. The bootstrap confidence value was obtained using 1000 re-samplings. The sequence alignments were performed in Clustal Omega and the phylogenetic trees were reconstructed by MEGA5.

References

1. Nathanael JG, Wille U. 2019. Oxidative damage in aliphatic amino acids and di- and tripeptides by the environmental free radical oxidant $NO_3^{\bullet}$: the role of the amide bond revealed by kinetic and computational studies. J Org Chem 84:3405–3418. https://doi.org/10.1021/acs.joc.8b03224.
2. Kawamura K, Kondo S, Maeda K, Umezawa H. 1969. Structures and syntheses of leupeptins Pr-LL and Ac-LL. Chem Pharm Bull 14:1902–1909. https://doi.org/10.1248/cpb.17.1902.
3. Zhang S, Klementz D, Zhu J, Makitrynskyy R, Ola Pasternak AR, Günther S, Zechel DL, Bechthold A. 2019. Genome mining reveals the origin of a bald phenotype and a cryptic nucleocidin gene cluster in *Streptomyces asterosporus* DSM 41452. J Biotechnol 292:23–31. https://doi.org/10.1016/j.jbiotec.2018.12.016.
4. Datsenko KA, Wanner BL. 2000. One-step inactivation of chromosomal genes in Escherichia coli K-12 using PCR products. Proc Natl Acad Sci U S A 97:6640–6645. https://doi.org/10.1073/pnas.120163297.
5. Li JH, Oh J, Kienesberger S, Kim NY, Clarke DJ, Zechner EL, Crawford JM. 2020. Making and breaking leupeptin protease inhibitors in pathogenic gammaproteobacteria. Angew Chem Int Ed Engl 59:17872–17880. https://doi.org/ 10.1002/anie.202005506.
6. Jumper J, Evans R, Pritzel A, Green T, Figurnov M, Ronneberger O, Tunyasuvunakool K, Bates R, Žídek A, Potapenko A, Bridgland A, Meyer C, Kohl SAA, Ballard AJ, Cowie A, Romera-Paredes B, Nikolov S, Jain R, Adler J, Back T, Petersen S, Reiman D, Clancy E, Zielinski M, Steinegger M, Pacholska M, Berghammer T, Bodenstein S, Silver D, Vinyals O, Senior AW, Kavukcuoglu K, Kohli P, Hassabis D. 2021. Highly accurate protein structure prediction with AlphaFold. Nature 596:583–589. https://doi.org/10.1038/s41586-021-03819-2.
